# Complex Genomic Landscape of Inversion Polymorphism in Europe's Most Destructive Forest Pest

**DOI:** 10.1093/gbe/evae263

**Published:** 2024-12-04

**Authors:** Anastasiia Mykhailenko, Piotr Zieliński, Aleksandra Bednarz, Fredrik Schlyter, Martin N Andersson, Bernardo Antunes, Zbigniew Borowski, Paal Krokene, Markus Melin, Julia Morales-García, Jörg Müller, Zuzanna Nowak, Martin Schebeck, Christian Stauffer, Heli Viiri, Julia Zaborowska, Wiesław Babik, Krystyna Nadachowska-Brzyska

**Affiliations:** Institute of Environmental Sciences, Faculty of Biology, Jagiellonian University, 30-387 Kraków, Poland; Doctoral School of Exact and Natural Sciences, Jagiellonian University, 30-348 Kraków, Poland; Institute of Environmental Sciences, Faculty of Biology, Jagiellonian University, 30-387 Kraków, Poland; Institute of Environmental Sciences, Faculty of Biology, Jagiellonian University, 30-387 Kraków, Poland; Chemical Ecology, Department of Plant Protection Biology, Swedish University of Agricultural Sciences Alnarp, 234 22 Lomma, Sweden; ETM, Faculty of Forestry and Wood Sciences, Czech University of Life Sciences Prague, 165 00 Praha, Czechia; Department of Biology, Lund University, 223 62 Lund, Sweden; Institute of Environmental Sciences, Faculty of Biology, Jagiellonian University, 30-387 Kraków, Poland; Departament of Forest Ecology, Forest Research Institute, 05-090 Raszyn, Poland; Division of Biotechnology and Plant Health, Norwegian Institute of Bioeconomy Research, 1433 Ås, Norway; Forest Health and Bidiversity Group, Natural Resources Institute Finland, 80100 Joensuu, Finland; Institute of Environmental Sciences, Faculty of Biology, Jagiellonian University, 30-387 Kraków, Poland; Doctoral School of Exact and Natural Sciences, Jagiellonian University, 30-348 Kraków, Poland; Field Station Fabrikschleichach, Animal Ecology and Tropical Biology, Biocenter, University of Würzburg, 96181 Rauhenebrach, Germany; Bavarian Forest National Park, 94481 Grafenau, Germany; Institute of Environmental Sciences, Faculty of Biology, Jagiellonian University, 30-387 Kraków, Poland; Institute of Forest Entomology, Forest Pathology and Forest Protection, Department of Forest and Soil Sciences, University of Natural Resources and Life Sciences Vienna (BOKU), 1190 Vienna, Austria; Institute of Forest Entomology, Forest Pathology and Forest Protection, Department of Forest and Soil Sciences, University of Natural Resources and Life Sciences Vienna (BOKU), 1190 Vienna, Austria; UPM Forest, UPM-Kymmene, 33100 Tampere, Finland; Institute of Environmental Sciences, Faculty of Biology, Jagiellonian University, 30-387 Kraków, Poland; Institute of Environmental Sciences, Faculty of Biology, Jagiellonian University, 30-387 Kraków, Poland; Institute of Environmental Sciences, Faculty of Biology, Jagiellonian University, 30-387 Kraków, Poland

**Keywords:** polymorphic inversions, genome complexity, spruce bark beetle, *Ips typographus*, forest pest

## Abstract

In many species, polymorphic genomic inversions underlie complex phenotypic polymorphisms and facilitate local adaptation in the face of gene flow. Multiple polymorphic inversions can co-occur in a genome, but the prevalence, evolutionary significance, and limits to complexity of genomic inversion landscapes remain poorly understood. Here, we examine genome-wide genetic variation in one of Europe's most destructive forest pests, the spruce bark beetle *Ips typographus*, scan for polymorphic inversions, and test whether inversions are associated with key traits in this species. We analyzed 240 individuals from 18 populations across the species' European range and, using a whole-genome resequencing approach, identified 27 polymorphic inversions covering ∼28% of the genome. The inversions vary in size and in levels of intra-inversion recombination, are highly polymorphic across the species range, and often overlap, forming a complex genomic architecture. We found no support for mechanisms such as directional selection, overdominance, and associative overdominance that are often invoked to explain the presence of large inversion polymorphisms in the genome. This suggests that inversions are either neutral or maintained by the combined action of multiple evolutionary forces. We also found that inversions are enriched in odorant receptor genes encoding elements of recognition pathways for host plants, mates, and symbiotic fungi. Our results indicate that the genome of this major forest pest of growing social, political, and economic importance harbors one of the most complex inversion landscapes described to date and raise questions about the limits of intraspecific genomic architecture complexity.

**Significance**

The spruce bark beetle (*Ips typographus*) is one of the most destructive forest pests, causing mass mortality of spruce stands under favorable weather conditions. Here, we performed a large-scale population genomic analysis and provided information on the genetic structuring and variation along the species genome. We found that the spruce bark beetle harbors one of the most complex polymorphic inversion landscapes described to date. We also found that inversions are enriched in odorant receptor genes, which are important for recognizing hosts, mates, and symbiotic fungi. Our study raises questions about the limits of the complexity of intraspecific genome rearrangements and the causes and consequences of inversion-rich genomes. Our findings not only shed light on the genomic variation of the major forest pest but also contribute to the understanding of the abundance of inversion polymorphisms that are increasingly found across the tree of life.

## Introduction

Large structural variants (SVs), such as chromosomal inversions, translocations, insertions, and duplications, were among the earliest mutations to be described in natural populations (McClung 1905; Sturtevant 1921; Dobzhansky and Sturtevant 1938; Dobzhansky 1970), but their systematic identification and in-depth analysis have only become possible with recent advances in sequencing technology (Wellenreuther et al. 2019). The increasing accumulation of genomic data has revealed not only the presence of structural variation between and within many species but also its important role in species adaptation and speciation (Lamichhaney et al. 2015; Van't Hof et al. 2016; Cheng et al. 2018; Wellenreuther and Bernatchez 2018; Faria, Chaube, et al. 2019; Todesco et al. 2020; Mérot et al. 2021; Harringmeyer and Hoekstra 2022; Saitou et al. 2022). One particular form of SVs, polymorphic chromosomal inversions, has been at the center of recent debate about their potential to influence the evolutionary process (Berdan et al. 2023).

Polymorphic inversions are chromosomal segments that occur in two orientations within populations: collinear and inverted haplotypes/arrangements. Inversions have been shown to be involved in speciation, local adaptation, and maintenance of complex phenotypes (Lamichhaney et al. 2015; Lohse et al. 2015; Wellenreuther and Bernatchez 2018; Fuller et al. 2019). This is due to a key property of inversions: they suppress recombination within heterozygotes and thereby prevent the separation of alleles present in each inversion haplotype. Because of their role as recombination modifiers, inversions can act as supergenes—large elements of genomic architecture containing multiple linked functional elements (Thompson and Jiggins 2014). Supergenes keep alleles together in the face of gene flow and suppress the formation of recombinant genotypes.

Classic examples of supergenes include inversions associated with different mating strategies in ruffs (*Calidris pugnax*; Lamichhaney et al. 2015), mimicry phenotypes in *Heliconius* butterflies (Joron et al. 2011), and social organization in fire ants (*Solenopsis* spp.; Purcell et al. 2014; Gutiérrez-Valencia et al. 2021). In many other species, polymorphic inversions define locally adapted ecotypes (Lowry and Willis 2010; Kirubakaran et al. 2016; Koch et al. 2021; Matschiner et al. 2022; Reeve et al. 2023) or exhibit spatial haplotype frequency differences, e.g. by forming geographic and climatic gradients in inversion distributions (Ayala et al. 2014, 2017; Kapun et al. 2016; Mérot et al. 2021). While most of the described cases are organisms with one or a few inversions, several recent studies have reported species with many polymorphic inversions (*Littorina* snails, Faria, Chaube, et al. 2019; Reeve et al. 2023; sunflowers, Todesco et al. 2020; deer mice, Harringmeyer and Hoekstra 2022; humans, Porubsky et al. 2022). These recent findings raise questions about the prevalence and evolutionary significance of polymorphic inversions in natural populations. Are inversion-rich genomes the exception or the rule? How much of the genome can be situated within polymorphic inversions and, consequently, how large can the fraction of the genome that undergoes reduced recombination be? The latter question is particularly important because, in addition to suppressing recombination and keeping coadapted alleles together, inversion heterozygotes also suppress the formation of new allelic combinations and thus reduce the efficacy of natural selection (Roesti et al. 2022).

Long-term persistence of two inversion haplotypes can be facilitated by both divergent and balancing selection (Faria, Johannesson, et al. 2019). Divergent selection can favor different inversion genotypes in different environments and, when coupled with reduced migration between divergent populations, can lead to speciation. Even when intraspecific gene flow is high, divergent selection can lead to divergent ecotypes associated with locally advantageous inversion genotypes. Alternatively, balancing selection may maintain balanced inversion polymorphisms over time via several, not mutually exclusive, mechanisms, such as overdominance, negative frequency dependence, antagonistic pleiotropy, and spatially or temporally varying selection (Connallon and Clark 2014; Faria, Johannesson, et al. 2019). Importantly, regardless of the selection mechanism, inversions will accumulate mutations independently since recombination is suppressed in inversion heterozygotes. This will lead to differentiated allelic content and increased differentiation between inversion haplotypes over time (Faria, Johannesson, et al. 2019). Each inversion haplotype can thus be treated as a separate “population” with a size that corresponds to the frequency of that arrangement within the studied population or species. Rare inversion arrangements will experience a high mutational load due to reduced recombination and limited purging because there are few homozygotes. However, deleterious mutations can also accumulate on more frequent inversion haplotypes (Berdan et al. 2021) and lead to associative overdominance. This helps to maintain inversion polymorphisms since independent accumulation of mutations continues over time, but recessive deleterious alleles private to an inversion arrangement are invisible to selection in inversion heterozygotes.

The Eurasian spruce bark beetle (*Ips typographus* [L.]: Curculionidae: Scolytinae; hereafter the spruce bark beetle), plays a key role in Eurasian forest ecosystems. Under endemic conditions, it attacks weakened Norway spruce (*Picea abies* [L.] H. Karst.) trees. However, if spruce resistance is compromised by certain abiotic disturbances (e.g. snowbreaks, windfalls, high temperatures, and drought), an increased availability of stressed trees can trigger mass propagation of beetles and lead to rapid population increase and devastating outbreaks. The extent of recent beetle outbreaks in Europe is unprecedented, and impacts will likely increase in the coming decades in response to climate change (Biedermann et al. 2019; Bentz et al. 2021; Müller et al. 2022). For example, during the first decade of this century, the spruce bark beetle killed an estimated 14.5 million m^3^ of timber per year on average. The Czech Republic provides a particularly striking example of the beetles' destructive potential. During the peak outbreak years of 2017 to 2019, the beetles killed 3.1% to 5.4% of the country's entire growing stock of Norway spruce each year, translating to 23 million m^3^ spruce killed in 2019. Historically, Central Europe has been most heavily affected by bark beetle outbreaks, while outbreaks have been less frequent and destructive in northern Europe (Hlásny et al. 2019). However, this may change as climate warming is expected to make the boreal forests of Northern Europe more vulnerable to bark beetle outbreaks (Lange et al. 2006; Jönsson et al. 2007; Bentz et al. 2021; Müller et al. 2022). As an example, heatwaves and severe summer drought in Sweden in 2018 initiated a bark beetle outbreak killing over 30 million m^3^ Norway spruce in the next years (Öhrn et al. 2021).

Increasing bark beetle attacks and other forest disturbances have already triggered social and political conflicts in parts of Europe and have highlighted the urgent need for improved management strategies (Vega and Hofstetter 2014; Hlásny et al. 2021). The increasing importance of the spruce bark beetle is reflected in a rapidly growing body of research focuses on the species' ecology and the causes and consequences of outbreaks (reviewed in Vega and Hofstetter 2014; Biedermann et al. 2019; Hlásny et al. 2021). Despite this enormous interest, one aspect of the species' biology remains largely unexplored: we know almost nothing about the species' genome-wide variation and the evolutionary mechanisms that shape this variation. The lack of population genomics studies that analyze whole-genome variation restricts our understanding of the genomic basis of adaptation and adaptive potential in the spruce bark beetle. This is particularly important because, as revealed in this study, the spruce bark beetle genome harbors a complex inversion polymorphism landscape that may play a critical role in many evolutionary processes, including adaptation (discussed above).

Here, we investigated genome-wide variation across spruce bark beetle populations with a special focus on chromosomal inversion polymorphisms. We found one of the most complex polymorphic inversion architectures described to date and investigated several evolutionary mechanisms, including directional selection, overdominance, and associative overdominance, that can maintain inversion polymorphisms in the genome. We also tested associations between inversion polymorphisms and two key fitness-related traits in the spruce bark beetle—diapause and olfaction. Our results suggest that inversions may play a role in the evolution of key traits in this species and raise questions about the prevalence and role of inversion polymorphisms in species characterized by large effective population sizes, little geographic subdivision, and no clear phenotypic variation across their geographical range.

## Results and Discussion

**Fig. 1.**
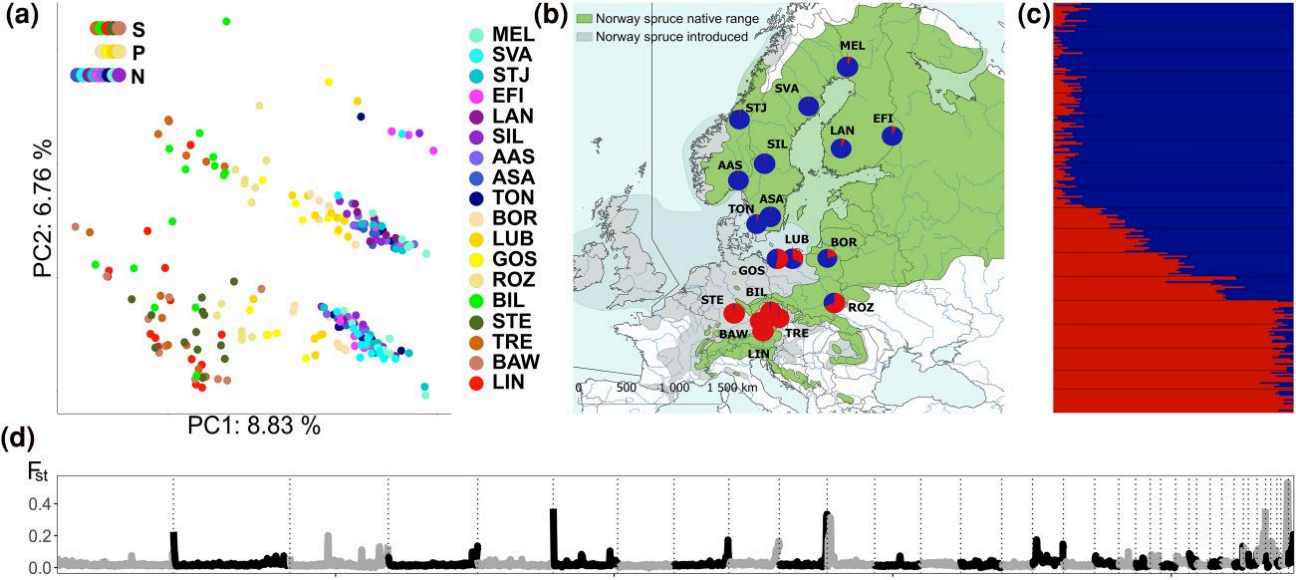
Genomic structure and differentiation in *I. typographus*. a) Whole-genome PCA, where colors correspond to what population an individual beetle belongs to. Colors indicate geographical grouping of populations: S, south; P, Poland; N, north. b, c) Geographical distribution and genetic differentiation of the 18 beetle populations analyzed (see supplementary table S11, Supplementary Material online for details about the sampling locations). The two main genetic groups are shown in red (southern) and blue (northern). d) Genome-wide genetic differentiation (*F*_ST_) calculated between northern and southern groups. Vertical lines separate different contigs (shown in gray and black). All panels were prepared using data that included inversions.

We resequenced whole genomes of the spruce bark beetle from 18 European populations (Fig. 1; supplementary table S1, Supplementary Material online). After initial raw data processing from 253 individuals (including data quality check, trimming and Genome Analysis Toolkit (GATK)-guided variant calling, genotyping, recalibration, and filtering; for details, see Supplementary material), we used 240 individuals (141 females and 99 males) in downstream analyses. The mean per individual sequencing depth was 23.2× (range: 5 to 53×). Sequencing coverage on IpsContig9 was consistently lower in individuals sexed as males (on average 0.57 individual coverage). Thus, we considered IpsContig 9 to be a sex (X) chromosome (females carry XX and males XYp chromosomes). After quality filtering, we retained 5.245 million single nucleotide polymorphisms (SNPs) covering the entire length of the genome assembly (236.8 Mb) but analyzed a subset of 5.067 million SNPs located on the 36 longest contigs and representing 78% of the assembly. Alignment of the *I. typographus* and *Ips nitidus* genome assemblies indicated no major misassembly problems (*I. typographus* contigs align to single *I. nitidus* chromosomes) and demonstrated high synteny between karyotypes of both species (supplementary fig. S1, Supplementary Material online). Given these results on contig sizes, genome assembly quality scores, and species karyotypes, we find it likely that many large spruce bark beetle contigs represent entire chromosome arms. Such a high level of genome quality is sufficient for all downstream analyses, in particular SNP-based polymorphic inversion identification (Mérot et al. 2021; Reeve et al. 2023).

### Complex Genomic Inversion Landscape

**Fig. 2.**
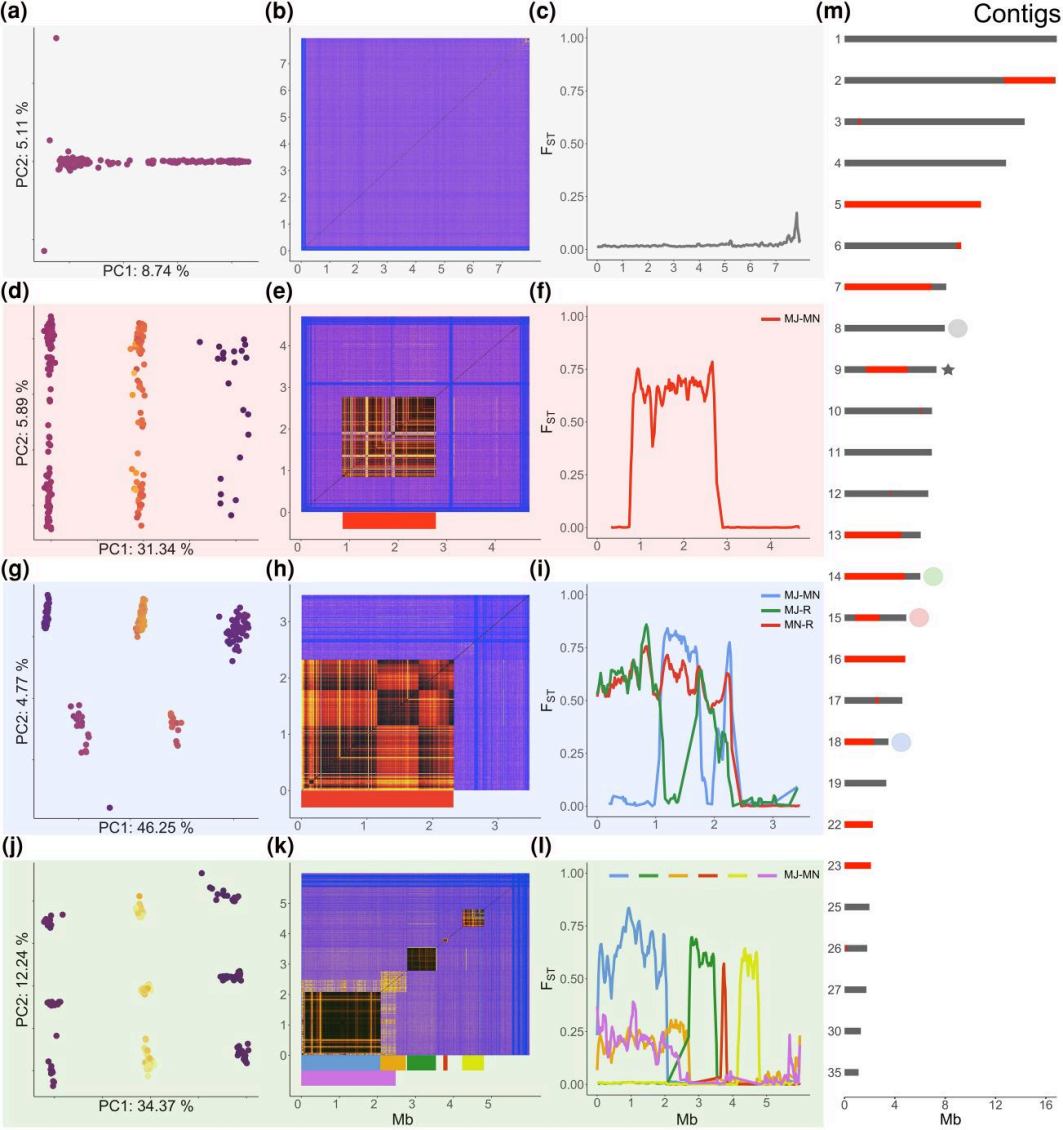
Identification of chromosomal inversions in *I. typographus*. Each row of figure panels shows the results of per-contig PCA a, d, g, j), per-contig LD analysis b, r, h, k), and genetic differentiation (*F*_ST_) analysis for a selected contig c, f, i, l). Dots in the PCA panels represent individual beetles and are colored according to the heterozygosity of the individual (darker colors represent lower heterozygosity). a to c) Results for a contig with no inversions (IpsContig8) and little differentiation between southern and northern populations (c, *F*_ST_ between southern and northern populations). d to l) Different contigs with increasingly complex inversion patterns: a single inversion (d to f, Inv15); a single inversion with a double-crossover signal (g to i, Inv18); and multiple adjacent inversions with one inversion overlapping with the first two inversions on the contig (j to l; Inv14.1, Inv14.2, Inv14.3, Inv14.4, Inv14.5, and Inv.14.6). m) Contigs with inversions indicated in red (contigs for which inversion genotyping was not possible are not shown; see Materials and Methods section for details). Colored dots indicate the inversions that are presented in detail in a) to l). Colored bars e, h, and k) indicate the position of each inversion. The same colors are used in f), i), and l), where *F*_ST_ is calculated between major (MJ) and minor (MN) inversion haplotypes. Two additional *F*_ST_ lines (blue and green) in i) show *F*_ST_ calculated between inversion homozygotes and a recombinant haplotype (R) formed after a putative double-crossover event. Both axes in b), e), h), and k) represent positions along the contig in megabases; low levels of linkage are shown in blue and higher levels in yellow to dark red. Sex chromosome is indicated with a star in m).

Twenty-nine candidate inversions (“inversions” henceforth) were identified following the criteria described in the Materials and Methods section (Table 1; Fig. 2; supplementary figs. S2 and S3, Supplementary Material online). Briefly, we considered a genomic region to harbor a polymorphic inversion if local principal component analysis (PCA) identified the region as an outlier and/or the region exhibited high linkage disequilibrium (LD) and PCA performed on SNPs from this region separated individuals not by geography but into three distinct groups (with one group classified as putative heterozygous individuals characterized by higher heterozygosity compared to the other two groups). Two putative inversions (Inv16.1 and Inv23.1) were most likely part of the same inversion: they are in strong LD (supplementary fig. S4, Supplementary Material online) and the same beetles were always genotyped as homo- and heterozygotes in both, which is unlikely to occur for two unlinked inversions. The same situation was found for Inv16.2 and Inv23.2. No other inversions were in strong LD with each other, which would suggest cosegregation (supplementary fig. S4, Supplementary Material online). Thus, overall, we found 27 inversions in 17 contigs, including one located on the X chromosome (Inv9). Approximate inversion sizes varied from 0.1 to 10.8 Mb (Table 1) and inversions constituted ∼28% of the analyzed part of the genome (48 Mb of 170 Mb) and at least 18.6% of the entire assembly. Estimated inversion ages ranged from 0.5 to 2.6 My (Table 1, assuming a mutation rate of 2.9 × 10^−9^; see supplementary table S1, Supplementary Material online for results for different mutation rates), and younger inversions had higher major inversion haplotype frequency (supplementary fig. S5, Supplementary Material online). Inversion regions exhibited a reduced population recombination rate in heterozygous individuals (supplementary fig. S2, Supplementary Material online) and moderate to high genetic differentiation between inversion arrangements (*F*_ST_ between homozygous individuals was 0.15 to 0.64; Fig. 1; supplementary fig. S2, Supplementary Material online).

**Table 1:** Identified chromosomal inversions in the *I. typographus* genome

ID	Contig	Size	Start	End	Age	Odorant receptors
Inv2	IpsContig2	4.04	12.67	16.71	0.5	…
Inv3	IpsContig3	0.14	1.11	1.25	1.1	…
Inv5	IpsContig5	10.84	0.00	10.84	1.3	ItypOR33, ItypOR41, ItypOR40, ItypOR10, ItypOR47, ItypOR50, ItypOR29, ItypOR43JOI, ItypOR34, ItypOR52NTE, ItypOR4, ItypOR3, ItypOR53, ItypOR2, ItypOR19
Inv6	IpsContig6	0.34	8.93	9.27	1.0	…
Inv7.1	IpsContig7	0.67	0.00	0.67	1.7	…
Inv7.2	IpsContig7	6.92	0.00	6.92	1.1	ItypOR1, ItypOR17
Inv9	IpsContig9	3.30	1.71	5.01	0.6	…
Inv10	IpsContig10	0.08	6.05	6.13	1.8	…
Inv12	IpsContig12	0.07	3.63	3.70	1.5	…
Inv13	IpsContig13	4.50	0.00	4.50	1.7	ItypOR28, ItypOR23, ItypOR49, ItypOR27
Inv14.1	IpsContig14	2.08	0.00	2.08	1.9	ItypOR36, ItypOR44, ItypOR18JF, ItypOR20NTE
Inv14.2	IpsContig14	0.67	2.08	2.75	1.0	…
Inv14.3	IpsContig14	0.76	2.78	3.54	2.1	…
Inv14.4	IpsContig14	0.11	3.73	3.84	2.1	…
Inv14.5	IpsContig14	0.57	4.23	4.80	2.3	…
Inv14.6	IpsContig14	2.48	0.00	2.48	0.6	ItypOR36, ItypOR44, ItypOR18JF, ItypOR20NTE
Inv15	IpsContig15	1.92	0.86	2.78	1.9	…
Inv16.1	IpsContig16	4.83	0.00	4.83	1.6	ItypOR58, ItypOR9, ItypOR11, ItypOR31, ItypOR30, ItypOR16, ItypOR35JF
Inv16.2	IpsContig16	4.83	0.00	4.83	0.7	ItypOR58, ItypOR9, ItypOR11, ItypOR31, ItypOR30, ItypOR16, ItypOR35JF
Inv17	IpsContig17	0.21	2.48	2.69	2.2	…
Inv18	IpsContig18	2.32	0.00	2.32	2.1	…
Inv22.1	IpsContig22	0.32	0.00	0.32	2.1	…
Inv22.2	IpsContig22	0.23	0.42	0.65	2.1	…
Inv22.3	IpsContig22	1.23	0.68	1.91	2.0	ItypOR22CTE
Inv22.4	IpsContig22	0.20	1.92	2.12	2.1	…
Inv22.5	IpsContig22	2.24	0.00	2.24	0.6	ItypOR22CTE
Inv23.1	IpsContig23	2.10	0.00	2.10	1.4	ItypOR58, ItypOR9
Inv23.2	IpsContig23	1.84	0.26	2.10	0.6	ItypOR58, ItypOR9
Inv26	IpsContig26	0.10	0.12	0.22	2.6	…

Note that Inv16.1 and Inv23.1 are parts of the same inversion and Inv16.2 and Inv23.2 are a part of another single inversion.

ID, inversion name; Contig, contig name; Size, size of the inversions (Mb); Start and End, coordinates of the inversion (Mb); Age, approximate age of the inversion in Myr; Odorant receptors, odorant receptors present within inversion (in the order they appear along the contig sequence).

While the variation patterns observed on contigs with single inversions (12 contigs) were clear and left no doubt about the presence of polymorphic inversions, we also observed more complex patterns such as overlapping inversions and inversions with double-crossover events. These patterns were more difficult to interpret and deserve additional explanation and caution (Fig. 2; supplementary figs. S2 and S3, Supplementary Material online).

Putative overlapping inversions were identified on the basis of overlapping clusters of high LD, PCA clustering into three distinct groups along PC2 or PC3 (in addition to the three distinct groups along PC1), and moderate to high *F*_ST_ between individuals classified as alternative homozygotes (Fig. 2; supplementary fig. S3, Supplementary Material online). In summary, IpsContig14 and IpsContig22 contained complexes of multiple adjacent and sometimes overlapping inversions (Fig. 2j to l; supplementary figs. S2 and S3, Supplementary Material online), and three other contigs contained two overlapping inversions each (IpsContigs: 7, 16, and 23; supplementary figs. S2 and S3, Supplementary Material online). In the case of putative double-crossover events, the observed LD clusters were separated by regions of lower LD and intermediate groups of individuals were visible between the main clusters along PC1 (supplementary fig. S3, Supplementary Material online). The *F*_ST_ between individuals classified as alternative homozygotes was significantly reduced in the region of the putative double crossover (Fig. 2; supplementary figs. S2 and S3, Supplementary Material online). In total, four putative double-crossover events were identified within four inversions (Inv5, Inv18, Inv22.3, and Inv22.4; Fig. 1; supplementary figs. S2 and S3, Supplementary Material online).

The most difficult patterns to disentangle were associated with Inv2, Inv5, Inv7.1, and Inv7.2. Putative Inv2 showed a signature of overlapping LD clusters (overlapping inversions) but ambiguous PCA clustering (supplementary fig. S3, Supplementary Material online). The patterns were clear and consistent with inversion polymorphisms when only populations from the southern region were analyzed (supplementary fig. S6, Supplementary Material online; thus, Inv2 was genotyped only in this part of the species range). This suggests the existence of other structural differences between the southern and northern regions or unidentified genome assembly problems. PCA of Inv5 identified three clusters that most likely correspond to three inversion genotypes, but it also identified several smaller clusters. The smaller clusters may indicate multiple independent recombination events (double-crossover events that occurred at different locations within Inv5; supplementary figs. S2 and S3, Supplementary Material online) or assembly problems within Inv5 (unlikely, since IpsContig5 aligns perfectly with *I. nitidus* chromosome 9; supplementary fig. S1, Supplementary Material online). Thus, Inv5 was only genotyped for three major genotype groups for which multiple lines of evidence (LD, heterozygosity, and *F*_ST_) suggested an inversion polymorphism. Inv7.1 and Inv7.2 are characterized by overlapping LD clusters, and PCA and *F*_ST_ patterns consistent with overlapping inversions. However, Inv7.2 showed low LD in the middle of the inversion and unexpected clustering of individuals in one of the clusters along PC1. Here, the cluster was split into two distinct groups where individuals clustered according to their geographic location within each group (supplementary figs. S2 and S3, Supplementary Material online). This suggests possible misassembly, and Inv7.2 is probably shorter than predicted from the size of the LD regions. Genotyping of Inv16.2 and Inv23.2 was only possible for part of the species range, as PCA clustering into three genotype groups was only visible when analyzing the northern group (supplementary fig. S6, Supplementary Material online).

Even though we cannot rule out that some of the inversion patterns we identified were formed or distorted by misassembly or additional structural rearrangements (e.g. duplications; Kim et al. 2022), there is strong evidence for most of the polymorphic inversions in the spruce bark beetle. Extensive collinearity with *I. nitidus* suggested no major misassembly problems and supports our identification of polymorphic inversions in the spruce bark beetle genome (supplementary fig. S1, Supplementary Material online; Wang et al. 2023). Nevertheless, long-read sequencing and Hi-C data are needed to shed light on the few problematic/complex cases and to precisely identify the inversion boundaries. It was not surprising that we detected polymorphic inversions in the spruce bark beetle genome, as there are many well-known examples of polymorphic inversions in natural populations (Wellenreuther and Bernatchez 2018). What was striking, however, was the complexity of the genomic landscape of polymorphic inversions we found in this species. The spruce bark beetle has at least 27 large inversions covering a substantial part of the genome (28% of the analyzed part of the genome). Numerous (a dozen or more) polymorphic inversions have been described for several species (e.g. Faria, Chaube, et al. 2019; Tigano et al. 2021; Harringmeyer and Hoekstra 2022), but it remains an open question whether many polymorphic inversions within species are the exception or the rule.

The exceptionally complex inversion architecture we found in the spruce bark beetle, with multiple adjacent and often overlapping inversions, resembles well-known examples from *Heliconius* butterflies (Jay et al. 2021) and fire ants (Wang et al. 2013). In these insects, multiple adjacent inversions are the basis for mimicry phenotypes and complex social organization, respectively. The presence of clusters of adjacent inversions and inversion overlaps are consistent with theoretical expectations of stepwise extension of recombination suppression on supergenes (Jay et al. 2022) and with a highly polygenic architecture of adaptation (Schaal et al. 2022).

### Genome-Wide Variation versus Inversion Region Variation and Its Geographic Structure

Analyses based on the whole genome (including inversions and collinear regions) revealed a clear latitudinal structuring of genetic variation in the spruce bark beetle (Fig. 1). NGSadmix supported the presence of two distinct genetic groups corresponding to southern and northern populations, with Polish populations showing varying degrees of admixture between the two groups (supplementary fig. S7, Supplementary Material online). Based on these results, and to be able to assess the differentiation between two genetic clusters, we divided the studied populations into a northern and southern group, excluding admixed Polish populations. Despite unambiguous NGSadmix division into two genetic clusters, the genome-wide genetic differentiation between the northern and southern groups was very low (*F*_ST_ = 0.021). This was true also within inversion regions where *F*_ST_ ranged from 0.005 to 0.05. Similarly, pairwise *F*_ST_ between populations showed low levels of differentiation, ranging from 0.000 to 0.035 (calculated excluding inversions; supplementary table S2, Supplementary Material online). Mean genome-wide nucleotide diversity was moderate (*π* = 0.0062) and per population *π* ranged from 0.0055 to 0.0066 (supplementary fig. S8, Supplementary Material online). There was a weak negative correlation between nucleotide diversity and latitude (*r*^2^ = 0.32, *P* = 0.033; supplementary fig. S9, Supplementary Material online), as northern populations had slightly lower genetic variation than southern populations (*π*_southern_ = 0.0065; *π*_northern_ = 0.0061; *π*_Polish_ = 0.0066). Southern populations had an excess of rare alleles and, consequently, had more negative Tajima's *D*-values along the genome than northern populations (mean Tajima's *D* was −0.458 and −0.062 in the southern and northern groups, respectively; supplementary fig. S8, Supplementary Material online). All these results are consistent with previous phylogeographic studies of the spruce bark beetle that analyzed a much smaller number of genetic markers. The data suggests high levels of connectivity among populations and a very recent differentiation into two genetic clusters (Stauffer et al. 1999; Sallé et al. 2007; Bertheau et al. 2013; Mayer et al. 2015). More recent RADseq data confirm a very weak genetic structure in the spruce bark beetle across much of Sweden (Ellerstrand et al. 2022), as is expected in a species with high dispersal (Nilssen 1984) and/or recent divergence between populations. Tajima's *D*-values indicate a different demographic history of the southern and northern groups (e.g. recent demographic expansion of southern populations and more stable demography in northern populations; supplementary fig. S8, Supplementary Material online).

**Fig. 3.**
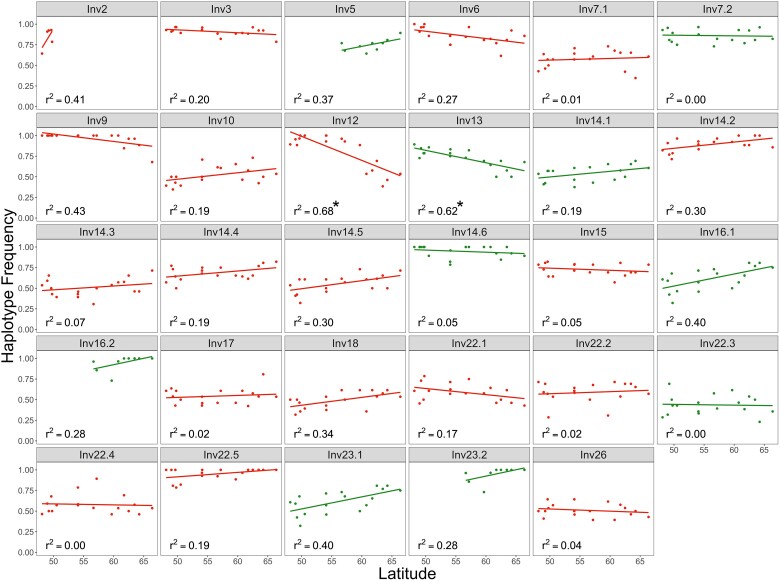
Correlation between inversion haplotype frequency and latitude for chromosomal inversions detected in European *I. typographus* populations sampled along a latitudinal gradient. Significant correlations are indicated with an asterisks (*P < 0.05, after Hommel multiple testing correction). Inversions harboring ORs are indicated in green.

Inversion regions in the spruce bark beetle did not structure in the same way as the collinear part of the genome and most regions do not show any clustering into a southern and northern group (supplementary fig. S2, Supplementary Material online). Frequencies of two inversions were significantly correlated with latitude (Fig. 3; see further discussed below). Almost all identified inversions were polymorphic across the beetle's European range, except for one inversion (Inv9) that was polymorphic only in northern populations. For three inversions (Inv2, Inv5, and Inv16.2 + Inv23.2), unambiguous genotyping was only possible across part of the species range (see above). The differentiation between haplotypes of some inversions was moderate or high (mean *F*_ST_ range 0.15 to 0.64; Fig. 2; supplementary fig. S2, Supplementary Material online), and, according to our age estimates, the inversions originated before the Last Glacial Period. Many inversions may be several million years old and probably predate the within-species differentiation into a southern and northern group. This was not unexpected, as many known inversion polymorphisms have been segregating within species for hundreds of thousands or millions of years (see table in Wellenreuther and Bernatchez 2018), sometimes even persisting through multiple speciation events (Brelsford et al. 2020).

### Association between Inversions and Fitness-Related Traits

Inversion polymorphism is often associated with the maintenance of complex polymorphic phenotypes (Schwander et al. 2014; Lamichhaney et al. 2015). Although the spruce bark beetle does not exhibit easily identifiable phenotypes, such as distinct color patterns or mating strategies, we were able to test for associations between inversions and two complex traits of key importance for many insects: diapause and olfaction.

Diapause is an effective strategy to increase fitness and avoid mortality by synchronizing seasonal occurrence with critical resources and mitigating harmful effects of adverse abiotic conditions (Denlinger 2022). Diapause is characterized by suppressed development, metabolism, and reproduction, accompanied by increased stress resistance (Denlinger 2022; Schebeck et al. 2024). While multiple environmental factors can influence this complex process, many aspects of diapause, such as its induction and termination (i.e. the decision to enter into or exit from diapause, respectively), are heritable (Roff 1996). These traits may be controlled by a small number of loci or be highly polygenic (Paolucci et al. 2016; Koštál et al. 2017; Pruisscher et al. 2018; Kozak et al. 2019; Dowle et al. 2020; Denlinger 2022). The spruce bark beetle enters reproductive diapause as an adult to respond to unfavorable winter conditions (Schebeck et al. 2017, 2024). This diapause is physiologically and behaviorally characterized by suppressed development and reproduction, a lack of flight activity, an increased resistance against cold temperatures, and movement to suitable overwintering habitats (Schopf 1985; Annila 1969; Schopf 1989; Doležal and Sehnal 2007; Dworschak et al. 2014; Schebeck et al. 2017). The spruce bark beetle exhibits two diapause phenotypes that could be associated with polymorphic inversions: a facultative photoperiod-regulated diapause and an obligate photoperiod-independent diapause (Schebeck et al. 2022). Beetles enter facultative diapause when day-length drops below specific threshold values that vary along latitudinal gradients. When day length is above the threshold, beetles develop and reproduce normally (Doležal and Sehnal 2007; Schroeder and Dalin 2017; Schebeck et al. 2022). Obligate diapausing beetles, however, enter diapause in each generation as a regular part of their life cycle, independently of day-length cues. These beetles can therefore produce only one generation per year and do not resume development and reproduction until the next year (Schebeck et al. 2022). Both diapause phenotypes are usually found in European populations, with facultative diapause prevailing in more southern latitudes and obligate diapause being most common in northern regions (Schebeck et al. 2022). We found no association between diapause phenotypes and inversion genotypes (exact *G*-test; supplementary table S3 and fig. S10, Supplementary Material online), nor did we find any genomic regions that were highly differentiated between facultatively and obligately diapausing individuals (supplementary fig. S11, Supplementary Material online). However, it should be noted that due to a small sample size, we were only able to test for complete association between phenotype and inversion genotype.

Olfaction is another fitness-related trait in many insects, including bark beetles (Raffa et al. 2016). Insect odorant receptors (ORs) are encoded by a large and dynamically evolving gene family. Some ORs are evolutionarily conserved across species within insect orders, whereas many others are species or genus specific. For the spruce bark beetle, odorant detection is essential for host and mate finding, as well as for recognition and maintenance of symbiotic fungal associations (Andersson et al. 2009; Kandasamy et al. 2019). We examined 73 antennally expressed ORs (Yuvaraj et al. 2021) and found that 46% of these (33 of 71 ORs, which mapped to 26 IpsContigs) were located within inverted regions (Table 1). A permutation test (10,000 iterations; randomizing inversion locations across 170 Mb) validated the overrepresentation of OR genes within inversions (*P* = 0.0325; i.e. in just 325 of the 10,000 permutations, the number of ORs located within inversions was higher than or equal to the number observed in the real data). In addition, several Ips-specific ORs (five out of seven ORs from an Ips-specific OR clade; Hou et al. 2021) were located within inverted regions; specifically, four out of these five were predominantly on IpsContig13. The five Ips-specific ORs (ItypOR23, ItypOR27, ItypOR28, ItypOR29, and ItypOR49) have been functionally characterized and respond to compounds produced primarily by beetles (pheromones), the host tree, or fungal symbionts (Hou et al. 2021; Powell et al. 2021). We found no difference in OR number between inversion haplotypes (i.e. there were no OR deletions). Most ORs located in inversions harbor multiple nonsynonymous variants segregating within the studied spruce bark beetle populations (supplementary table S4, Supplementary Material online) and nine ORs (supplementary table S5, Supplementary Material online) had at least one fixed or nearly fixed (*d*_xy_ > 0.9) nonsynonymous variant between inversion haplotypes. These diverged ORs were located at Inv5 (ItypOR43JOI and ItypOR29), Inv13 (ItypOR23 and ItypOR27), Inv14.1 (ItypOR20NTE and ItypOR36), and Inv16.1 (ItypOR30, ItypOR31, and ItypOR16). Among these ORs only ItypOR30 had dN/dS > 1 (i.e. signature of positive selection; two nonsynonymous substitutions, McDonald–Kreitman test, *P* = 0.55).

Olfactory receptor activity was one of the enriched gene ontology (GO) categories found in inversions showing significant latitudinal variation (Fig. 3; supplementary table S6, Supplementary Material online; 0.01 < *P* < 0.05) and one of these inversions harbored multiple OR genes (Fig. 3; Inv13, Table 1). In *Drosophila pseudoobscura*, ORs are also associated with inversions (Fuller et al. 2016, 2017). Interestingly, Inv13 includes a gene-encoding ItypOR23, a receptor that has been previously shown to primarily respond to fungal volatiles (Hou et al. 2021). This OR was also one of the most divergent ORs between alternative inversion haplotypes (supplementary table S5, Supplementary Material online). We hypothesize that different OR alleles associated with Inv13 may be involved in spruce bark beetle interactions with fungal associates present across Europe. As many other bark beetle species, the spruce bark beetle is associated with several spatially differentiated fungal symbionts that can help beetles to exhaust and overcome tree defenses (Zhao et al. 2019). Interestingly, recent studies suggest that beetles can recognize volatile compounds emitted by certain beneficial fungi and use these volatiles to locate and pick up the fungi. Furthermore, individual beetles may also have preferences for different fungal species (Kandasamy et al. 2019; Zhao et al. 2019; Kandasamy et al. 2023). Spruce bark beetles in Germany are, for example, more attracted to fungal species that are common in Germany (such as *Grosmannia penicillata* and *Endoconidiophora polonica*) than to rarer species (*Leptographium europhioides*). Unpublished data also suggest that Swedish beetles are more attracted to *L. europhioides*, which is common in Sweden (personal communication, D. Kandasamy). Although preliminary, these observations suggest that beetle preferences may be tuned to the local fungal community, and we speculate that inversions may be involved in the recognition of region-specific fungal species.

Diapause and olfaction are not the only polymorphic complex traits in *I. typographus*. Several other potentially interesting traits include the existence of pioneer individuals that are the first to infest host trees and re-emergence of some females after egg laying to establish so-called sister broods in new trees (Anderbrant and Lofqvist 1988; Anderbrant 1989; Lehmanski et al. 2023). Other less obvious/visible phenotypes could be tested for their association with inversion polymorphisms. GO enrichment analysis provided a list of gene categories of interest (supplementary table S7, Supplementary Material online), but comprehensive research is needed to understand the relationship between spruce bark beetle phenotypes and the species' complex inversion polymorphism landscape.

### Evolutionary Mechanisms Maintaining Inversion Polymorphism in the Spruce Bark Beetle

**Fig. 4.**
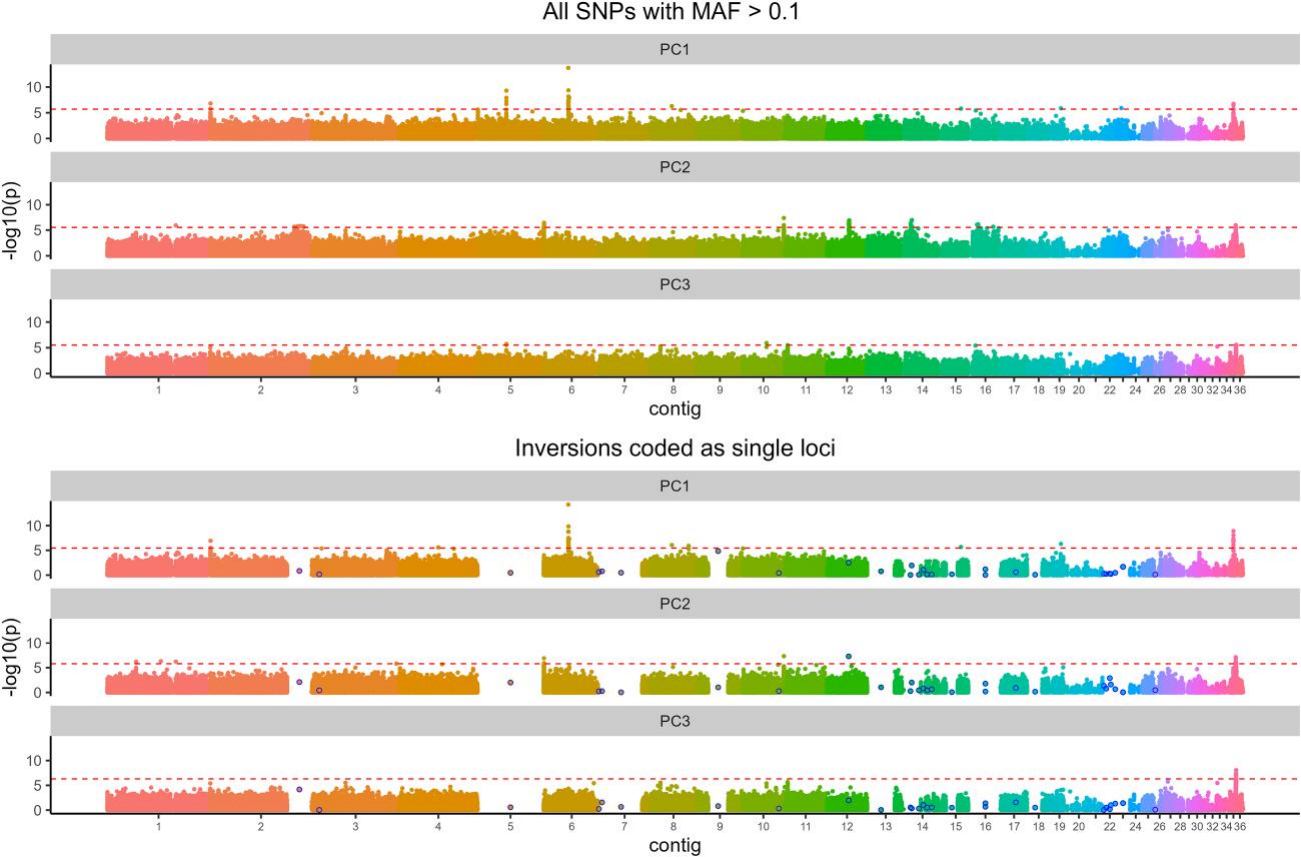
GEAs across 36 contigs in the *I. typographus* genome. The *y* axis shows log-transformed *P*-values from LFMM analysis testing association between SNPs and three principal components (PC1 to 3, facet labels) summarizing the environmental variation between beetle populations. Each dot represents a single SNP and different colors different contigs. The upper three panels show results for all SNPs and the lower three panels show results using data where each inversion (blue circles) was coded as a single locus.

Several nonmutually exclusive evolutionary mechanisms can maintain polymorphic inversions within species, the most important mechanisms being divergent and balancing selection (Wellenreuther and Bernatchez 2018; Faria, Johannesson, et al. 2019). The importance of divergent selection has been postulated based on allele frequency patterns and associations of polymorphic inversions with local adaptations that persist despite extensive intraspecific gene flow (Tigano and Friesen 2016). For example, a recent study of deer mice (*Peromyscus maniculatus*; Harringmeyer and Hoekstra 2022) identified multiple polymorphic inversions with clinal variation across environmental gradients in two distinct habitats. Such frequency changes have also been reported across hybrid zones (Faria, Chaube, et al. 2019) or latitudinal gradients (Mérot et al. 2021) in other species. Although the spruce bark beetle does not occupy distinct environmental niches, it inhabits forests across a very wide latitudinal gradient (spanning at least 16°). It is also a species with high dispersal capacity and extensive gene flow, as indicated by low *F*_ST_ across its range. We found a significant correlation between the frequency of inversion haplotypes in populations and geographic location (latitude) for two inversions (Fig. 3; Inv12 and Inv13; *r*^2^ 0.68 and 0.62, respectively). Importantly, in both cases, *r*^2^ was greater than what we observed for random SNPs with similar frequencies but situated outside inversions (*P* = 0.003 and 0.021 for Inv12 and Inv13, respectively). For multiple SNPs (59), climate and land cover variation (summarized using PCA) were associated with allele frequency changes; however, no such association was found for inversion genotypes (Fig. 4). The identified SNPs were within 14 genes, five of which had assigned GO categories (supplementary table S8, Supplementary Material online). All the above results indicate that there may be selection across environmental gradients in the spruce bark beetle but that this is not the only, or even a major, force maintaining inversion polymorphism within the species. It also remains an open question whether some inversions are involved in local adaptation or not.

Although “local adaptation” is a major hypothesis proposed to explain inversion polymorphism (Christmas et al. 2019; Akopyan et al. 2022; Harringmeyer and Hoekstra 2022), balancing selection and related mechanisms may also be important in maintaining polymorphic arrangements (Faria, Johannesson, et al. 2019; Mérot et al. 2020; Berdan et al. 2021). Such mechanisms include overdominance, associative overdominance, frequency-dependent selection, and selection that varies spatially and/or temporally. We found no support for overdominance playing a role in the spruce bark beetle, as we detected no excess of inversion heterozygotes compared to Hardy–Weinberg (HW) expectations in any of the populations. The only significant deviations from HW expectations were found in Inv12 (a deficit of heterozygotes across the whole species range) and Inv22.4 (more heterozygotes than expected across the whole species range and southern, Polish, and northern populations; supplementary table S9, Supplementary Material online). We therefore looked more closely at mutation load, which, if high, can indicate the importance of associative overdominance in maintaining inversion polymorphism. Theory predicts that recessive deleterious mutations will accumulate on both inversion arrangements but that most of these mutations will be private to only one arrangement (Navarro et al. 2000; Faria, Johannesson, et al. 2019; Berdan et al. 2021). This would lead to associative overdominance, as in heterozygotes, the effects of deleterious recessive alleles on one arrangement would be masked by the wild-type alleles on the other arrangement. The result would be long-term maintenance of the inversion polymorphism, resulting in strong divergence between inversion haplotypes (Navarro et al. 2000; Guerrero et al. 2012; Berdan et al. 2021).

Interestingly, several stable evolutionary scenarios that maintain polymorphic inversions are possible (for details, see figure 4 in Berdan et al. 2021). These scenarios differ in the expected mutation load, fitness, and frequency of the corresponding genotypes. Given the haplotype frequencies we observed in spruce bark beetle inversions, two scenarios are likely. First, that minority arrangements experience higher mutation load (due to reduced recombination and lower population size) but are maintained in the population at low frequencies due to, e.g. associative overdominance. Such a mechanism would favor balanced inversion polymorphisms of intermediate to large sizes (harboring hundreds of genes; Ohta 1971; Connallon and Olito 2022) and has been shown to play a role in maintaining polymorphic inversions in several insect species (Yang et al. 2002; Jay et al. 2021). Second, mutation load may accumulate on one or both inversion arrangements but be mitigated by the haplotype structuring process, i.e. the existence of multiple diverged subhaplotypes among inversion homozygotes that reduces the mutation load within homozygotes. If this process operates within one or both inversion arrangements, it may result in more equal frequencies of alternative inversion haplotypes. Such a mechanism can operate when genetic variation and mutation load are high and are theoretically possible in the spruce bark beetle system (Berdan et al. 2021).

**Fig. 5.**
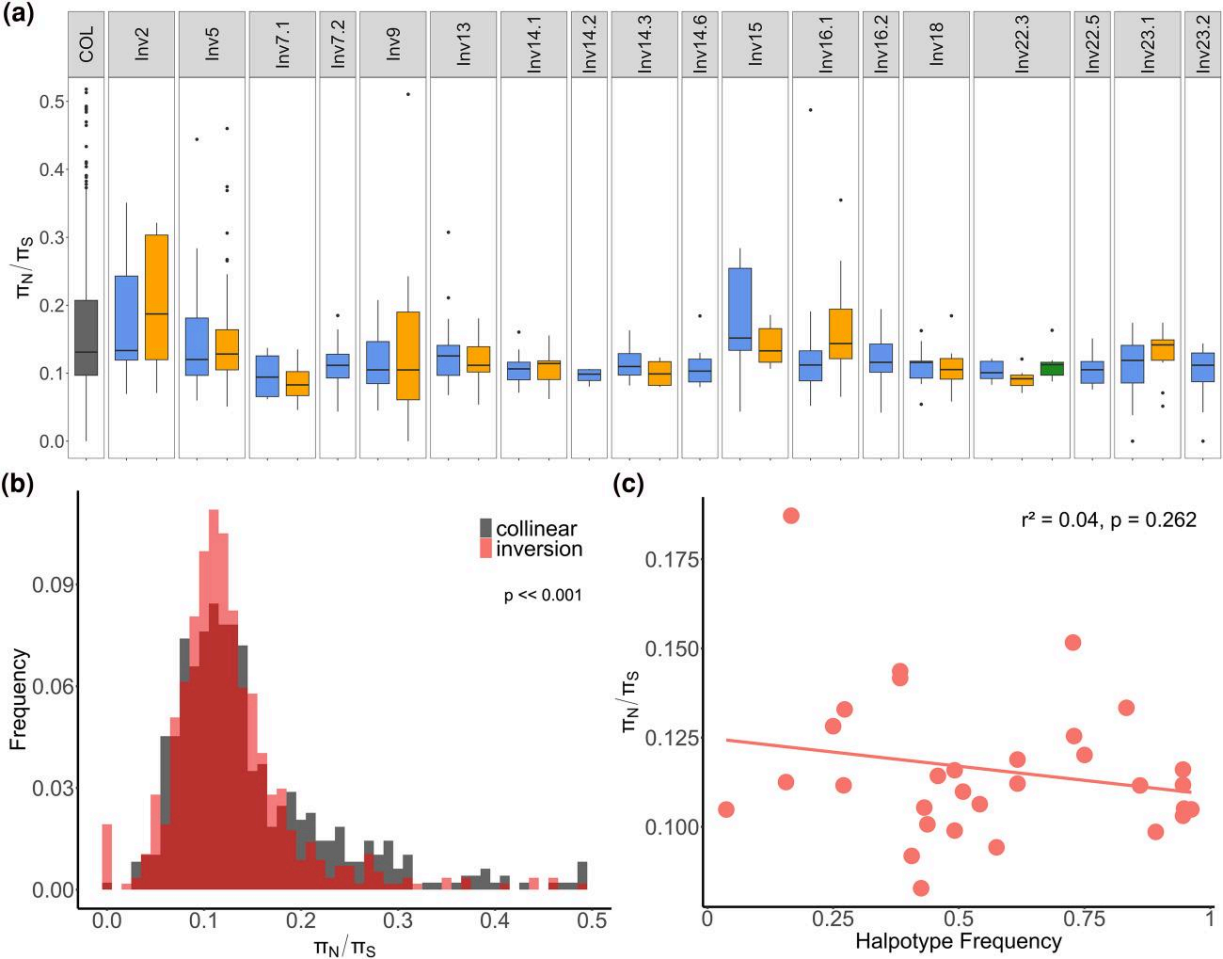
Mutation load analysis in *I. typographus*. a) The ratio of nonsynonymous to synonymous nucleotide diversity (*π_N_*/*π_S_*) computed for 200-kb windows along collinear parts of the genome (COL) and different inversion haplotypes (blue: major haplotype; yellow: minor haplotype; green: haplotype produced by double-crossover events between minor and major haplotypes). *n* = 4 to 55, 200-kb windows per inversion haplotype and *n* = 531 for COL. For clarity, *π_N_*/*π_S_* outliers above 0.5 are not shown (a total of 58 values, 45 of them in COL; supplementary fig. S16, Supplementary Material online shows the plot with these outliers). The lower and upper box hinges correspond to the first and third quartiles, whiskers show 1.5× the interquartile range. b) Distribution of *π_N_*/*π_S_* values per 200-kb window in inversions and collinear part of the genome (nonparametric two-sided Wilcoxon test). c) Correlation between median *π_N_*/*π_S_* for inversion haplotypes and their frequency across 18 spruce bark beetle populations. Only inversion haplotypes that were present in more than four individuals and that included four or more 200-kb windows, and more than five genes were included in the mutation load analyses.

Contrary to these theoretical expectations, we observed no sign of increased mutation load (measured by the *π_N_*/*π_S_* ratio) in inversion regions compared to the collinear part of the spruce bark beetle genome (supplementary table S10, Supplementary Material online; Fig. 5). We also found no sign of haplotype structuring that could reduce the mutation load within inversion homozygotes (supplementary fig. S12, Supplementary Material online). The only significant within-homozygote clustering we observed was in a few inversion haplotypes. However, this clustering divided individuals into southern and northern clades. This suggested either neutral differentiation or divergent selection acting on one of the inversion arrangements, rather than haplotype structuring being associated with mutation load (supplementary fig. S12, Supplementary Material online). These results are consistent with observations in other species of no significant mutation load when inversion haplotypes are subjected to divergent selection resulting in geographic structuring (Harringmeyer and Hoekstra 2022; Huang et al. 2022). In such cases, inversions facilitate adaptive divergence but do not tend to accumulate a mutation load. However, the geographic structuring of inversion polymorphisms is weak in the spruce bark beetle, and many inversions appear to have a slightly lower mutation load associated with the more common inversion haplotype (Fig. 5; supplementary table S11, Supplementary Material online). It is possible that the accumulation of a mutation load in the spruce bark beetle is mitigated by a high effective population size in this species, as many homozygous individuals in populations may purge mutation load to some extent. It is also possible that the *π_N_*/*π_S_* ratio does not fully capture the patterns of mutation load in the species and that further and more in-depth analyses are needed.

Overall, the absence of heterozygote excess does not support a role of overdominance in the maintenance of inversion polymorphism in the spruce bark beetle. Likewise, the absence of an elevated mutation load in inverted regions does not support a role of associative overdominance either. However, since we only have genomic data from a single season, we cannot rule out that other mechanisms, such as negative frequency-dependent selection or antagonistic pleiotropy, could maintain balanced inversion polymorphisms. Additional temporal data spanning multiple years (generations) are needed to test whether temporally varying selection affects the frequencies of inversion haplotypes in the spruce bark beetle. Further research is thus essential to determine the importance of different mechanisms in maintaining inversion polymorphisms within this species.

### Far-Reaching Consequences of Having an Inversion-Rich Genome

The presence of multiple polymorphic inversions can have significant consequences both for the evolution of a species and for evolutionary inferences based on genome-wide polymorphism data. Importantly, polymorphic inversions are a reservoir of genetic variation that can facilitate adaptation to rapidly changing environments. Indeed, several studies have shown that polymorphic inversions support rapid adaptation to changing climatic conditions (Rane et al. 2015; Kapun and Flatt 2019; McCulloch and Waters 2023) or adaptive tracking of fluctuating environments (Nunez et al. 2023). Spruce bark beetle populations are subjected to seasonal weather changes and a rapidly changing environment due to strong anthropogenic pressures (Hofmann et al. 2024). Warmer weather and drought periods have been associated with an intensification of bark beetle outbreaks (Biedermann et al. 2019; Bentz et al. 2021; Hlásny et al. 2021), which may act as a strong selection factor within bark beetle populations. Whether inversions are involved in rapid adaptations in the spruce bark beetle is an open question that requires further investigation.

Abundant polymorphic inversions within the genome can potentially bias inferences about, e.g. a species' demographic history and selection, if it is not properly accounted for. This is mainly due to the suppression of recombination within inversion regions and selection that influence inversion frequencies and variation patterns. It is well known that nonequilibrium demography and selection can leave similar genomic signatures. Traditionally, demographic analyses have used noncoding parts of the genome, based on the assumption that directional selection mostly affects protein-coding regions. However, there is growing evidence for the importance of background selection in shaping genome-wide diversity, and this is moving the field toward incorporating linked selection into inferences of demographic history (Li et al. 2012; Johri et al. 2021). We believe that new analytical approaches should also consider the potential influence of polymorphic inversion landscapes, because variation patterns of inverted regions can be shaped by different types of balancing selection. In addition, genomic scans for selection in inversion-rich genomes may yield biased results because recombination is reduced within inversion regions. Importantly, the effect of reduced recombination may extend outside inversions (Adrion et al. 2020; Koury 2023; Li et al. 2023). Quantifying how inversions and inversion-rich genomes influence various evolutionary inference methods may become a standard approach to test the robustness of these methods (Novo et al. 2023).

## Conclusions

Facilitated by advanced sequencing technologies and a recent focus on structural variation, evolutionary biology is facing a new era of exciting discoveries that will deepen our understanding of genome complexity, genome-wide patterns of variation, and the major evolutionary forces responsible for shaping these patterns (Lynch and Conery 2003; Wellenreuther et al. 2019; Weissensteiner et al. 2020; Sirén et al. 2021; Berdan et al. 2023; Wold et al. 2023). While extensive genome rearrangements are common among species across the tree of life (Bhutkar et al. 2008; Escudero et al. 2023; Höök et al. 2023), and perhaps more so than previously thought (Lewin et al. 2024), accumulating genomic data suggests that such rearrangements are also common at the intraspecific level (Faria, Chaube, et al. 2019; Faria, Johannesson, et al. 2019; Todesco et al. 2020; Harringmeyer and Hoekstra 2022; Porubsky et al. 2022; Reeve et al. 2023). Here, we characterized one of the most complex polymorphic inversion landscapes described to date and found no support for any of several mechanisms that often are associated with the presence of polymorphic inversions (including directional selection, overdominance and associative overdominance, and strong divergent selection). These results suggest that inversions are either neutral or maintained by the combined action of multiple evolutionary forces and influence evolutionary processes in complex ways. Complete neutrality of large inversions is unlikely (Connallon and Olito 2022), especially in species with large Ne, such as the spruce bark beetle, in which selection should be particularly efficient. Several studies have convincingly linked polymorphic inversions to adaptation (Lowry and Willis 2010; Twyford and Friedman 2015; Kirubakaran et al. 2016), but as more species are systematically screened for structural variation, data are accumulating on species in which (at least some) SVs show no clear adaptive advantage or disadvantage (this study and Harringmeyer and Hoekstra 2022). These findings raise questions about how prevalent inversions and other SVs are at different levels of the tree of life and, importantly, about their consequences for individual fitness and the role they play in genome evolution and reorientation of the evolutionary process (Marroni et al. 2014; Berden et al. 2023).

## Materials and Methods

### The Spruce Bark Beetle Genome and Its Quality

The spruce bark beetle karyotype is *n* = 14 + XYp. The species genome assembly consists of 272 contigs, and the 36 largest contigs (>1 Mb) constitute the most of the assembly (78% of the 236.8-Mb-long assembly, N50 = 6.65 Mb; Powell et al. 2021). Telomeric motifs (sequences present at the end of chromosomes) were identified at the end of eight contigs (including the five largest contigs). BUSCO analysis indicated that 99.5% of the genes present in the insect database (insects_odb9) were also present among the predicted spruce bark beetle genes. To further investigate the quality of the spruce bark beetle assembly, we compared it to the chromosome-level assembly of the reference genome of the congener *I. nitidus* (NCBI: GCA_018691245.2; 1.5 to 2.3-Mya divergence from the spruce bark beetle; Wang et al. 2023). For clarity, we only extracted the 36 and 16 longest contigs/pseudochromosomes from *I. typographus* and *I. nitidus* genomes, respectively, and used these in our synteny analysis. Syntenic blocks were detected using ntSynt version 1.0.1 (Coombe et al. 2024), with divergence set to 2.5% and using default values of the remaining parameters. Links longer than 10 kb were processed using scripts provided with ntSynt and visualized using gggenomes version 1.0.0 (Hackl et al. 2024; https://github.com/thackl/gggenomes) in R. SeqKit version 2.6.1 (Shen et al. 2024) was used for operations on fasta files, such as extraction or reverse complement of sequences.

### Sampling of Beetles

Adult spruce bark beetles were collected with pheromone-baited traps in the spring and summer of 2020. In total, we sampled 18 populations throughout Europe, using 13 to 14 individuals per locality (253 individuals in total) (Fig. 2; supplementary table S5, Supplementary Material online). Throughout the text, we use the term “population” to refer to an individual site or a collection of closely situated sites (within about 50 km). In Austria, we pooled individuals from three localities that were up to 120 km apart because of small sample sizes (five beetles or less per site). Populations from Fennoscandia are referred to as northern populations (or the northern group) and populations from central Europe are referred to as southern populations (or the southern group). Polish populations are considered separately from other central European populations because downstream analysis revealed high admixture proportions from the northern group. Beetles were brought alive to the laboratory, starved on a paper diet for several days, dissected, sexed based on genitalia morphology, and subjected to DNA extraction (described below).

### DNA Extraction and Genome Resequencing

DNA was extracted from the whole body of dissected beetles using the Wizard Genomic DNA Purification Kit (Promega). The concentration of extracted DNA was estimated using a Qubit fluorometer (Thermo Fisher Scientific). Genomic libraries were prepared with NEBNext Ultra II FS DNA Library Prep with Beads (New England Biolabs), with single indexes. Individual libraries were combined into three pools and 2 × 150 bp paired-end sequenced in three lanes of a S4 flowcell using the NovaSeq 6000 instrument and v1 sequencing chemistry (Illumina Inc.). Sequencing was done by the National Genomics Infrastructure, SNP&SEQ Technology Platform (Uppsala, Sweden). To assess the overall genotyping error, we prepared and sequenced duplicate libraries for nine individuals.

### Data Preparation and Filtering

Details of raw data processing and filtering are described in the Supplementary material. In short, raw reads were mapped to the reference genome (Powell et al. 2021) using Bowtie 2 version 2.4.2 (Langmead and Salzberg 2012). Duplicated reads were removed using Picard MarkDuplicates version 2.24.1 (https://broadinstitute.github.io/picard/). To detect and correct systematic errors in base quality scores, recalibration was done using the GATK version 4.1.9.0, BaseRecalibrator, and ApplyBQSR (McKenna et al. 2010; Depristo et al. 2011). Variant calling and genotyping were done using GATK HaplotypeCaller, CombineGVCFs, and GenotypeGVCFs. GATK VariantRecalibrator and ApplyVQSR were used to calculate and filter (by variant) quality score log-odds (VQSLOD). Bcftools version 1.11 (Danecek et al. 2021) was used to remove insertions and deletions (indels) as well as any polymorphisms of five bases upstream and downstream. GATK VariantFiltration was applied to mask all genotypes with low sequencing depth or low genotype quality (McKenna et al. 2010; Depristo et al. 2011). Variants that were not biallelic SNPs or did not meet the recommended hard filtering thresholds (GATK Team; see Supplementary material) were filtered out. To filter out polymorphisms that could come from duplicated regions, we removed variants located within repeat-masked regions of the genome (Powell et al. 2021), variants with excessive overall coverage (mean + 1 SD), and variants with heterozygote excess. Variants for which genotypes could be detected in less than half of the individuals were also removed. We used PLINK version 1.90b6.18 (Purcell et al. 2007) to detect sample contaminations, swaps and duplications, and familial relationships (e.g. sibling pairs present in the data), which might bias downstream analyses. Individuals with excessive coverage were removed, as these could be a result of human errors during library preparation or pooling. We only analyzed contigs longer than 1 Mb in downstream analysis. These constituted 78% of the genome assembly, i.e. a total of 186 Mb. Since part of IpsContig33 had high similarity to mtDNA, this contig was not included in the downstream analysis. Genotyping error was assessed using GATK Genotype Concordance.

### Genome-Wide Genetic Variation and Its Geographic Structuring

Genome-wide genetic structuring was explored by PCA using PLINK. The most likely number of genetic clusters and admixture proportions was estimated using NGSadmix (Skotte et al. 2013). The analysis was run for five different *K*-values (1 to 5; ten replicates per *K*-value), using a minor allele frequency (MAF) filter of 0.05 and 10,000 iterations, and a SNP data set that was pruned for LD using PLINK (–indep-pairwise 50 10 0.1 option). To choose the most likely number of genetic clusters, the results were examined using CLUMPAK (http://clumpak.tau.ac.il/index.html). To examine the influence of inversions on genetic clustering and to facilitate inversion genotyping, NGSadmix was run separately for (i) all autosomal contigs without potential inversions and (ii) each potential inversion.

To assess genetic differentiation among different spruce bark beetle populations Weir and Cockerham (1984)  *F*_ST_ was estimated using VCFtools version 0.1.16 (Danecek et al. 2011). *F*_ST_ was calculated between population groups identified by NGSadmix, as well as among all population pairs. Additionally, we summarized *F*_ST_ values in 100-kb overlapping windows (using 20-kb steps) along the contigs. Window-based analyses were done for all contigs and all possible population pairs. In addition, absolute sequence divergence (*d*_xy_), nucleotide diversity, and Tajima's *D* statistic were estimated and summarized for 100-kb nonoverlapping windows using ANGSD version 0.935-44-g02a07fc (Korneliussen et al. 2014). These three statistics were calculated for each population separately and were based on a maximum likelihood estimate of the folded site frequency spectrum. We excluded sites with mapping quality below 30 phred, base quality score below 20, and coverage less than three times the population sample size and more than three times the average coverage, following the approach used in Delmore et al. (2018). The ANGSD calculations were based on allele frequencies estimated from genotype likelihoods (Li 2011) and ngsPopGen scripts (https://github.com/mfumagalli/ngsPopGen).

### Identification of Inversions, Their Geographic Distribution, and Variation Patterns

To identify inversions, we followed the standard population genetic approach (see e.g. Huang et al. 2020; Mérot et al. 2021; Reeve et al. 2023). Potential chromosomal inversion regions were identified based on (i) per contig PCAs, (ii) local PCAs, (iii) patterns of heterozygosity, and (iv) LD clustering. Per contig PCAs were performed as a first screen for inversions using PLINK (Purcell et al. 2007). Local PCAs were performed to identify the approximate position of inversions using the *lostruct* R package (Li and Ralph 2019). Analysis was performed using two independent approaches: localPCA analysis using all analyzed contigs and localPCA analysis for each contig independently. The first analysis was run following the approach described in Huang et al. (2020). Windows were considered outliers if their multidimensional scaling (MDS) scores were more than 2 SD greater than the mean across all windows. The maximum number of windows between what was considered separate inversions was set to 20. In the second analysis, separate contigs were subjected to the *k*-means clustering algorithm to define groups of windows that formed a single inversion. *k*-means clustering was performed on the first MDS scores. Different *k*-values (number of window clusters) were tested for each contig so that structurally different parts of the contig were isolated into separate clusters. For both approaches, different window sizes (1, 10, and 100 kb) were tested.

LD among SNPs (thinned by selecting one SNP every 10 kb; MAF > 5%) was calculated for each contig using PLINK. We considered a genomic region to be an inversion region if (i) local PCA analysis identified the region as an outlier, (ii) the region exhibited high LD (most SNPs having *r*^2^ > 0.4), and (iii) PCA performed on SNPs from this region separated individuals into three distinct groups with heterozygosity patterns matching the expectation of the middle group of the PCA presenting higher heterozygosity.

Genotyping of individual beetles with respect to the inversion haplotypes they carried was done based on NGSadmix clustering (with *k* = 2 inversion heterozygotes having mixed ancestry in approximate 50/50 proportions; supplementary fig. S13, Supplementary Material online). In a few more complex cases (putative overlapping inversions and double-crossover events) genotyping was done by clustering individuals based on the PCA groups visible along PC1 and PC2 (for more details, see supplementary fig. S3, Supplementary Material online and related text). Contigs with <10,000 SNPs provided ambiguous results and were excluded from genotyping. Approximate inversion boundaries were defined based on local PCAs and detection of sharp borders in LD clusters. The population genetic approach used in this study is a standard approach to detect putative large polymorphic inversions but is not able to provide details on inversion breakpoints.

As further evidence for inversions, we estimated population recombination rates (rho) for each putative inversion region using individuals with a particular inversion genotype (separately for homozygous and heterozygous individuals). We followed the approach used in Jones et al. (2019) and sampled 20 individuals from each genotype group. For genotype groups with less than ten individuals, rho was not calculated, but rho was calculated for groups with fewer than 20 but more than ten individuals. Watterson theta estimates were used to create a custom likelihood lookup table using the Ldhat 2.2 program *complete* (McVean et al. 2002). The *interval* program was used to estimate the population recombination rate across investigated inversion (and adjacent regions in the same contig). The *interval* algorithm was run for 2 million iterations and the chain was sampled every 10,000 iterations with a burn-in of 100,000 generations (using the Ldhat package's *stat* program, block penalty = 5). Population recombination rates were summarized in nonoverlapping 100-kb windows using a custom perl script.

Inversion genotype and haplotype frequencies were calculated using an in-house R-script. Frequencies were calculated (i) within each population, (ii) within the southern and northern groups, and (ii) for all sampled populations combined. Deviations from HW equilibrium were estimated for all three data sets. Inversions with only two haplotypes were tested for HW deviations using Fisher's exact tests. Inversions with more than two haplotypes (including recombinant haplotypes between two inversion arrangements) were tested using a permutation test, and sex chromosome inversions were tested as described in Graffelman and Weir (2018). All tests were done using the R package *HardyWeinberg* (Graffelman 2015). To investigate if inversion haplotypes differed in frequency along environmental gradients, Pearson correlation between inversion haplotype frequencies and latitude/longitude was calculated. To assess whether correlations were stronger than expected based on the collinear part of the genome, we performed a permutation test. We generated sets of 1,000 randomly selected SNPs with MAFs similar to (±0.05) the frequencies of inversions showing significant correlations. We first calculated the MAF of each SNP for each population and then calculated Pearson's correlation between SNP frequencies and population latitudinal coordinates. The resulting *r*^2^ distribution was used to determine the number of loci outside inversions that exceeded the *r*^2^ obtained from the correlation between inversion haplotype frequency and latitude and to calculate the *P*-value of the test. To assess levels of genetic differentiation between inversion haplotypes, *F*_ST_ and *d*_xy_ between alternative inversion haplotypes (AA and BB) were estimated following the approach described above. Deletions present in alternative inversion haplotypes were not included in *d*_xy_ calculations but were summarized separately using custom scripts.

### Testing for Enrichment of GO Terms in Inversions

We used gene annotations from Powell et al. (2021) and tested for overrepresentation of GO terms among genes located within inversions using R package topGO (Alexa and Rahnenführer 2024), applying Fisher's exact test and the “weight01” algorithm (Alexa et al. 2006) to deal with the GO graph structure; only GO categories with at least ten members among SNP-associated genes were considered.

### Inversion Age Estimation

Absolute sequence divergence between alternative inversion haplotypes was used to calculate the approximate time of divergence of inverted and noninverted haplotypes. We used the equation *T* = *d*_xy_/2*μ*, where *T* is the divergence time in generations, *μ* is the mutation rate per site per generation, and *d*_xy_ is a mean *d*_xy_ calculated based on per SNP values estimated in ANGSD. Since mutation rates of the spruce bark beetle are unknown, we used a range of mutation rate estimates available for three diploid, sexually reproducing insects: *Drosophila melanogaster* (Krasovec 2021), *Heliconius melpomene* (Keightley et al. 2015), and *Chironomus riparius* (Oppold and Pfenninger 2017). These per-generation and per-basepair mutation rate estimates varied from 2.1 to 11.7 × 10^−9^. This approach could only give rough inversion age estimates due to the uncertainty of the mutation rate estimates, probable intraspecific variation in mutation rate (Krasovec 2021), and a (likely) substantial influence of selection and gene flux (Charlesworth 2023).

### Mutation Load Estimation

To estimate mutation load, we calculated the ratio of nucleotide diversity at nonsynonymous sites (*π_N_*) versus synonymous sites (*π_S_*). Mutation load (*π_N_*/*π_S_*) was calculated separately for each inversion homozygote and for the collinear part of the genome (for all individuals). We computed nucleotide diversity for each site using SNPGenie (Nelson et al. 2015). The *π_N_*/*π_S_* ratio was estimated in 200-kb windows using an in-house R script. To account for the fact that inversions can greatly suppress recombination in surrounding parts of the genome (Koury 2023), the collinear part of the genome was divided into two groups: (i) a group including all collinear 200-kb windows outside inversions and (ii) a group including all collinear windows outside inversions but excluding windows that came from contigs with inversions (so-called strict filtering). Both collinear data sets were used to test for overall differences in mutation load between inversions and the collinear part of the genome (using two-sided *t*-test). One-sided *t*-tests were used to test whether minor (less frequent) homokaryotypes had higher mutation loads than major (more frequent) homokaryotypes. Homokaryotypes that contained fewer than four 200-kb windows and were present in few individuals (two thresholds were tested: <4 and <10 individuals) were excluded from the analysis. Additionally, windows with a small number of genes were excluded (two thresholds were tested: <5 and <10 genes).

Haplotype structuring, i.e. the presence of two or more distinct subhaplotypes among inversion homozygote haplotypes, can prevent fitness degeneration on one or both inversion haplotypes by carrying partially complementary sets of deleterious recessive alleles (Charlesworth and Charlesworth 1997; Berdan et al. 2021). To check if any inversion homozygotes exhibited haplotype structuring, we first phased the data using Beagle 5.2 (using default settings; Browning and Browning 2007). Next, for each inverted region and homokaryotype, we filtered out all variants with MAF < 0.1 and used PGDSpider 2.1.1.0 (Lischer and Excoffier 2012) to convert VCFs to full-length sequences. Finally, we constructed neighbor-joining trees for alleles within haplotypes using MEGA7 and investigated if topologies showed the presence of distinct clusters within homokaryotype groups (Kumar et al. 2016).

### Phenotype–Genotype Associations

To test whether inversion polymorphisms were associated with diapause phenotypes, we resequenced the whole genome of 18 female spruce bark beetle individuals with defined individual diapause phenotypes (ten beetles expressing facultative diapause and eight beetles expressing obligate diapause). These individuals came from a spruce bark beetle diapause study by Schebeck et al. (2022). In brief, diapause phenotypes were determined under controlled conditions by exposing beetles from three locations (northern Scandinavia, Central Europe low-elevation site, and Central Europe high-elevation site) to diapause-inducing or diapause-averting photoperiodic conditions. Diapause expression was assessed by studying gonad development and timing of emergence from experimental logs (two reliable indicators of diapause expression in the spruce bark beetle). The individuals with the best DNA extractions were selected for sequencing using the DNBseq platform (BGI Tech Solutions, Poland) to a mean coverage of 20×. Data were processed in the same way as described above (raw data processing and inversion genotyping). To test for differentiation in inversion haplotype frequencies between diapause phenotypes, we ran an exact *G*-test using Genepop 4.1.2 (Raymond and Rousset 1995). *F*_ST_ between individuals expressing facultative and obligate diapause was estimated using VCFtools version 0.1.16 (Danecek et al. 2011) and summarized in 100-kb overlapping windows (20-kb steps).

To check if spruce bark beetle OR genes were associated with inversions, we examined 73 OR genes recently annotated by Yuvaraj et al. (2021). We used OR sequences from Yuvaraj et al. (2021) and mapped them to the spruce bark beetle reference genome using minimap2 (Li 2018). We located 71 out of 73 ORs in the 170 Mb covered by the 26 contigs we analyzed (since contigs with <10,000 SNPs were excluded from inversion detection and genotyping). Three ORs mapped to more than one contig. ItypOR9 and ItypOR58 mapped to the end of IpsContig16 and 23, suggesting possible assembly error and duplication of end-of-contig sequences, which are difficult to assemble. ItypOR59NTE mapped to three nearby (within 7 kb) locations on IpsContig6, suggesting either assembly error or recent duplication. For these three ORs, we used one randomly chosen location in downstream analyses. To test if inversions were significantly enriched in OR genes, we ran a permutation test (10,000 iterations; permutating inversion locations over the 170-Mb genome consisting of 26 contigs, while keeping OR locations fixed). We tested how many permutations yielded a higher (or equal) number of ORs located within inversions than that observed in the real data. To check if alternative inversion arrangements harbored different numbers of OR genes (e.g. if one arrangement carried a deletion), we compared sequence coverage within ORs in individual beetles identified as inversion homozygotes. Additionally, we counted nonsynonymous segregating variants present in ORs situated within inversions and checked if any of these SNPs were highly diverged between inversion haplotypes (by calculating *d*_xy_). Finally, to investigate whether natural selection was involved in shaping nonsynonymous variation patterns in ORs between inversion haplotypes, we calculated *d_N_*/*d_S_* ratio for ORs with multiple highly diverged nonsynonymous variants and performed MacDonald–Kreitman test for ORs with *d_N_*/*d_S_* > 1. *d*_xy_ was calculated using GATK VariantsToTable outputs and custom perl scripts, *d_N_*/*d_S_* was calculated using the pairwise distances option in MEGA11 (syn–nonsynonymous substitution model, Kumar method), and the MacDonald–Kreitman test was performed in DnaSP v6 (Rozas et al. 2017).

### Genotype–environment Association

Genotype–environment association (GEA) analyses were done to test if allele frequency changes in SNPs were associated with the beetle populations' local environment. The analysis was performed using (i) all SNPs present in the analyzed part of the genome and (ii) excluding SNPs present within inversion regions and coding each inversion as single SNPs. Many different environmental variables were summarized along two principal components (see below). To control for the confounding effect of the overall genetic differentiation, we used latent factor mixed models (LFMMs; Caye et al. 2019) as implemented in the lfmm2() function from the R package LEA (Gain and François 2021). We used only SNPs with <20% missing data, MAF ≥ 0.1, and that occurred in individuals with <30% missing data. Because LFMM cannot handle missing data, we imputed missing genotypes using impute() from LEA. We ran LFMM for (i) all SNPs that passed the filters described above and (ii) for a data set where each inversion was represented as a single “SNP” inversion genotype. We used five latent factors (*k* = 5) in lfmm2(). *P*-values were calculated using lfmm2.test() from LEA and false discovery rate (FDR) corrected using the p.adjust() R function with method = “fdr” (Benjamini and Hochberg 1995).

Each beetle population's local environment was characterized according to climate and land cover data. We used all 19 bioclimatic variables from WorldClim version 2.1. (Fick and Hijmans 2017) with a resolution of ∼1 km^2^. These bioclimatic variables included annual mean temperature, mean diurnal range, isothermality, temperature seasonality, maximum temperature of warmest month, minimum temperature of coldest month, temperature annual range, mean temperature of wettest quarter, mean temperature of driest quarter, mean temperature of warmest quarter, mean temperature of coldest quarter, annual precipitation, precipitation of wettest month, precipitation of driest month, precipitation seasonality, precipitation of wettest quarter, precipitation of driest quarter, precipitation of warmest quarter, and precipitation of coldest quarter. These variables are averaged over the years 1970 to 2000. Proportions of forest, cropland, and built-up areas across the geographical range of the spruce bark beetle were downloaded from https://lcviewer.vito.be/ for 2015 with a spatial resolution of ∼100 m^2^ (Buchhorn et al. 2019). These global land cover maps are part of the Copernicus Land Service, derived from PROBA-V satellite observations, and have an accuracy of 80% as measured by the CEOS land product validation subgroup. We also included the proportion of land area covered by spruce trees (genus *Picea*) at a resolution of ∼1 km^2^, as obtained by Brus et al. (2012) using a statistical mapping approach. All bioclimatic variables were reprojected to a final resolution of ∼1 km^2^ using the Lambert azimuthal equal area method. We then extracted mean values for all environmental variables (supplementary fig. S14, Supplementary Material online) within a 50-km radius from each population location using the R package terra (Hijmans 2022). Finally, PCA was used to summarize the multiscale environmental variation among populations. The first two PCA components (PC1 and PC2) explained 25% and 23% of the environmental variation, respectively, and were used as the final input for the GEA analyses. PC1 represented environmental variation mainly related to latitude, with northern populations showing higher values indicative of higher temperature variation and lower temperatures during the coldest months. PC2 represented environmental variation mostly related to temperature and amount of cropland, with higher values representing localities with higher temperatures during the warmest months and a higher proportion of cropland (and conversely less forest cover and spruce; supplementary fig. S15, Supplementary Material online). Additionally, we identified genes closest to SNPs that showed significant association with each PC.

## Supplementary Material

evae263_Supplementary_Data

## Data Availability

All DNA sequences have been deposited to the National Center for Biotechnology Information Sequence Read Archive under the BioProject ID PRJNA1013983. Scripts can be found https://github.com/AnstMykh/Ips_inversions_MS/tree/main.
