# Supplementary material for: Complex Genomic Landscape of Inversion Polymorphism in Europe's Most Destructive Forest Pest
Source: Genome Biol Evol. 2024 Dec 4;16(12):evae263. doi: 10.1093/gbe/evae263 (PMC11652730; doi:10.1093/gbe/evae263)

Supplementary Information

Complex genomic landscape of inversion polymorphism in Europe's most destructive forest pest

Anastasiia Mykhailenko, Piotr Zieliński, Aleksandra Bednarz, Fredrik Schlyter, Martin N. Andersson, Bernardo Antunes, Zbigniew Borowski, Paal Krokene, Markus Melin, Julia Morales-García, Jörg Müller, Zuzanna Nowak, Martin Schebeck, Christian Stauffer, Heli Viiri, Julia Zaborowska, Wiesław Babik, Krystyna Nadachowska-Brzyska

Details on resequencing, data preparation, and filtering

Raw data were first subjected to read quality control using FastQC 0.11.9 (Andrews 2010). Trimming of adaptors, low-quality bases (quality below 3) and reads (below 36 bases long after adaptors and low quality bases trimming) were done with Trimmomatic 0.39 (Bolger et al. 2014). Next, reads were mapped to the *Ips typographus* reference genome (Powell et al. 2021) using Bowtie 2 version 2.4.2 (Langmead and Salzberg 2012). Samtools 1.11 (Bonfield et al. 2021; Danecek et al. 2021) was used for alignment sorting and indexing. Mapping quality was assessed with Qualimap version 2.2.2 (Okonechnikov et al. 2016). To avoid allele amplification bias in variant calling, the Picard MarkDuplicates version 2.24.1 was used to remove duplicate reads (Broad Institute 2019). To detect and correct systematic errors in base quality scores recalibration was done using the Genome Analysis Toolkit (GATK) version 4.1.9.0 BaseRecalibrator and ApplyBQSR0 (McKenna et al. 2010; Depristo et al. 2011). Recalibration was performed separately for individual beetles sequenced on different lanes. This step provided recalibrated alignments which were used in the downstream analyses. A database of known variants was created from the same dataset using GATK hard filtering recommendations (GATK Team). Variants were filtered out when: QD < 2, FS > 60, MQ < 30, SOR > 3, MQRankSum < -12.5, ReadPosRankSum < -8, and, additionally for this purpose, we filtered out variants with MAF < 0.01 and > 50% missing genotypes. Variant calling and genotyping were performed with GATK HaplotypeCaller, CombineGVCFs, and GenotypeGVCFs. HaplotypeCaller was run in GVCF mode using default parameter settings, except for –heterozygosity (value used to compute prior probability that a locus is non-reference) which was set to 0.01 – since we expected increased levels of polymorphisms

compared to the human genome. Next, variant quality score log-odds (VQSLOD) were calculated using GATK VariantRecalibrator and ApplyVQSR with the --truth-sensitivity-filter-level set to 99.0, to take into account properties of the variants not captured in the QUAL score. Bcftools version 1.11 (Bonfield et al. 2021; Danecek et al. 2021) was used to remove insertions and deletions (indels) as well as polymorphic sites five bases up- and downstream from indels. GATK VariantFiltration was applied to mask all genotypes with low sequencing depth (DP < 8) or low genotype quality (GQ < 20). Variants which were not biallelic single nucleotide polymorphisms or did not meet the hard filtering recommendations (see above) were filtered out. To filter out polymorphisms which may come from duplicated regions we removed variants located within masked regions of the genome (Powell et al. 2021), variants with excessive overall coverage (mean + 1 SD), and variants that exhibited significant heterozygote excess (ExcessHet > 54.69). Variants genotyped in less than half of the individuals were also removed. We used PLINK version 1.90b6.18 (--genome; Purcell et al. 2007) to detect sample contaminations, swaps, and duplications, as well as pedigree errors and unknown familial relationships (e.g. sibling pairs present in the data) which might bias downstream analyses. We also removed individuals with excessive coverage (coverage > mean*3) as these could have been affected by manual mistakes during library preparation or pooling. We focused on the 36 largest contigs (longer than 1 Mb) that constituted 78% of the genome assembly (i.e., 186 Mb). Since parts of IpsContig33 had high similarity to mtDNA this contig was not included in the downstream analyses. Genotyping error was assessed using GATK GenotypeConcordance.

After careful filtering of the whole genome re-sequencing data consisting of 253 libraries, we used 240 individuals in downstream analyses. Two individuals were removed due to high relatedness and another two due to extremely high sequencing coverage, indicating potential mistakes during library preparation. Nine individuals that had been sequenced twice allowed us to calculate genotyping errors, particularly Non-Reference Discrepancy which equaled 1.5% and Overall Genotype Concordance which was 99.8% (DePristo et al. 2011). The mean coverage per individual varied from 5x to 53x (mean 23.2; median 23). Coverage at one contig (IpsContig9) was consistently lower (0.57 of average individual coverage) in individuals sexed as males (with only two mismatches (a 0.8% error rate) between morphologically identified sex and IpsContig9 coverage). Thus, IpsContig9 was treated as a sex (X) chromosome. After filtering we retained 5.245 million SNPs in the whole genome but

we analyzed only 5.067 million SNPs present in the 35 analyzed contigs that constituted 75% of the whole genome assembly).

References

Andrews S. 2010. FastQC: a quality control tool for high throughput sequence data.

Bolger AM, Lohse M, Usadel B. 2014. Genome analysis Trimmomatic : a flexible trimmer for Illumina sequence data. *Bioinformatics* 30:2114–2120.

Bonfield JK, Marshall J, Danecek P, Li H, Ohan V, Whitwham A, Keane T. 2021. HTSlib: C library for reading/writing high-Throughput sequencing data. *Gigascience* 10:1–6.

Cabanettes F, Klopp C. 2018. D-GENIES: Dot plot large genomes in an interactive, efficient and simple way. *PeerJ* 2018.

Danecek P, Bonfield JK, Liddle J, Marshall J, Ohan V, Pollard MO, Whitwham A, Keane T, McCarthy SA, Davies RM. 2021. Twelve years of SAMtools and BCFtools. *Gigascience* 10:1–4.

Depristo MA, Banks E, Poplin R, Garimella K V., Maguire JR, Hartl C, Philippakis AA, Del Angel G, Rivas MA, Hanna M, et al. 2011. A framework for variation discovery and genotyping using next-generation DNA sequencing data. *Nat. Genet.* 43:491–501.

Langmead B, Salzberg SL. 2012. Fast gapped-read alignment with Bowtie 2. *Nat. Methods* 9:357–359.

McKenna A, Hanna M, Banks E, Andrey Sivachenko KC, Kernytsky A, Garimella K, Altshuler D, Gabriel S, Daly M, DePristo MA. 2010. The Genome Analysis Toolkit: A MapReduce framework for analyzing next-generation DNA sequencing data. *Genome Res.* 20:1297–1303.

Okonechnikov K, Conesa A, García-Alcalde F. 2016. Qualimap 2: Advanced multi-sample quality control for high-throughput sequencing data. *Bioinformatics* 32:292–294.

Powell D, Groβe-Wilde E, Krokene P, Roy A, Chakraborty A, Löfstedt C, Vogel H, Andersson MN, Schlyter F. 2021. A highly-contiguous genome assembly of the Eurasian spruce bark beetle, *Ips typographus*, provides insight into a major forest pest. *Commun. Biol.* 4:1–9.

Purcell S, Neale B, Todd-Brown K, Thomas L, Ferreira MAR, Bender D, Maller J, Sklar P, De Bakker PIW, Daly MJ, et al. 2007. PLINK: A tool set for whole-genome association and population-based linkage analyses. *Am. J. Hum. Genet.* 81:559–575.

Supplementary Tables:

Table S1 Inversion age estimates in *Ips typographus*. The estimates are based on absolute genetic divergence (d_{xy}) calculated between two inversion haplotypes and using different mutation rates: 2.10 × 10^{-9} (Time 1), 2.90 × 10^{-9} (Time 2), 1.17 × 10^{-8} (Time 3); time in Myr.

**Inversion**	**Contig**	d_{xy}	**Time 1**	**Time 2**	**Time 2**
Inv2	IpsContig2	0.0030	0.70	0.51	0.13
Inv3	IpsContig3	0.0063	1.49	1.08	0.27
Inv5	IpsContig5	0.0076	1.82	1.32	0.33
Inv6	IpsContig6	0.0057	1.35	0.98	0.24
Inv7.1	IpsContig7	0.0100	2.39	1.73	0.43
Inv7.2	IpsContig7	0.0065	1.55	1.12	0.28
Inv9	IpsContig9	0.0033	0.78	0.56	0.14
Inv10	IpsContig10	0.0102	2.63	1.90	0.47
Inv12	IpsContig12	0.0090	1.55	1.12	0.28
Inv13	IpsContig13	0.0101	2.41	1.74	0.43
Inv14.1	IpsContig14	0.0109	2.59	1.87	0.46
Inv14.2	IpsContig14	0.0056	1.34	0.97	0.24
Inv14.3	IpsContig14	0.0123	2.92	2.12	0.52
Inv14.4	IpsContig14	0.0125	2.59	1.88	0.47
Inv14.5	IpsContig14	0.0134	3.20	2.31	0.57
Inv14.6	IpsContig14	0.0036	0.87	0.63	0.16
Inv15	IpsContig15	0.0112	2.67	1.94	0.48
Inv16.1	IpsContig16	0.0094	2.25	1.63	0.40
Inv16.2	IpsContig16	0.0041	0.97	0.70	0.17
Inv17	IpsContig17	0.0126	3.00	2.17	0.54
Inv18	IpsContig18	0.0119	2.83	2.05	0.51
Inv22.1	IpsContig22	0.0124	2.94	2.13	0.53
Inv22.2	IpsContig22	0.0123	2.94	2.13	0.53
Inv22.3	IpsContig22	0.0114	2.70	1.96	0.49
Inv22.4	IpsContig22	0.0121	2.88	2.09	0.52
Inv22.5	IpsContig22	0.0035	0.82	0.60	0.15
Inv23.1	IpsContig23	0.0082	1.96	1.42	0.35
Inv23.2	IpsContig23	0.0035	0.83	0.60	0.15
Inv26	IpsContig26	0.0151	3.59	2.60	0.64

Table S2 Genetic differentiation (F_{ST}) between investigated *Ips typographus* populations. Population abbreviations are according to Table S5. Green color – lower F_{ST}; red – higher F_{ST}.

	MEL	SVA	STJ	LAN	EFI	SIL	AAS	ASA	TON	GOS	LUB	BOR	ROZ	STE	BIL	BAW	TRE
SVA	0.002																
STJ	0	-0.001															
LAN	-0.001	0.001	0														
EFI	0	0.003	0	-0.001													
SIL	0.004	0.005	0.001	0.001	0.001												
AAS	0.003	0.007	0.002	0.001	0.003	0											
ASA	0.004	0.008	0.003	0.003	0.001	0	0.002										
TON	0.001	0.003	-0.001	0.001	0.001	0.001	-0.001	0.002									
GOS	0.006	0.01	0.007	0.005	0.003	0.005	0.007	0.005	0.005								
LUB	0.006	0.01	0.006	0.004	0.003	0.003	0.004	0.005	0.004	-0.001							
BOR	0.003	0.014	0.008	0.005	0.005	0.003	0.005	0.004	0.005	0.004	0.003						
ROZ	0.014	0.02	0.015	0.014	0.014	0.011	0.012	0.012	0.012	0.002	0.003	0.007					
STE	0.017	0.025	0.021	0.02	0.02	0.023	0.022	0.022	0.018	0.009	0.014	0.015	0.008				
BIL	0.029	0.035	0.029	0.026	0.026	0.021	0.021	0.025	0.024	0.009	0.011	0.018	0.003	0.011			
BAW	0.018	0.026	0.024	0.021	0.023	0.023	0.025	0.024	0.023	0.01	0.014	0.013	0.007	0	0.011		
TRE	0.024	0.034	0.027	0.024	0.023	0.021	0.021	0.023	0.022	0.007	0.013	0.014	0.005	0.006	0.001	0.006	
LIN	0.019	0.027	0.023	0.023	0.023	0.025	0.024	0.022	0.021	0.008	0.014	0.015	0.006	0	0.007	0	0.004

Table S3 Association between inversion haplotype and diapause phenotype in *Ips typographus*. Results of the genic differentiation test (exact G test). The test was done using individuals of known diapause phenotype. Only inversions that were genotyped for the whole species range were tested.

Inversion	Contig	p value
Inv3	IpsContig3	1.00
Inv6	IpsContig6	not polymorphic
Inv7.1	IpsContig7	0.74
Inv7.2	IpsContig7	1.00
Inv9	IpsContig9	0.44
Inv10	IpsContig10	0.50
Inv12	IpsContig12	0.15
Inv13	IpsContig13	1.00
Inv14.1	IpsContig14	0.75
Inv14.2	IpsContig14	0.57
Inv14.3	IpsContig14	0.30
Inv14.4	IpsContig14	0.48
Inv14.5	IpsContig14	1.00
Inv14.6	IpsContig14	not polymorphic
Inv15	IpsContig15	1.00
Inv16.1	IpsContig16	0.42
Inv17	IpsContig17	0.31
Inv18	IpsContig18	0.73
Inv22.1	IpsContig22	0.49
Inv22.2	IpsContig22	0.20
Inv22.3	IpsContig22	0.64
Inv22.4	IpsContig22	0.31
Inv22.5	IpsContig22	not polymorphic
Inv23.1	IpsContig23	0.43
Inv26	IpsContig26	0.32

Table S4 Number of nonsynonymous and synonymous SNPs polymorphic in the spruce bark beetle populations. #NS – number of nonsynonymous SNPs; #SS – number of synonymous SNPs. Table sorted by #NS.

OR ID	Contig ID	Inversion ID	#NS	#SS
ItypOR22CTE	IpsContig22	Inv22.3	54	32
ItypOR43JOI	IpsContig5	Inv5	50	39
ItypOR53	IpsContig5	Inv5	44	40
ItypOR3	IpsContig5	Inv5	43	33
ItypOR2	IpsContig5	Inv5	42	35
ItypOR49	IpsContig13	Inv13	41	40
ItypOR19	IpsContig5	Inv5	38	30
ItypOR30	IpsContig16	Inv16.1, Inv16.2	38	14
ItypOR4	IpsContig5	Inv5	38	37
ItypOR36	IpsContig14	Inv14, Inv14.6	37	44
ItypOR50	IpsContig5	Inv5	36	25
ItypOR20NTE	IpsContig14	Inv14, Inv14.6	35	37
ItypOR1	IpsContig7	Inv7.2	31	22
ItypOR17	IpsContig7	Inv7.2	31	37
ItypOR33	IpsContig5	Inv5	31	26
ItypOR40	IpsContig5	Inv5	29	16
ItypOR23	IpsContig13	Inv13	28	24
ItypOR28	IpsContig13	Inv13	28	37
ItypOR31	IpsContig16	Inv16.1, Inv16.2	22	38
ItypOR35JF	IpsContig16	Inv16.1, Inv16.2	22	12
ItypOR10	IpsContig5	Inv5	20	14
ItypOR27	IpsContig13	Inv13	19	37
ItypOR41	IpsContig5	Inv5	17	14
ItypOR29	IpsContig5	Inv5	15	10
ItypOR16	IpsContig16	Inv16.1, Inv16.2	12	13
ItypOR11	IpsContig16	Inv16.1, Inv16.2	11	2

ItypOR34	IpsContig5	Inv5	10	12
ItypOR52NTE	IpsContig5	Inv5	7	6
ItypOR18JF	IpsContig14	Inv14, Inv14.6	0	0
ItypOR44	IpsContig14	Inv14, Inv14.6	0	0
ItypOR47	IpsContig5	Inv5	0	0
ItypOR58	IpsContig16	Inv16.1, Inv16.2	0	0
ItypOR58	IpsContig23	Inv23.1, Inv23.2	0	0
ItypOR9	IpsContig16	Inv16.1, Inv16.2	0	0
ItypOR9	IpsContig23	Inv23.1, Inv23.2	0	0

Table S5 Nonsynonymous divergence between inversion haplotypes within OR coding sequence. Numbers indicate number of nonsynonymous SNPs with different d_{xy} thresholds. Table sorted by number of SNPs with d_{xy} >0.9.

OR ID	Contig ID	Inversion ID	d_{xy} >0.9	d_{xy} >0.8	d_{xy} >0.5	d_{xy} <0.1
ItypOR43JOI	IpsContig5	Inv5	7	7	7	41
ItypOR30	IpsContig16	Inv16.1	4	4	4	30
ItypOR20NTE	IpsContig14	Inv14.1	3	3	5	25
ItypOR36	IpsContig14	Inv14.1	3	4	4	31
ItypOR23	IpsContig13	Inv13	2	2	5	18
ItypOR31	IpsContig16	Inv16.1	2	4	4	17
ItypOR16	IpsContig16	Inv16.1	1	1	1	11
ItypOR27	IpsContig13	Inv13	1	2	3	11
ItypOR29	IpsContig5	Inv5	1	1	1	12
ItypOR1	IpsContig7	Inv7.2	0	0	1	26
ItypOR10	IpsContig5	Inv5	0	0	1	17
ItypOR11	IpsContig16	Inv16.2	0	0	0	9
ItypOR11	IpsContig16	Inv16.1	0	0	2	9
ItypOR16	IpsContig16	Inv16.2	0	0	1	11
ItypOR17	IpsContig7	Inv7.2	0	0	0	21
ItypOR19	IpsContig5	Inv5	0	0	1	33
ItypOR2	IpsContig5	Inv5	0	1	1	35
ItypOR20NTE	IpsContig14	Inv14.6	0	0	1	27
ItypOR22CTE	IpsContig22	Inv22.3	0	4	14	23
ItypOR28	IpsContig13	Inv13	0	0	2	24
ItypOR3	IpsContig5	Inv5	0	1	11	32
ItypOR30	IpsContig16	Inv16.2	0	0	3	32
ItypOR31	IpsContig16	Inv16.2	0	0	2	17
ItypOR33	IpsContig5	Inv5	0	0	1	28
ItypOR34	IpsContig5	Inv5	0	0	0	8
ItypOR35JF	IpsContig16	Inv16.2	0	0	0	22

ItypOR35JF	IpsContig16	Inv16	0	0	0	19
ItypOR36	IpsContig14	Inv14.6	0	0	1	34
ItypOR4	IpsContig5	Inv5	0	0	0	35
ItypOR40	IpsContig5	Inv5	0	1	3	24
ItypOR41	IpsContig5	Inv5	0	1	1	16
ItypOR49	IpsContig13	Inv13	0	0	0	39
ItypOR50	IpsContig5	Inv5	0	1	3	31
ItypOR52NTE	IpsContig5	Inv5	0	0	0	7
ItypOR53	IpsContig5	Inv5	0	0	2	34

Table S6 Gene Ontology categories significantly enriched in inversions that correlate with latitude. MF – Molecular function; BP – Biological process; CC – cellular component. Categories associated with olfaction are bolded.

GO ID	Term	p-value	type
GO:0006289	nucleotide-excision repair	0.008	BP
GO:0050911	**detection of chemical stimulus involved in sensory perception of smell**	0.010	BP
GO:0004984	**olfactory receptor activity**	0.011	MF
GO:0009116	nucleoside metabolic process	0.012	BP
GO:0004222	metalloendopeptidase activity	0.016	MF
GO:0042302	structural constituent of cuticle	0.021	MF
GO:0032559	adenyl ribonucleotide binding	0.024	MF
GO:0032508	DNA duplex unwinding	0.029	BP
GO:0009966	regulation of signal transduction	0.030	BP
GO:0097435	supramolecular fiber organization	0.031	BP
GO:0032561	guanyl ribonucleotide binding	0.031	MF
GO:0007264	small GTPase-mediated signal transduction	0.033	BP
GO:0003678	DNA helicase activity	0.036	MF
GO:0009161	ribonucleoside monophosphate metabolic process	0.037	BP
GO:0009124	nucleoside monophosphate biosynthetic process	0.037	BP
GO:0006886	intracellular protein transport	0.040	BP
GO:0006468	protein phosphorylation	0.043	BP
GO:0006367	transcription initiation at RNA polymerase II promoter	0.044	BP
GO:0034032	purine nucleoside bisphosphate metabolic process	0.044	BP
GO:0033875	ribonucleoside bisphosphate metabolic process	0.044	BP
GO:0022857	transmembrane transporter activity	0.045	MF
GO:0043168	anion binding	0.046	MF
GO:0003690	double-stranded DNA binding	0.047	MF

Table S7 Gene Ontology categories significantly enriched in inversions. MF – Molecular function; BP – Biological process; CC – cellular component. Categories associated with olfaction indicated are bolded.

**GO ID**	**Term**	**p-value**	**type**
GO:0005975	carbohydrate metabolic process	0.0000061	BP
GO:0016491	oxidoreductase activity	0.0000091	MF
GO:0071555	cell wall organization	0.000038	BP
GO:0016853	isomerase activity	0.000065	MF
GO:0030286	dynein complex	0.00022	CC
GO:0005509	calcium ion binding	0.00082	MF
GO:0006886	intracellular protein transport	0.0015	BP
GO:0022857	transmembrane transporter activity	0.00158	MF
GO:0005524	ATP binding	0.00225	MF
GO:0006355	regulation of DNA-templated transcription	0.0027	BP
GO:0000287	magnesium ion binding	0.0039	MF
GO:0020037	heme binding	0.00405	MF
GO:0043547	positive regulation of GTPase activity	0.0042	BP
GO:0005096	GTPase activator activity	0.00435	MF
GO:0015631	tubulin binding	0.00454	MF
GO:0016747	acyltransferase activity. transferring groups other than amino-acyl groups	0.00522	MF
GO:0006457	protein folding	0.0061	BP
GO:0004553	hydrolase activity. hydrolyzing O-glycosyl compounds	0.00618	MF
GO:0016705	oxidoreductase activity. acting on paired donors. with incorporation or reduction of molecular oxygen	0.00713	MF
GO:0005506	iron ion binding	0.00714	MF
GO:0003777	microtubule motor activity	0.00922	MF
GO:0016810	hydrolase activity. acting on carbon-nitrogen (but not peptide) bonds	0.00929	MF
GO:0006811	monoatomic ion transport	0.0101	BP
GO:0003723	RNA binding	0.01011	MF
GO:0016773	phosphotransferase activity. alcohol group as acceptor	0.01052	MF
GO:0008234	cysteine-type peptidase activity	0.0111	MF
GO:0055085	transmembrane transport	0.0125	BP
GO:0035556	intracellular signal transduction	0.0148	BP
GO:0019438	aromatic compound biosynthetic process	0.0165	BP
GO:0005319	lipid transporter activity	0.01735	MF
GO:0016667	oxidoreductase activity. acting on a sulfur group of donors	0.01735	MF
GO:0006468	protein phosphorylation	0.0176	BP
GO:0004812	aminoacyl-tRNA ligase activity	0.01774	MF

GO:0005829	cytosol	0.01818	CC
GO:1901576	organic substance biosynthetic process	0.0192	BP
GO:0005783	endoplasmic reticulum	0.01972	CC
GO:0050896	response to stimulus	0.0205	BP
GO:0016579	protein deubiquitination	0.0206	BP
**GO:0050911**	**detection of chemical stimulus involved in sensory perception of smell**	**0.0206**	**BP**
**GO:0004984**	**olfactory receptor activity**	**0.02136**	**MF**
GO:0004497	monooxygenase activity	0.02147	MF
GO:0015914	phospholipid transport	0.0228	BP
GO:0007155	cell adhesion	0.0228	BP
GO:0140096	catalytic activity. acting on a protein	0.02711	MF
GO:0016798	hydrolase activity. acting on glycosyl bonds	0.02754	MF
GO:0015078	proton transmembrane transporter activity	0.02821	MF
GO:0043565	sequence-specific DNA binding	0.02949	MF
GO:0005794	Golgi apparatus	0.02985	CC
GO:0030054	cell junction	0.03063	CC
GO:0003700	DNA-binding transcription factor activity	0.03085	MF
GO:0018130	heterocycle biosynthetic process	0.031	BP
GO:0044249	cellular biosynthetic process	0.0327	BP
GO:0006418	tRNA aminoacylation for protein translation	0.0344	BP
GO:1901362	organic cyclic compound biosynthetic process	0.0361	BP
GO:0005737	cytoplasm	0.03646	CC
GO:0005886	plasma membrane	0.03892	CC
GO:0046496	nicotinamide nucleotide metabolic process	0.0394	BP
GO:0044272	sulfur compound biosynthetic process	0.0394	BP
GO:0003690	double-stranded DNA binding	0.04048	MF
GO:0050790	regulation of catalytic activity	0.0431	BP
GO:0000398	mRNA splicing. via spliceosome	0.044	BP
GO:0018193	peptidyl-amino acid modification	0.0442	BP
GO:0008810	cellulase activity	0.04579	MF
GO:0015291	secondary active transmembrane transporter activity	0.04579	MF
GO:0006396	RNA processing	0.0469	BP
GO:0140513	nuclear protein-containing complex	0.04851	CC
GO:1902600	proton transmembrane transport	0.049	BP
GO:0007264	small GTPase-mediated signal transduction	0.049	BP

Table S8 Genes containing SNPs significant in the genotype-environment association analysis.

Gene	Description	GO ID
Ityp00693	roundabout homolog 2-like	NA
Ityp07355	PREDICTED: mitoferrin-1-like	GO:0016021
Ityp07620	U2 small nuclear ribonucleoprotein auxiliary factor 35 kDa subunit-related protein 1	NA
Ityp08738	poly [ADP-ribose] polymerase 12-like	NA
Ityp08741	general transcription factor IIE subunit 2-like	GO:0003677.GO:0005673.GO:0006367
Ityp09110	inotocin receptor	GO:0005000.GO:0005887.GO:0007186.GO:0032870.GO:0042277
Ityp14927	copper-transporting ATPase 1 isoform X1	NA
Ityp17124	PREDICTED: uncharacterized protein LOC109541129	NA
Ityp17174	chondroitin sulfate proteoglycan 4 isoform X2	GO:0016491.GO:0055114
Ityp17175	eukaryotic translation initiation factor 2-alpha kinase	NA
Ityp17177	PREDICTED: uncharacterized protein LOC109540861 isoform X1	NA
Ityp17181	RNA-binding protein 24-A-like	GO:0003729
Ityp17183	solute carrier organic anion transporter family member 2A1-like	NA
Ityp17184	PREDICTED: uncharacterized protein LOC109541131	NA

Table S9. Significant deviations from Hardy-Weinberg expectations for inversion genotypes at whole species range and within genetic groups (southern, northern and Polish). Significance level at 0.05 after Bonferroni correction.

Inversion	Data	Observed number of genotypes						Expected number of genotypes						P-value	Alpha at 0.05
		AA	AB	AC	BB	BC	CC	AA	AB	AC	BB	BC	CC		
Inv12	whole species range	18	55		167			9	74		158			0.00022	0.001724
Inv22.4		46	101	88	3	2	0	82	64	53	12	20	8	0.00000	0.001724
Inv22.4	northern	28	45	47	0	0	0	46	28	29	4	9	5	0.00000	0.000617
Inv22.4	polish	8	20	24	2	0	0	17	13	13	3	5	3	0.00000	0.000617
Inv22.4	southern	10	36	17	1	2	0	20	22	11	6	6	1	0.00000	0.000617

Table S10 Mutation load analyses in *Ips typographus*.

The ratio of nonsynonymous to synonymous nucleotide diversity (π_N/π_S) was calculated in 200 kb windows along the genome. The windows were separated into windows inside or outside inversions. Windows of the collinear part of the genome were analyzed including (no strict filtering) or excluding windows that come from the contigs harboring inversions (strict filtering). Mutation load for inversions was calculated for all individuals (all), individuals that were homozygous for less common inversion haplotypes (minor), and individuals that were homozygous for more common inversion haplotypes (major). Different filtering criteria were tested. including minimum number of genes (gene), minimum number of individuals carrying a given haplotype (ind), and minimum number of windows (win). For further details see the main text.

Strict filtering for collinear genome	Inversion groups	Filtering criteria gene/ind/win	Median π_N/π_S		P-value
			for collinear genome	for inversion group	
no	all	5/4/4	0.1311	0.1161	0.001
	minor		0.1311	0.1224	0.049
	major		0.1311	0.1131	<0.001
	all	10/10/4	0.1293	0.1145	0.006
	minor		0.1293	0.1161	0.537
	major		0.1293	0.1129	<0.001
yes	all	5/4/4	0.1285	0.1161	0.047
	minor		0.1285	0.1224	0.216
	major		0.1285	0.1131	0.016
	all	10/10/4	0.1284	0.1145	0.062
	minor		0.1284	0.1161	0.663
	major		0.1284	0.1129	<0.001

Table S11 Details about sites where *Ips typographus* were collected. ‘Group’ denotes in what part of the species’ distribution area beetles were collected: northern (N), southern (S) or Poland (P). ‘N’ gives the number of individuals collected per site.

Population ID	Population name	Latitude	Longitude	Country	Group	N
AAS	Ås	59.667	10.793	Norway	N	13
ASA	Asa	57.165	14.783	Sweden	N	14
BAW	Bavarian Forest	48.960	13.395	Germany	S	11
BIL	Bílkovice	49.761	14.848	Czechia	S	14
BOR	Borki	54.090	21.912	Poland	P	12
EFI	Eastern Finland	62.492	30.010	Finland	N	13
GOS	Gościnno	54.047	15.657	Poland	P	14
LAN	Länsi	61.723	23.633	Finland	N	13
LIN	Linz	48.092	13.874	Austria	S	14
LUB	Lubaszki	54.057	17.556	Poland	P	14
MEL	Mellakoski	66.399	24.440	Finland	N	14
ROZ	Roztocze	50.508	22.786	Poland	P	14
SIL	Siljanfors	60.757	14.066	Sweden	N	14
STE	Steigerwald	49.622	10.263	Germany	S	14
STJ	Stjørdal	63.469	10.918	Norway	N	13
SVA	Svartberget	64.236	19.570	Sweden	N	13
TON	Tönnersjö	56.643	13.070	Sweden	N	13
TRE	Třebíč	49.212	15.879	Czechia	S	13

Table S12. Loadings of environmental variables in the first two principal components.

Variable	Alias	Weight PC1	Weight PC2
Temperature Seasonality	bio_4	0.284	-0.033
Temperature Annual Range	bio_7	0.281	-0.067
Tree fraction	tree	0.193	-0.242
Precipitation Seasonality	bio_15	0.104	0.166
Mean Diurnal Range	bio_2	0.084	-0.039
Percentage Picea	picea	-0.011	-0.286
Max Temperature of Warmest Month	bio_5	-0.038	0.309
Mean Temperature of Wettest Quarter	bio_8	-0.038	0.235
Mean Temperature of Warmest Quarter	bio_10	-0.116	0.309
Cropland fraction	crops	-0.151	0.297
Elevation	elevation	-0.2	-0.117
Precipitation of Coldest Quarter	bio_19	-0.207	-0.237
Precipitation of Warmest Quarter	bio_18	-0.212	-0.185
Precipitation of Wettest Month	bio_13	-0.218	-0.214
Precipitation of Wettest Quarter	bio_16	-0.22	-0.213
Annual Precipitation	bio_12	-0.223	-0.243
Built-up fraction	urban	-0.223	0.138
Annual Mean Temperature	bio_1	-0.229	0.237
Precipitation of Driest Quarter	bio_17	-0.237	-0.222
Precipitation of Driest Month	bio_14	-0.238	-0.213
Mean Temperature of Driest Quarter	bio_9	-0.239	0.082
Isothermality	bio_3	-0.245	0.033
Min Temperature of Coldest Month	bio_6	-0.263	0.172
Mean Temperature of Coldest Quarter	bio_11	-0.267	0.17

Supplementary Figures

Figure S1 Comparison of *Ips typographus* and its close relative - *I. nitidius* – genomes, based on synteny analysis of two assemblies. The 36 longest contigs of focal species are contrasted with 16 *I. nitidius* pseudochromosomes, denoted as IT1 – IT36 and IN1 – IN16, respectively. Contigs that have reverse orientation in assembly are indicated with 'r' subscript and were inverted for image clarity. Black bars correspond to contig sequences, grey lines show colinear synteny blocks and red lines correspond to fragments with opposite orientations. Inversions in *I. typographus* genome found in this study are indicated as thicker red bars on upper panel, if there were two or more overlapping inversions on the same contig, they are shown as partially transparent.

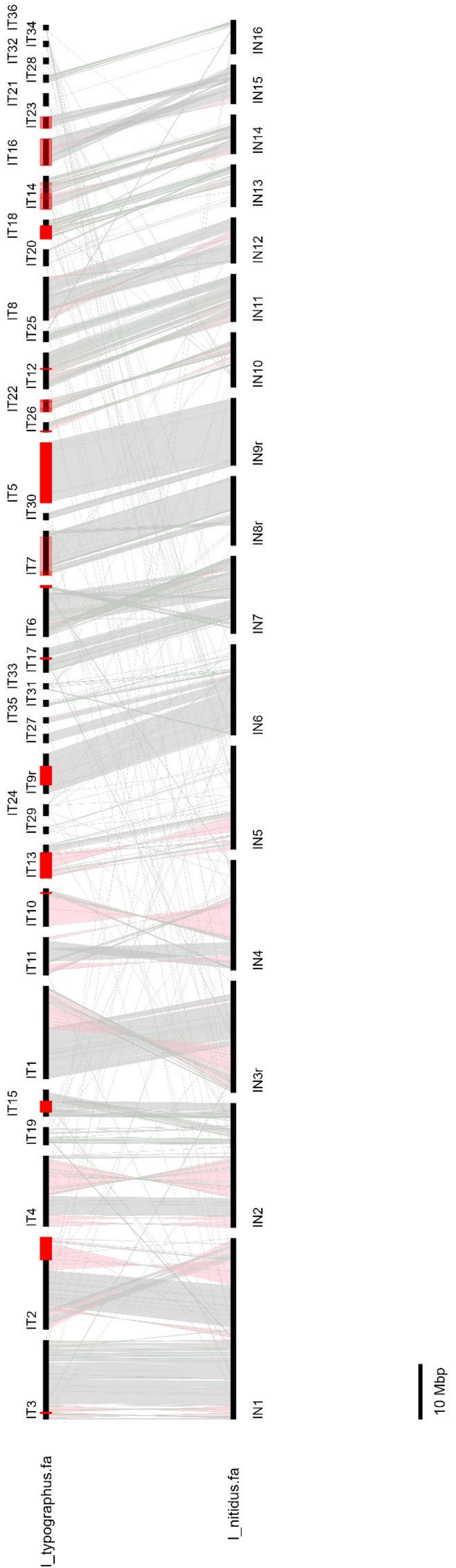
I_typographus.fa
I_nitidus.fa
IT3
IT2
IT4
IT19
IT15
IT1
IT11
IT10
IT13
IT29
IT24
IT9r
IT27
IT35
IT31
IT33
IT17
IT6
IT7
IT30
IT5
IT26
IT22
IT12
IT25
IT8
IT20
IT18
IT14
IT16
IT23
IT21
IT28
IT32
IT34
IT36
IN1
IN2
IN3r
IN4
IN5
IN6
IN7
IN8r
IN9r
IN10
IN11
IN12
IN13
IN14
IN15
IN16
10 Mbp

Figure S2 Inversion patterns in the *Ips typographus* genome. Each subplot shows multiple lines of evidence for inversion polymorphism: localPCA (A), per contig PCA (B), PCA for putative inversion region only (color coded by population (C) and heterozygosity (D)), F_{ST} between inversion haplotypes (E), LD patterns for homozygous (upper triangle) and all individuals (bottom triangle) (F); population recombination rate for three inversion genotypes (G). (A) Identification of inversion coordinates with MDS, where MDS coordinates are plotted against the length of the contig in Mb. Y-axis title shows on which dimension a given inversion was identified as an outlier. Each dot represents a single window, red dots - outlier inversion windows, grey dots - rest of the contig. (B) PCA plot for the whole contig; each dot is an individual colored according to a population it belongs to, blue colors represent populations from northern group, yellow colors – populations from Polish group and red and green colors – populations from southern group. (C) PCA plot for the inverted region each dot is an individual colored the same way as on the panel B. (D) PCA plot for the inverted region, each dot is colored according to the level of the observed heterozygosity in the inverted region. (E) Genetic differentiation (F_{ST}) between inversion haplotypes (major – MJ and minor MN inversion haplotype, and recombinant haplotype R, if present) along the contig. (F) Contig LD patterns for homozygous (upper triangle) and all individuals (bottom triangle). (G) Population recombination rate (ρ) calculated along the contig between inversion genotypes: M J – homozygotes of the more frequent haplotype. MN – homozygotes of the less frequent haplotype and HET - heterozygotes.

Inv 2

Inv2 could be genotyped only in southern group and C and D PCAs are shown only for southern populations for clearer visualization.

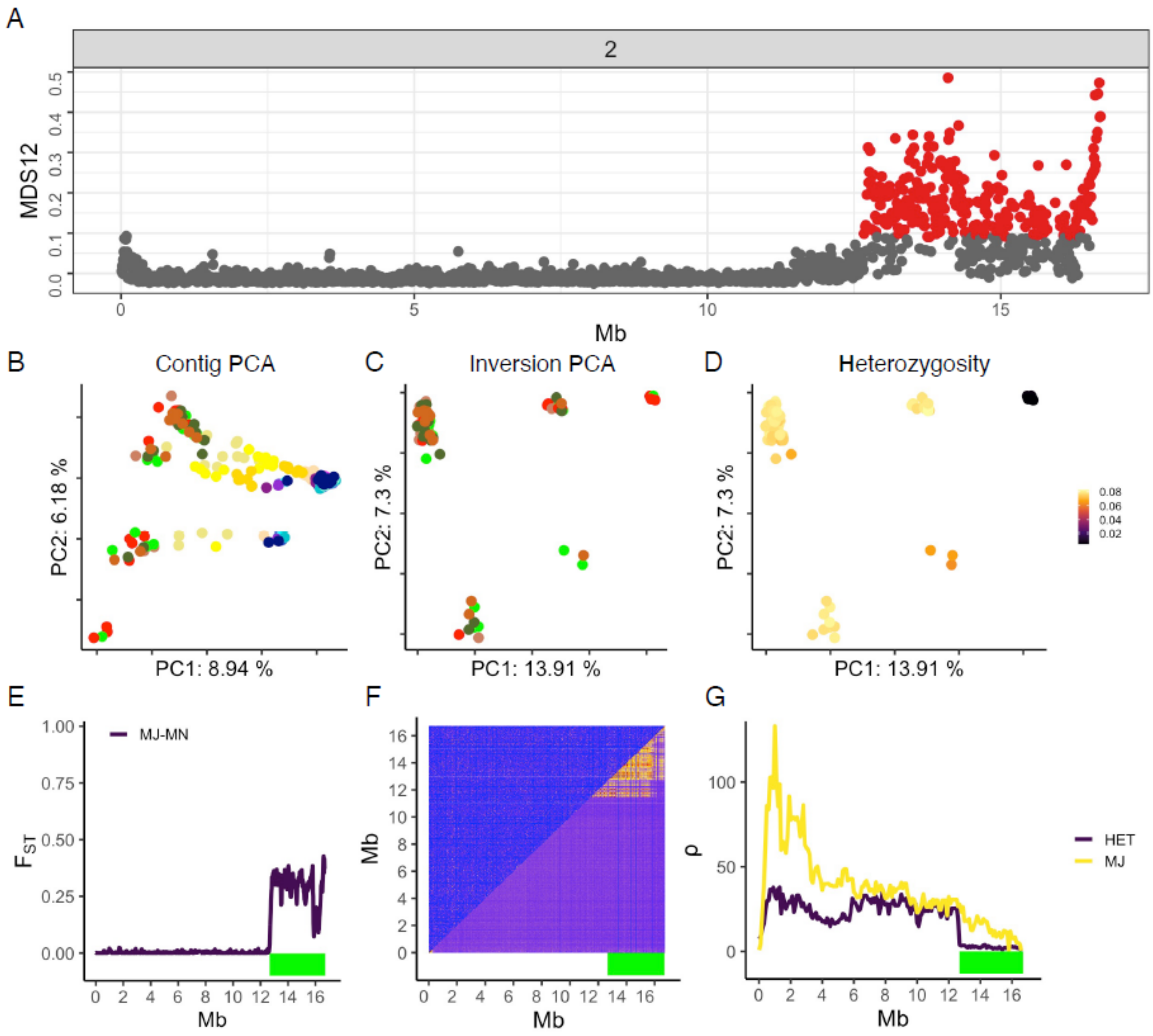

Inv3

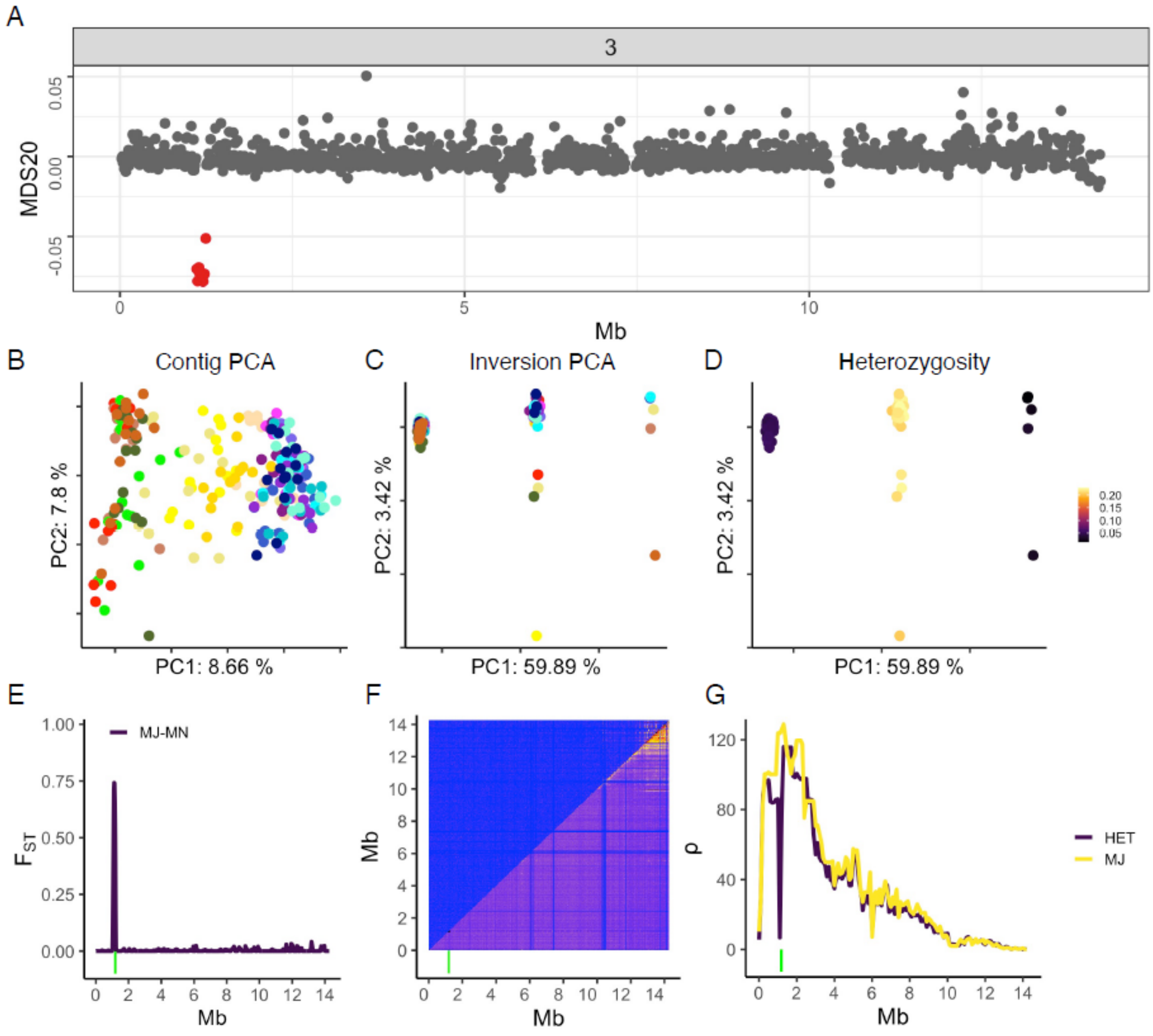
A
3
MDS20
Mb
B
Contig PCA
PC2: 7.8 %
PC1: 8.66 %
C
Inversion PCA
PC2: 3.42 %
PC1: 59.89 %
D
Heterozygosity
PC2: 3.42 %
PC1: 59.89 %
0.20
0.15
0.10
0.05
E
MJ-MN
F_{ST}
Mb
F
Mb
Mb
G
ρ
Mb
HET
MJ

Inv5

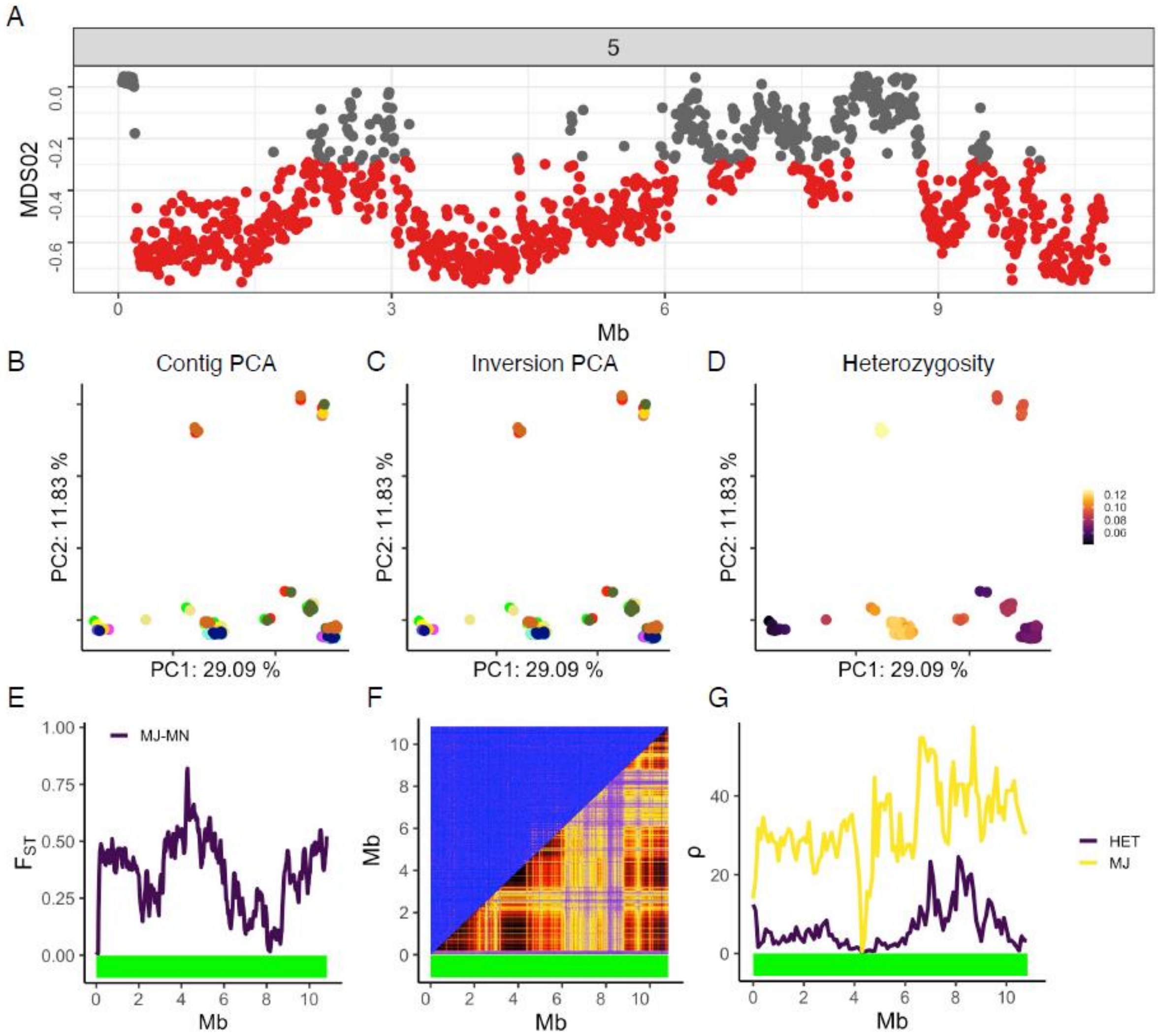

A
5
MDS02
0.0
-0.2
-0.4
-0.6
0
3
6
9
Mb
B
Contig PCA
C
Inversion PCA
D
Heterozygosity
PC2: 11.83 %
PC1: 29.09 %
0.12
0.10
0.08
0.06
E
MJ-MN
F_{ST}
1.00
0.75
0.50
0.25
0.00
F
G
HET
MJ
ρ
40
20
0
2
4
6
8
10
Mb

Inv6

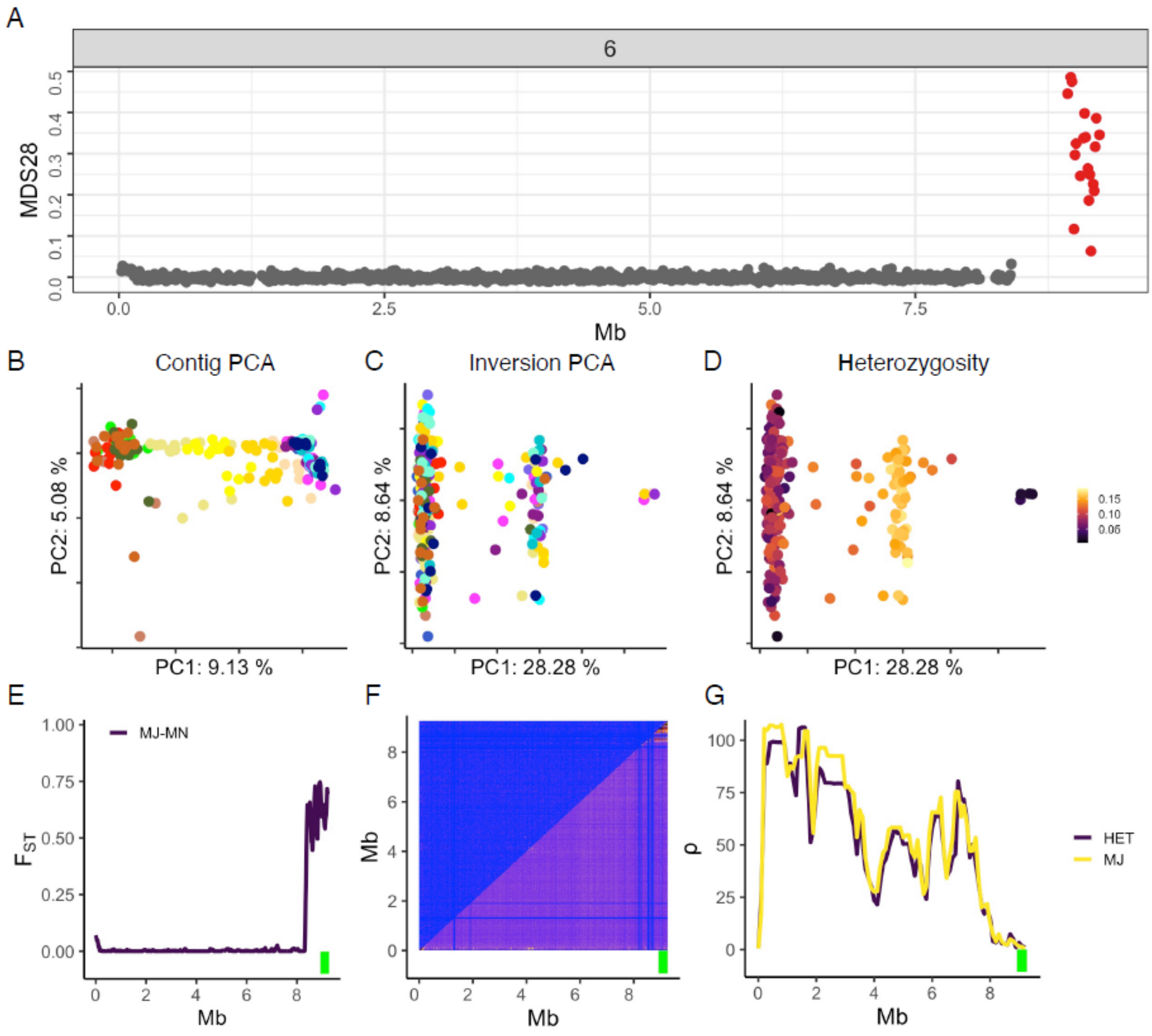
A
6
MDS28
Mb
B
Contig PCA
PC2: 5.08 %
PC1: 9.13 %
C
Inversion PCA
PC2: 8.64 %
PC1: 28.28 %
D
Heterozygosity
PC2: 8.64 %
PC1: 28.28 %
0.15
0.10
0.05
E
MJ-MN
F_{ST}
Mb
F
Mb
Mb
G
ρ
HET
MJ
Mb

Inv7.1

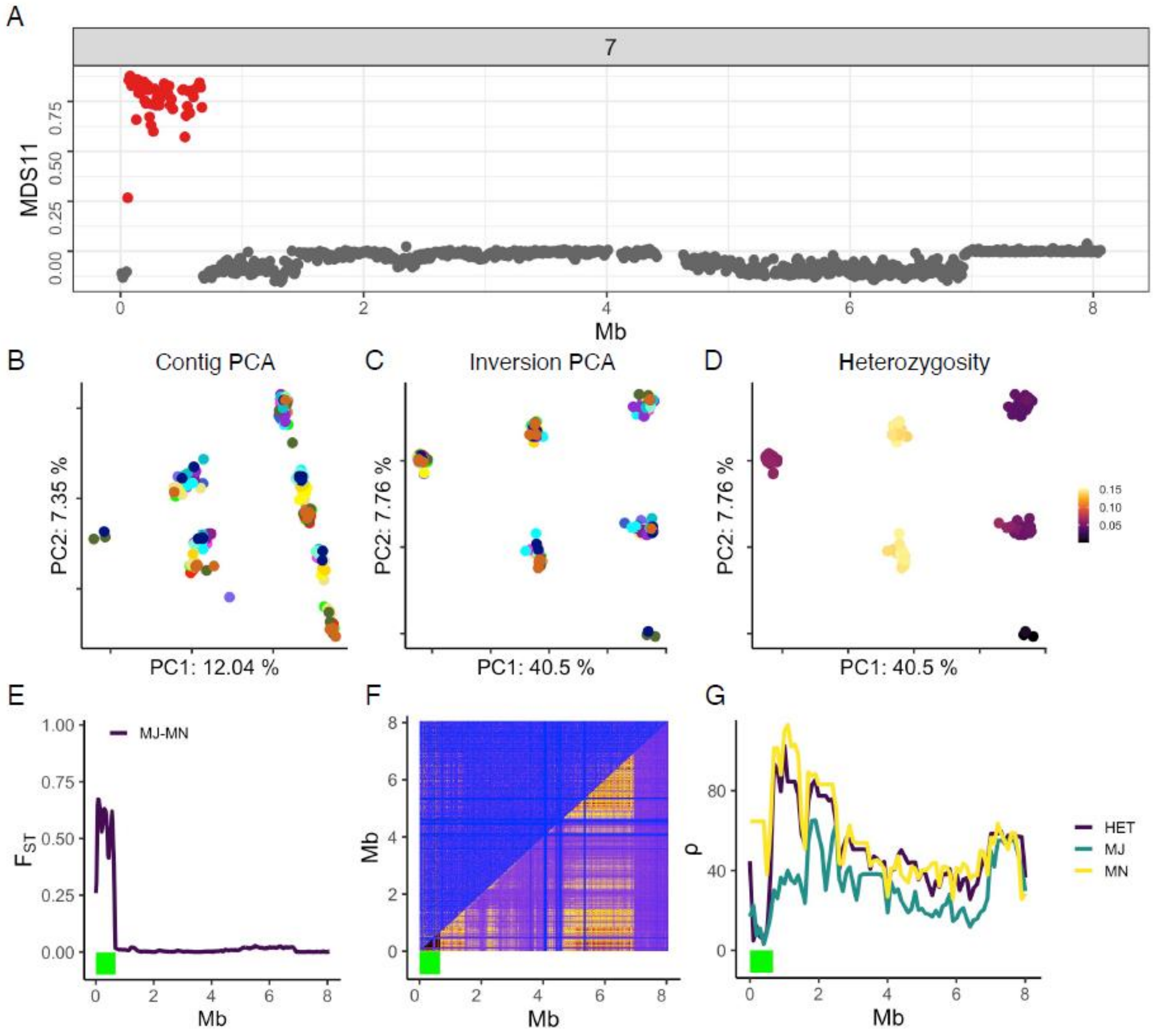

A
7
MDS11
0.00
0.25
0.50
0.75
0
2
4
6
8
Mb
B
Contig PCA
PC2: 7.35 %
PC1: 12.04 %
C
Inversion PCA
PC2: 7.76 %
PC1: 40.5 %
D
Heterozygosity
PC2: 7.76 %
PC1: 40.5 %
0.15
0.10
0.05
E
MJ-MN
F_{ST}
1.00
0.75
0.50
0.25
0.00
Mb
F
Mb
Mb
G
ρ
80
40
0
HET
MJ
MN
Mb

Inv7.2

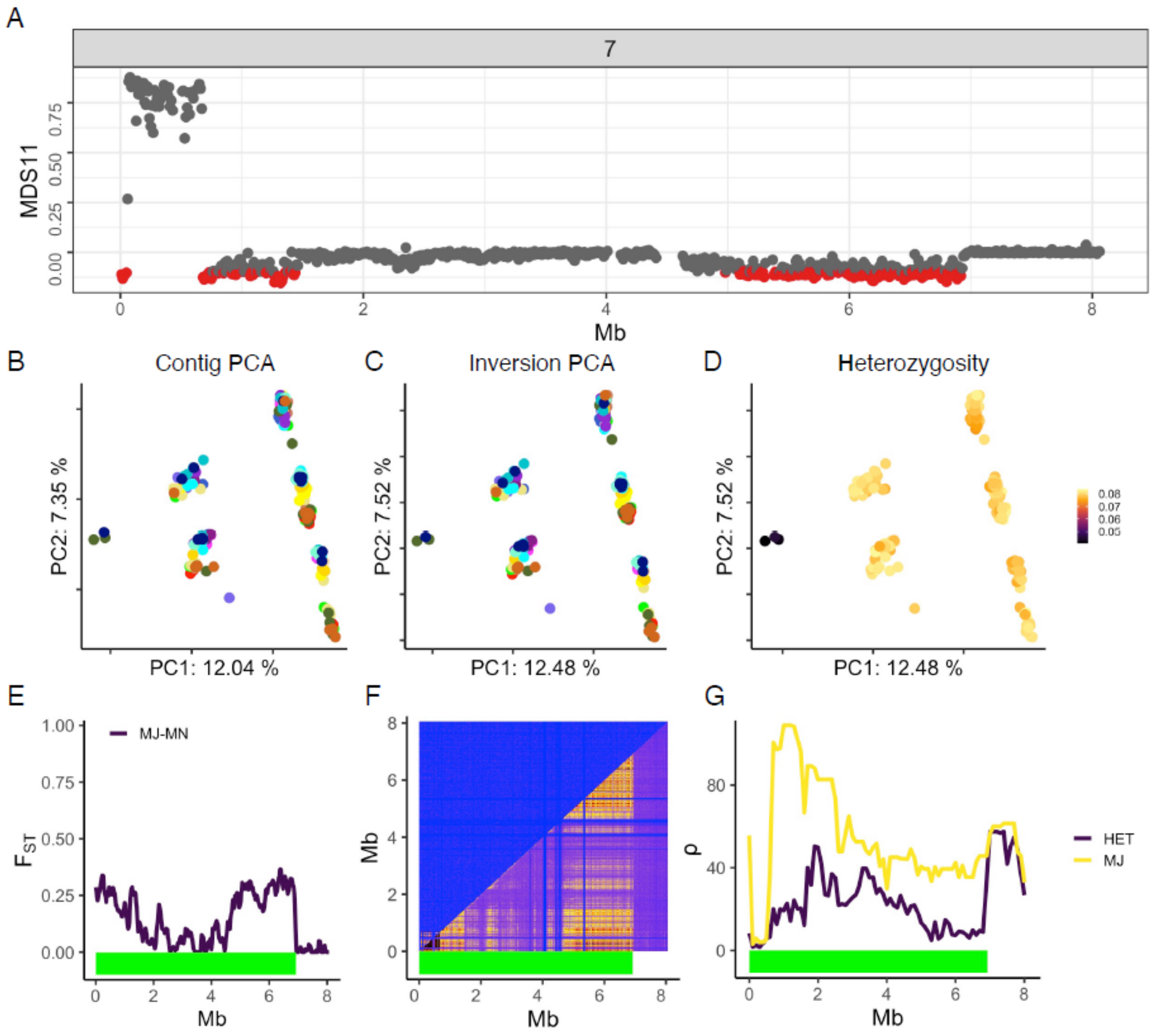

A
7
MDS11
Mb
B
Contig PCA
PC2: 7.35 %
PC1: 12.04 %
C
Inversion PCA
PC2: 7.52 %
PC1: 12.48 %
D
Heterozygosity
PC2: 7.52 %
PC1: 12.48 %
E
MJ-MN
F_{ST}
Mb
F
Mb
Mb
G
ρ
HET
MJ
Mb

Inv9

Inv9 is only polymorphic in northern group and PCAs are shown only for northern populations for clearer visualization. Population recombination rate was calculated using females only, as this is a putative X chromosome.

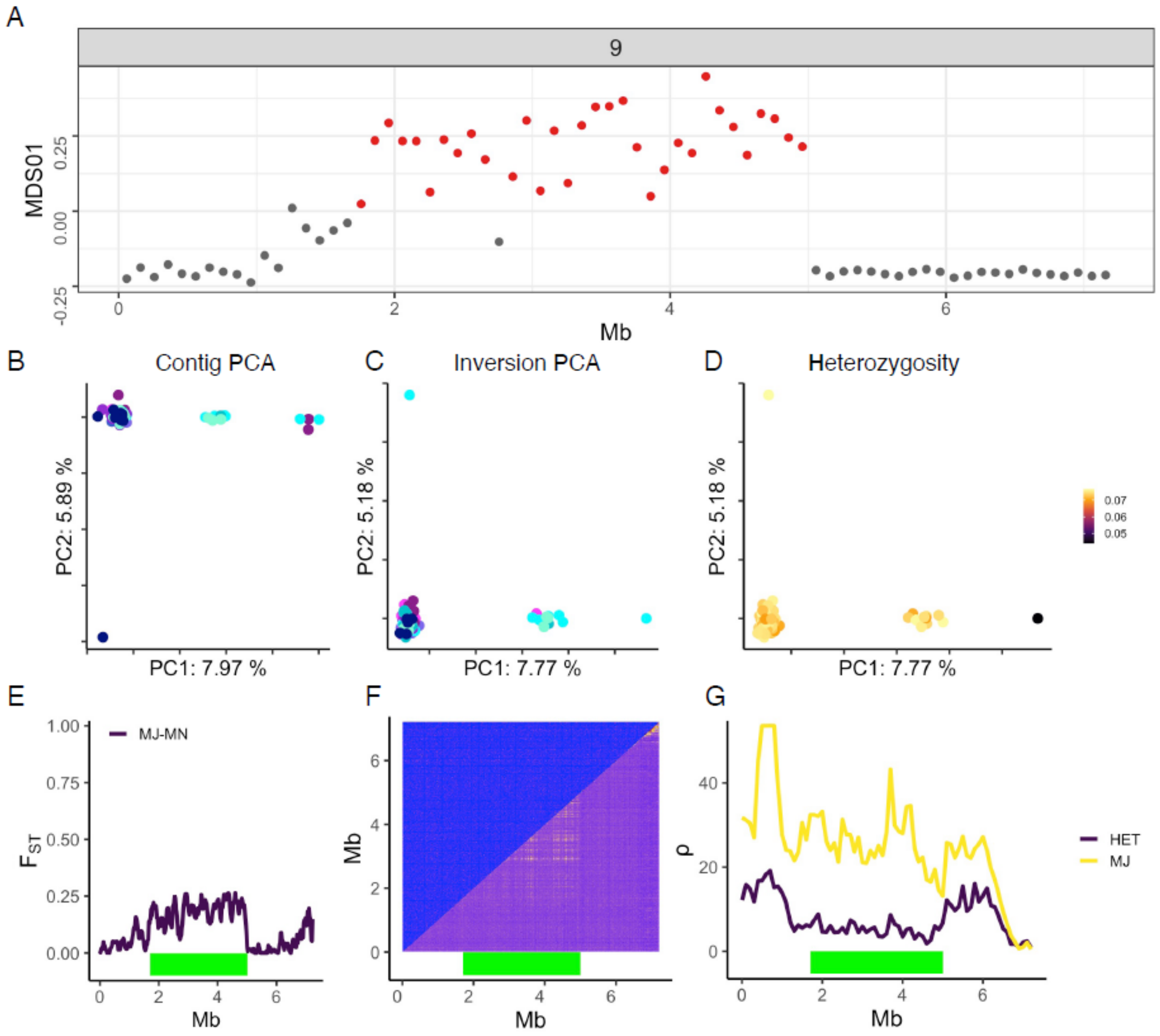

Inv10

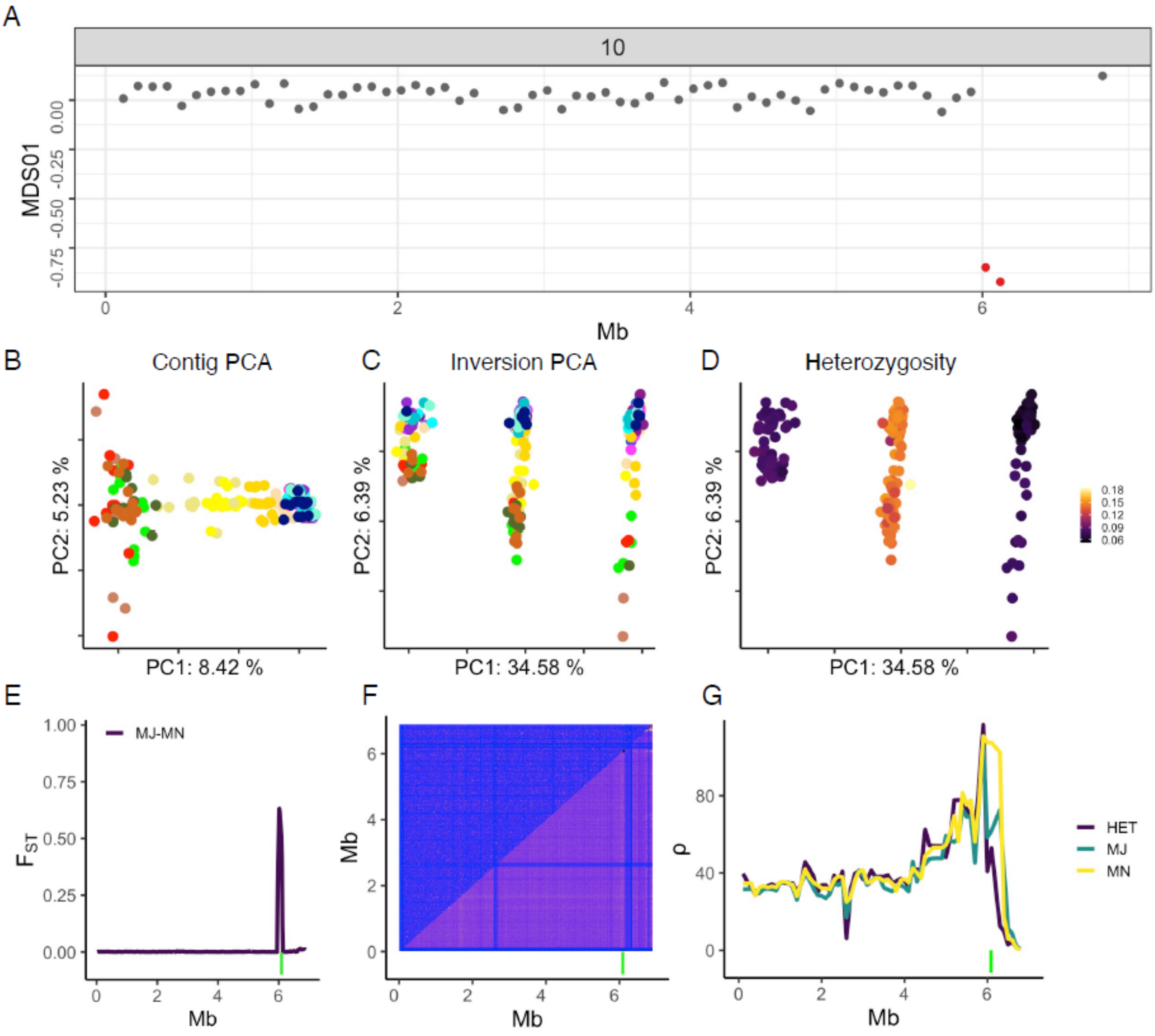

A
10
MDS01
0.00
-0.25
-0.50
-0.75
0
2
4
6
Mb
B
Contig PCA
PC2: 5.23 %
PC1: 8.42 %
C
Inversion PCA
PC2: 6.39 %
PC1: 34.58 %
D
Heterozygosity
PC2: 6.39 %
PC1: 34.58 %
0.18
0.15
0.12
0.09
0.06
E
MJ-MN
F_{ST}
1.00
0.75
0.50
0.25
0.00
Mb
F
Mb
Mb
G
ρ
80
40
0
HET
MJ
MN
Mb

Inv12

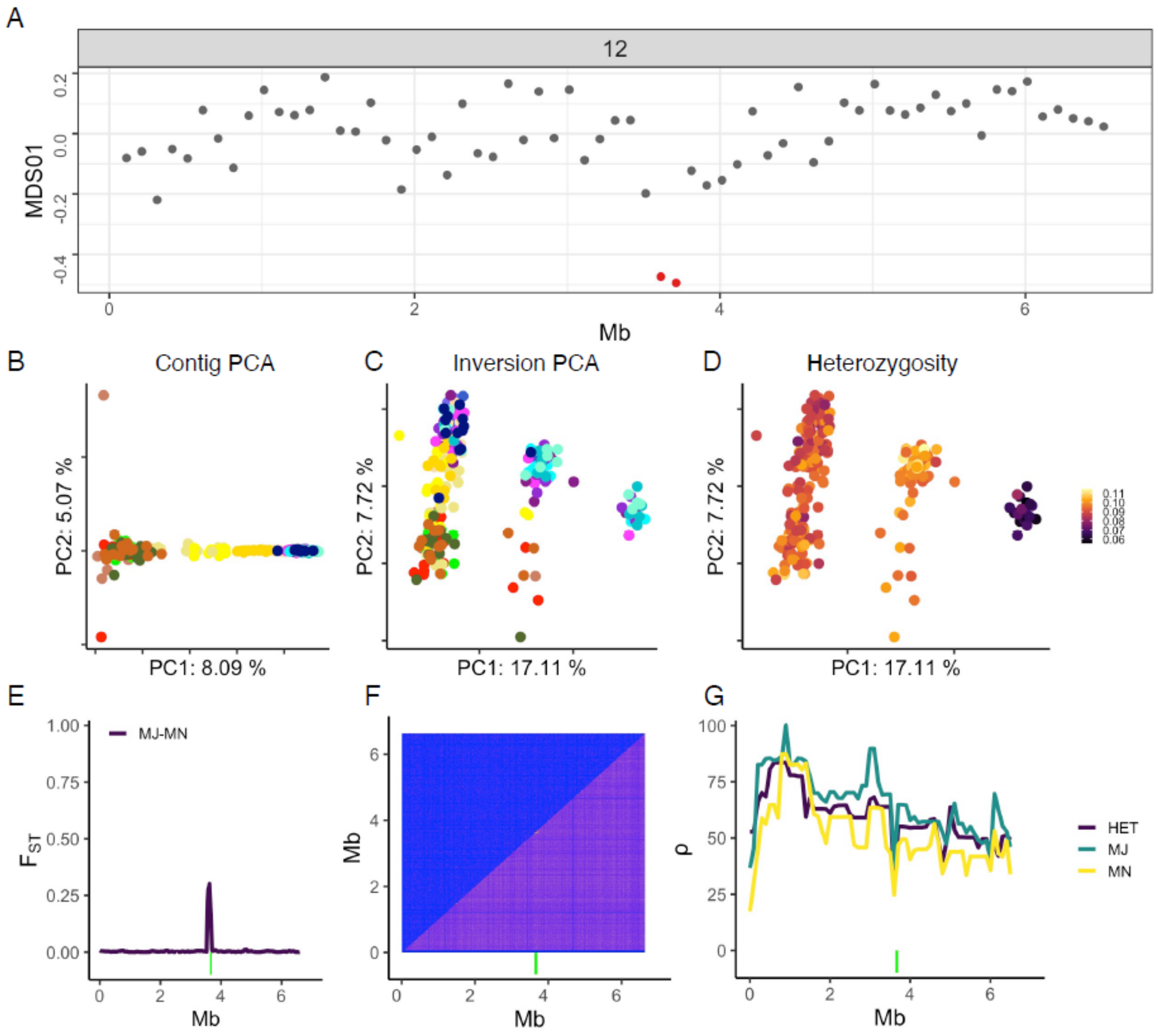

A
12
MDS01
0.2
0.0
-0.2
-0.4
0
2
4
6
Mb
B
Contig PCA
PC2: 5.07 %
PC1: 8.09 %
C
Inversion PCA
PC2: 7.72 %
PC1: 17.11 %
D
Heterozygosity
PC2: 7.72 %
PC1: 17.11 %
E
MJ-MN
1.00
0.75
0.50
0.25
0.00
0
2
4
6
Mb
F
6
4
2
0
Mb
0
2
4
6
Mb
G
100
75
50
25
0
ρ
0
2
4
6
Mb
HET
MJ
MN

Inv13

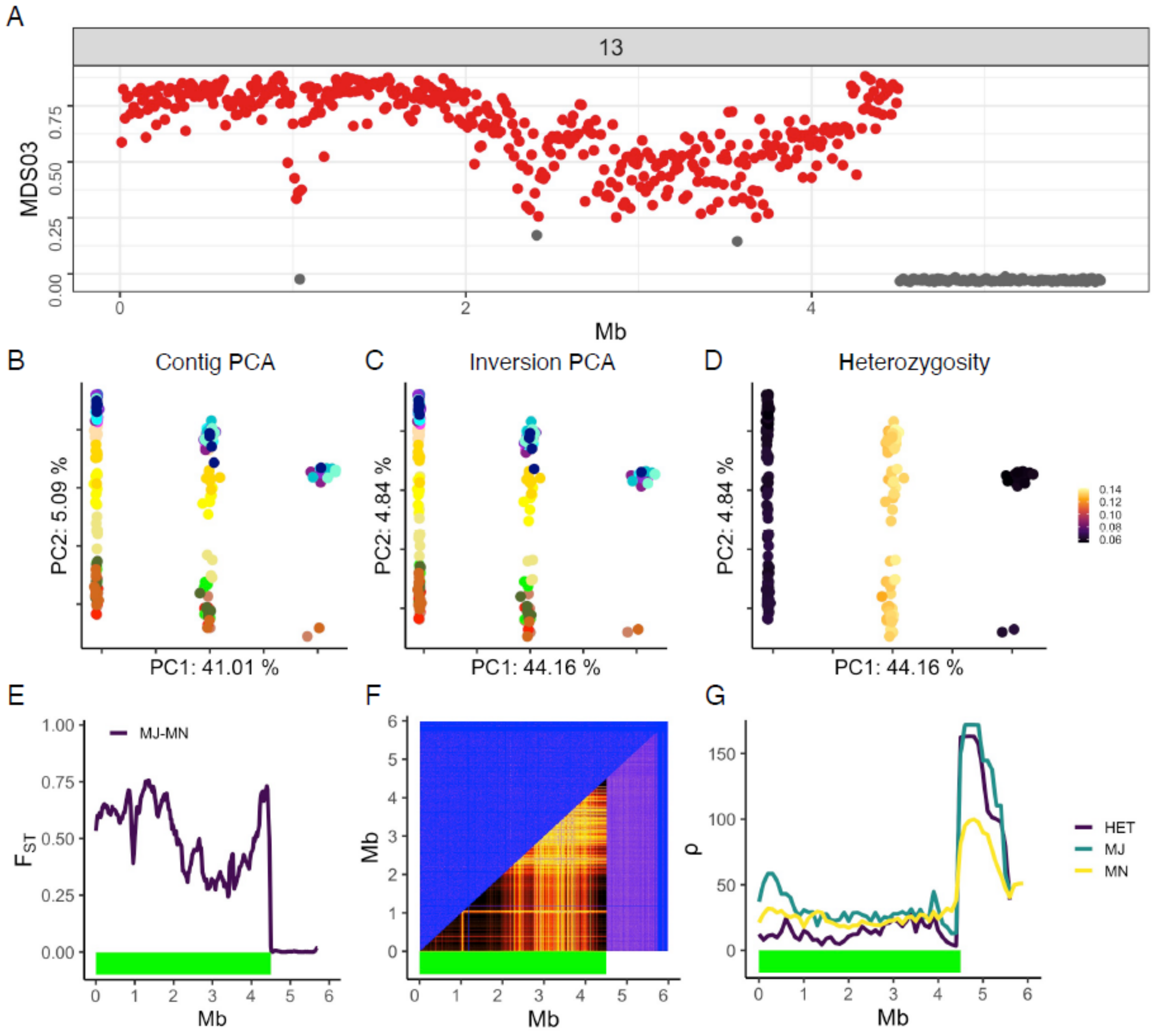

A
13
MDS03
0.00
0.25
0.50
0.75
0
2
4
Mb
B
Contig PCA
PC2: 5.09 %
PC1: 41.01 %
C
Inversion PCA
PC2: 4.84 %
PC1: 44.16 %
D
Heterozygosity
PC2: 4.84 %
PC1: 44.16 %
0.14
0.12
0.10
0.08
0.06
E
MJ-MN
1.00
0.75
0.50
0.25
0.00
F
ST
0 1 2 3 4 5 6
Mb
F
Mb
6 5 4 3 2 1 0
0 1 2 3 4 5 6
Mb
G
150
100
50
0
ρ
0 1 2 3 4 5 6
Mb
HET
MJ
MN

Inv14.1

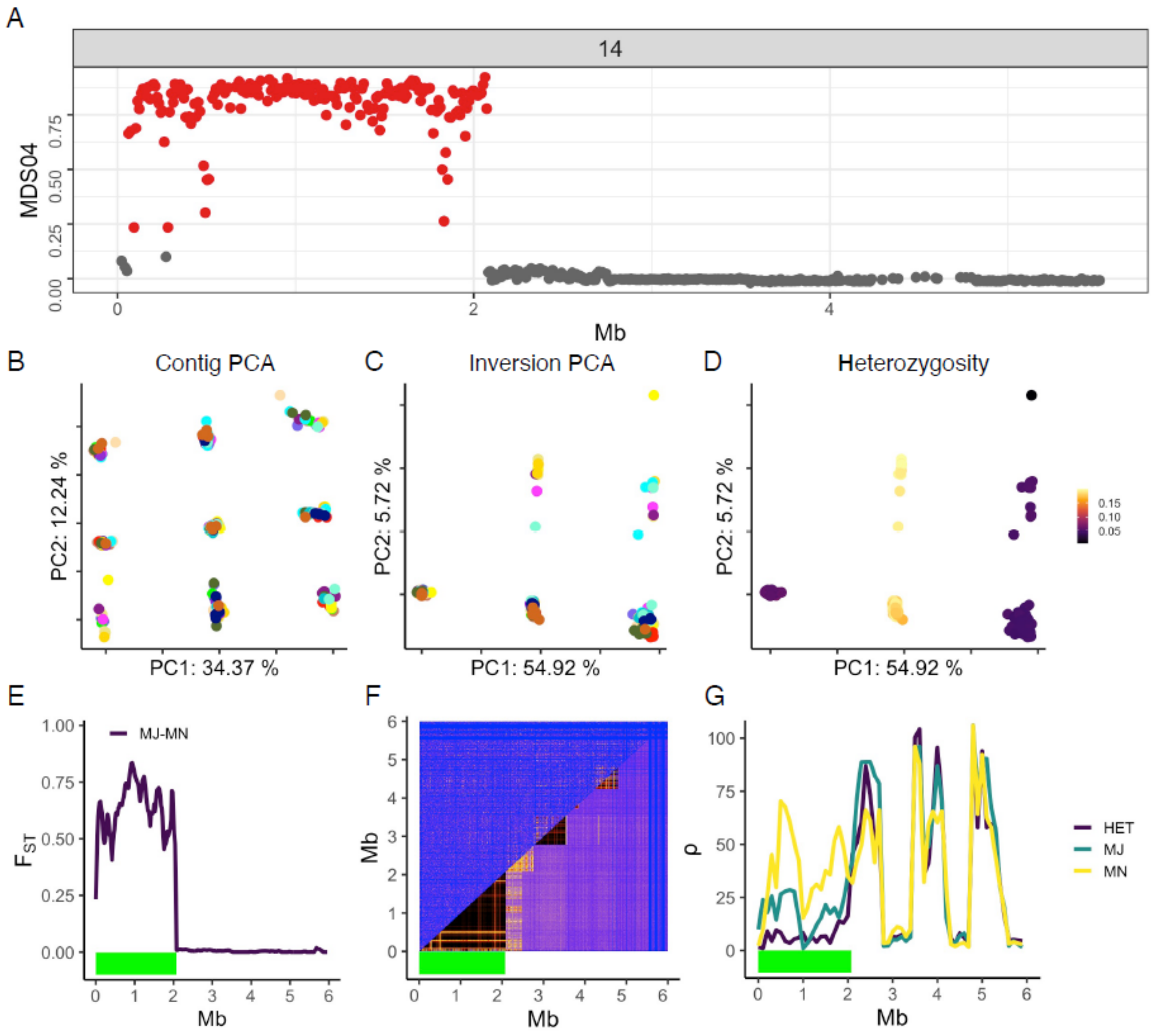

A
14
MDS04
0.00
0.25
0.50
0.75
0
2
4
Mb
B
Contig PCA
PC2: 12.24 %
PC1: 34.37 %
C
Inversion PCA
PC2: 5.72 %
PC1: 54.92 %
D
Heterozygosity
PC2: 5.72 %
PC1: 54.92 %
0.15
0.10
0.05
E
MJ-MN
F_{ST}
1.00
0.75
0.50
0.25
0.00
0
1
2
3
4
5
6
Mb
F
Mb
6
5
4
3
2
1
0
0
1
2
3
4
5
6
Mb
G
ρ
100
75
50
25
0
0
1
2
3
4
5
6
Mb
HET
MJ
MN

Inv14.2

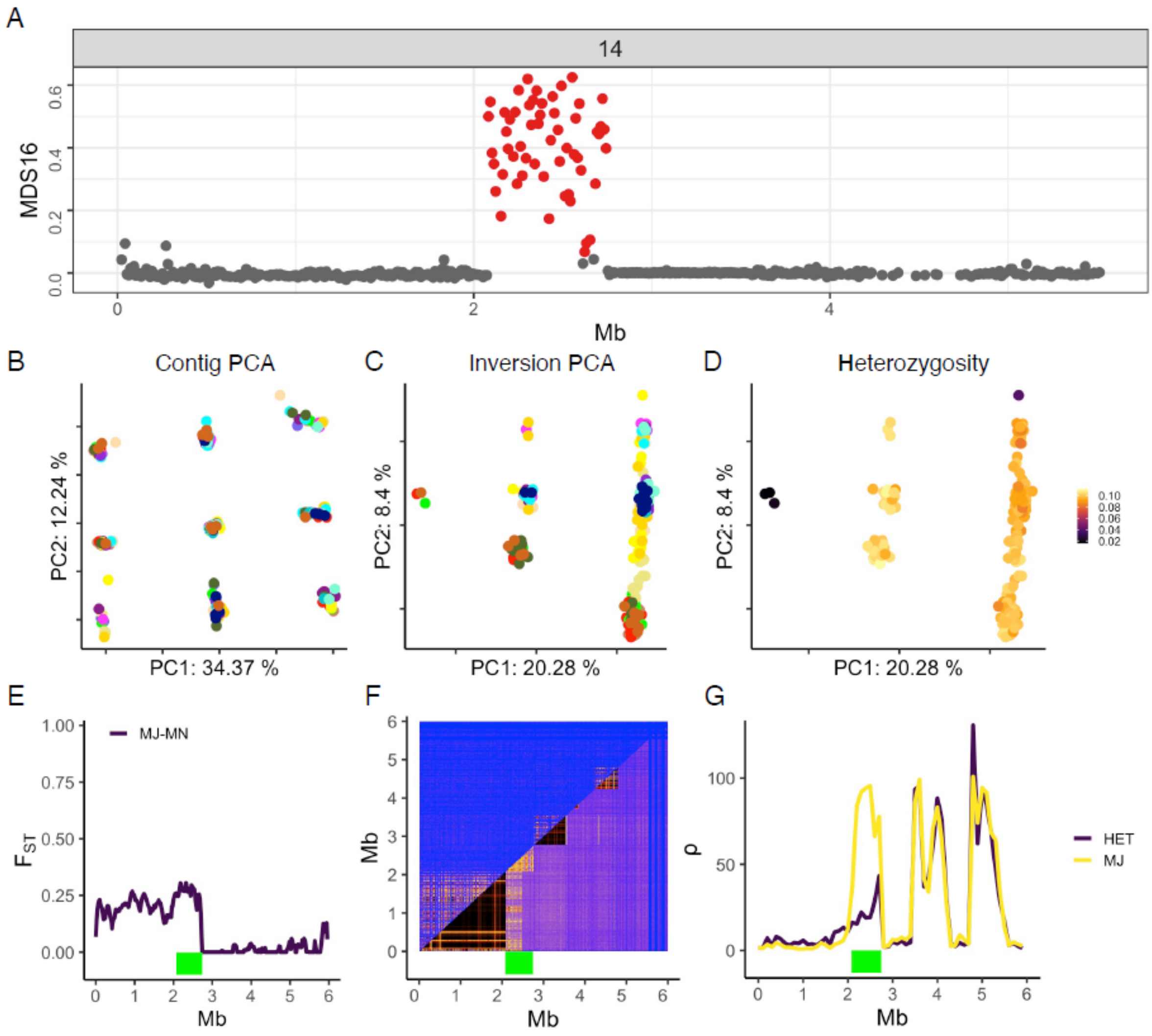
A
14
MDS16
0.0
0.2
0.4
0.6
0
2
4
Mb
B
Contig PCA
PC2: 12.24 %
PC1: 34.37 %
C
Inversion PCA
PC2: 8.4 %
PC1: 20.28 %
D
Heterozygosity
PC2: 8.4 %
PC1: 20.28 %
0.10
0.08
0.06
0.04
0.02
E
MJ-MN
F_{ST}
1.00
0.75
0.50
0.25
0.00
Mb
F
Mb
Mb
G
ρ
100
50
0
HET
MJ
Mb

Inv14.3

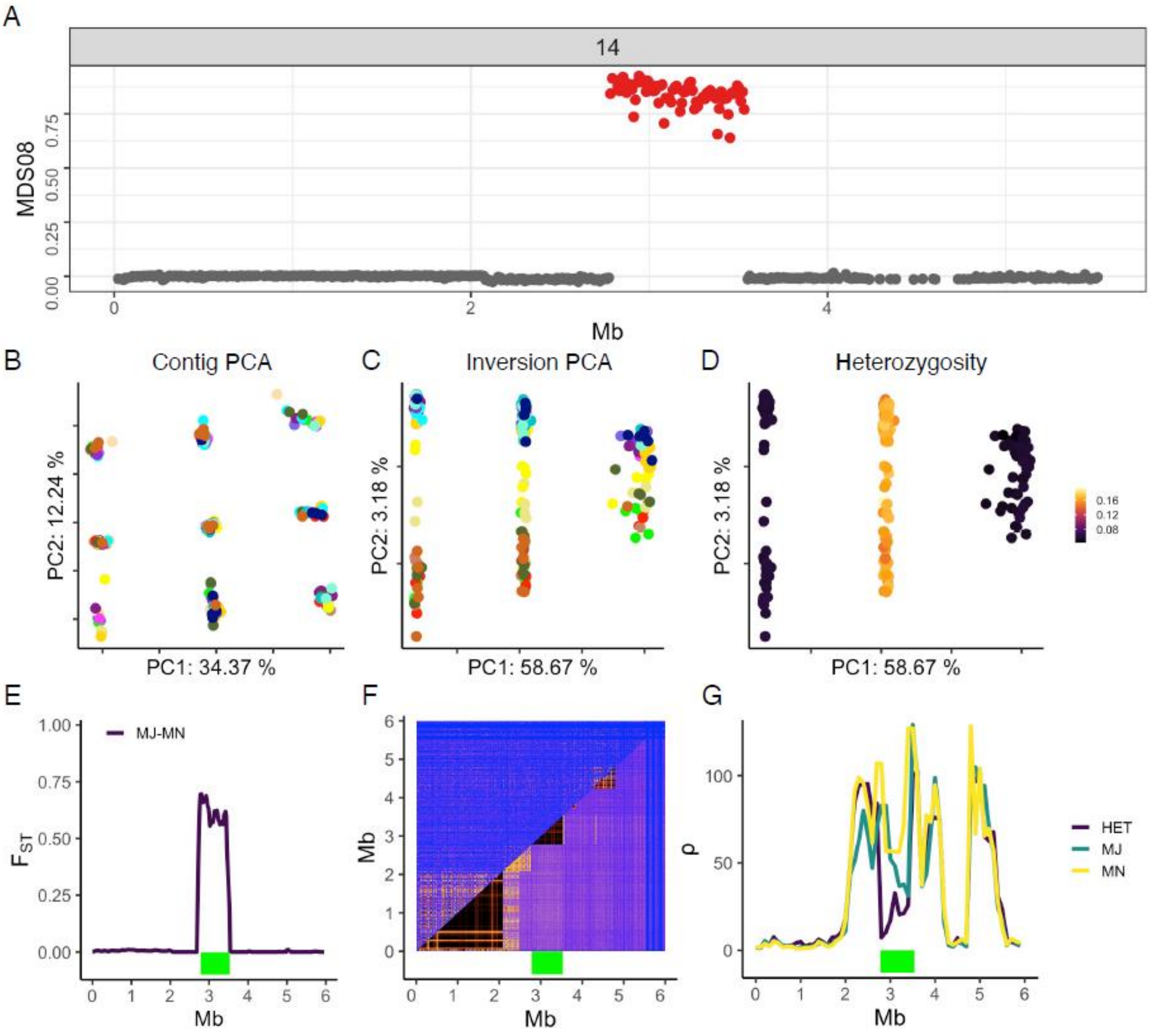
A
14
MDS08
0.00
0.25
0.50
0.75
0
2
4
Mb
B
Contig PCA
PC2: 12.24 %
PC1: 34.37 %
C
Inversion PCA
PC2: 3.18 %
PC1: 58.67 %
D
Heterozygosity
PC2: 3.18 %
PC1: 58.67 %
0.16
0.12
0.08
E
MJ-MN
1.00
0.75
0.50
0.25
0.00
FST
0 1 2 3 4 5 6
Mb
F
Mb
6 5 4 3 2 1 0
0 1 2 3 4 5 6
Mb
G
ρ
100
50
0
0 1 2 3 4 5 6
Mb
HET
MJ
MN

Inv14.4

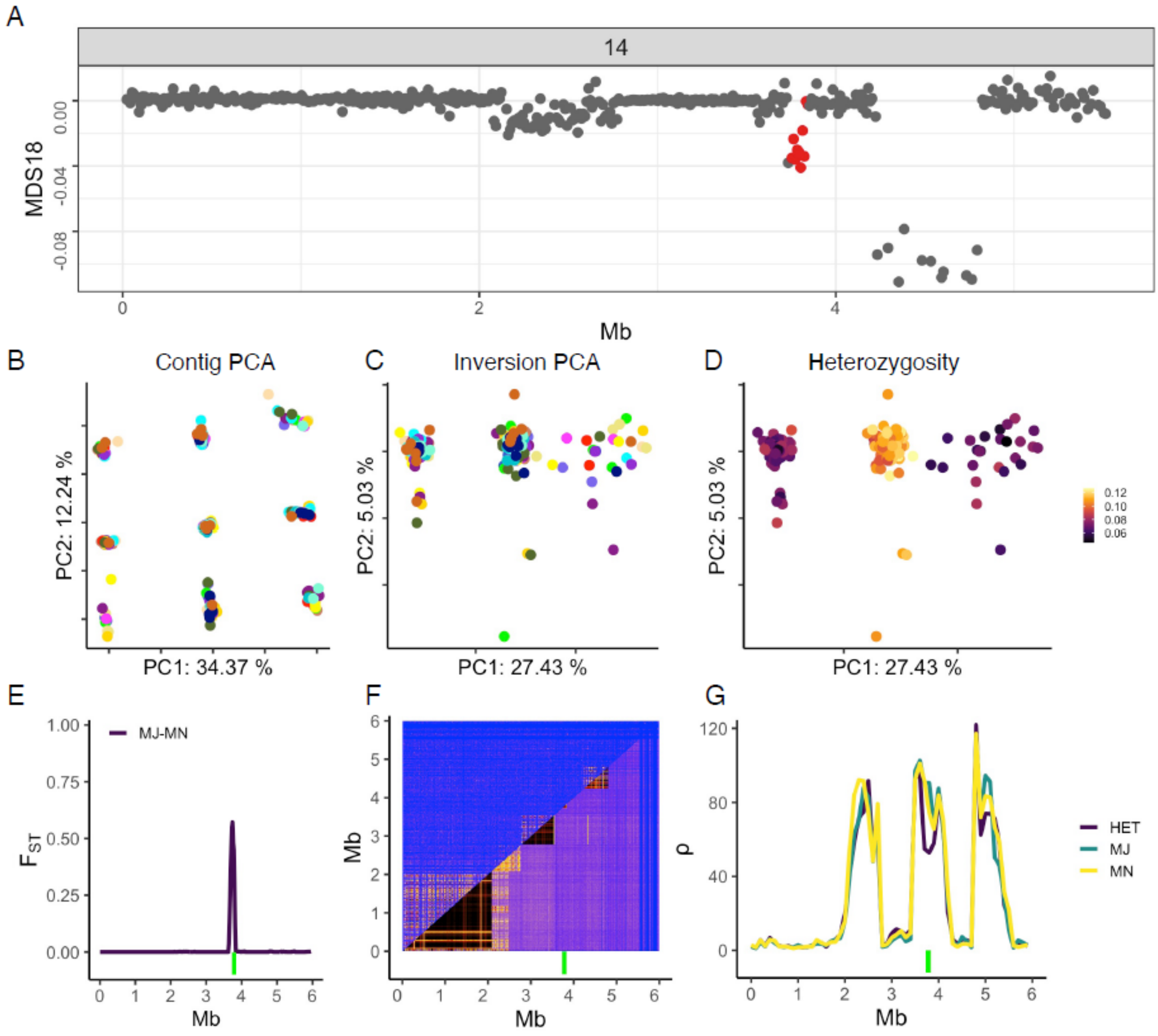

A
14
MDS18
0.00
-0.04
-0.08
0
2
4
Mb
B
Contig PCA
PC2: 12.24 %
PC1: 34.37 %
C
Inversion PCA
PC2: 5.03 %
PC1: 27.43 %
D
Heterozygosity
PC2: 5.03 %
PC1: 27.43 %
0.12
0.10
0.08
0.06
E
MJ-MN
1.00
0.75
0.50
0.25
0.00
Mb
F
Mb
Mb
G
120
80
40
0
ρ
HET
MJ
MN
Mb

Inv14.5

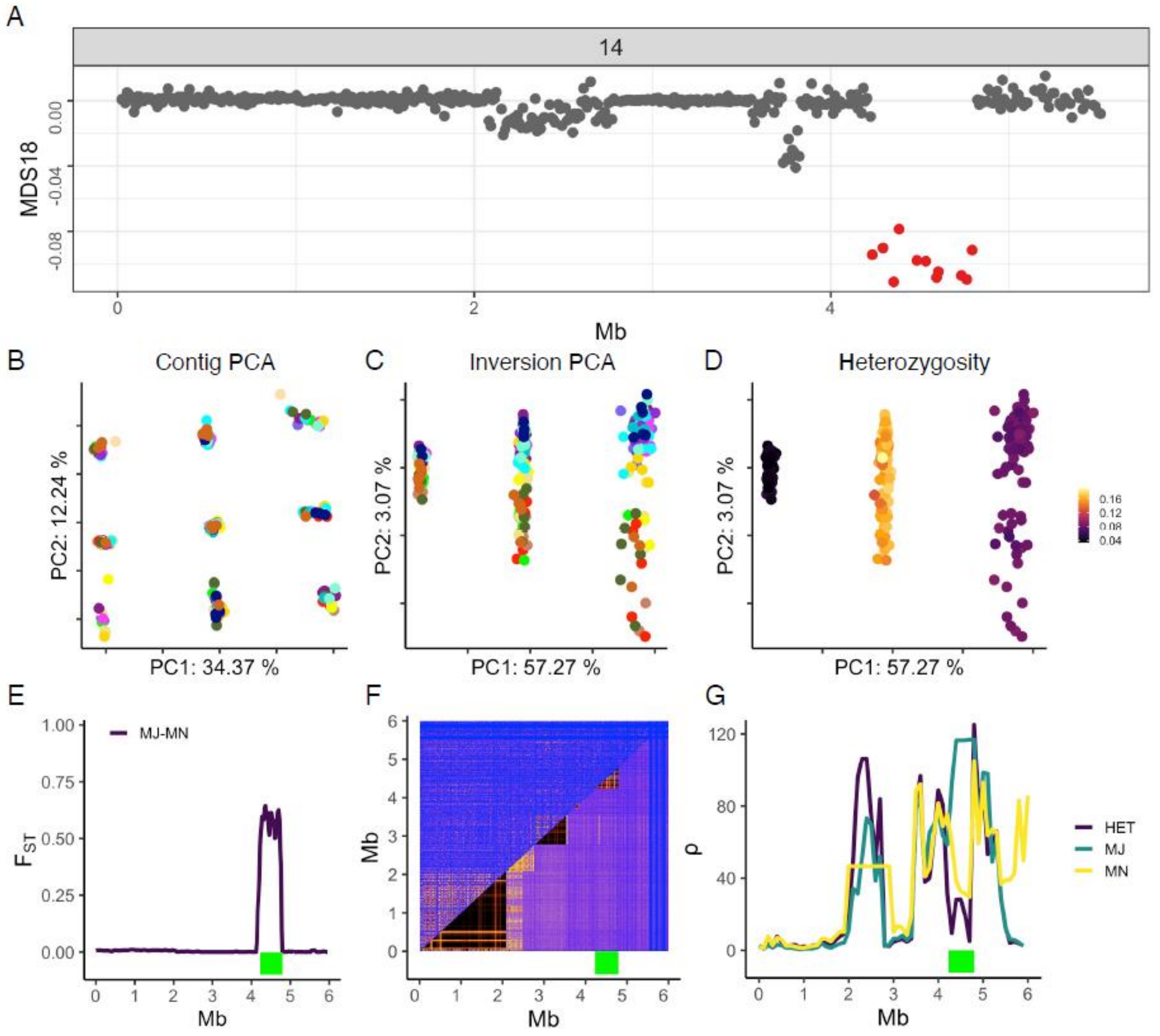

A
14
MDS18
0.00
-0.04
-0.08
0
2
4
Mb
B
Contig PCA
PC2: 12.24 %
PC1: 34.37 %
C
Inversion PCA
PC2: 3.07 %
PC1: 57.27 %
D
Heterozygosity
PC2: 3.07 %
PC1: 57.27 %
0.16
0.12
0.08
0.04
E
MJ-MN
1.00
0.75
0.50
0.25
0.00
F_{ST}
0 1 2 3 4 5 6
Mb
F
Mb
6 5 4 3 2 1 0
0 1 2 3 4 5 6
Mb
G
120
80
40
0
ρ
0 1 2 3 4 5 6
Mb
HET
MJ
MN

Inv15

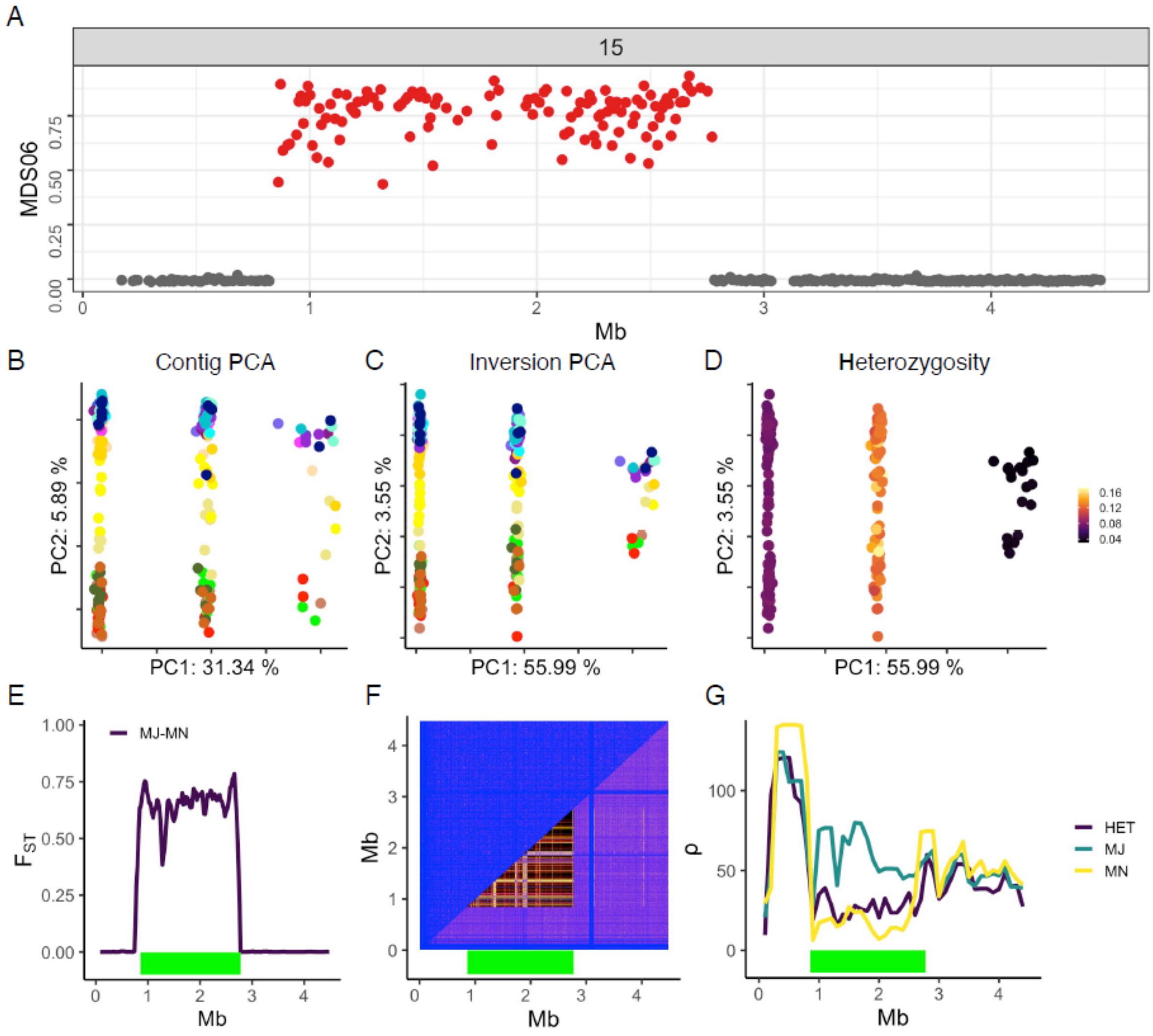

A
15
MDS06
0.00
0.25
0.50
0.75
0
1
2
3
4
Mb
B
Contig PCA
PC2: 5.89 %
PC1: 31.34 %
C
Inversion PCA
PC2: 3.55 %
PC1: 55.99 %
D
Heterozygosity
PC2: 3.55 %
PC1: 55.99 %
0.16
0.12
0.08
0.04
E
MJ-MN
1.00
0.75
0.50
0.25
0.00
F_{ST}
Mb
F
Mb
Mb
G
HET
MJ
MN
100
50
0
ρ
Mb

Inv16.1

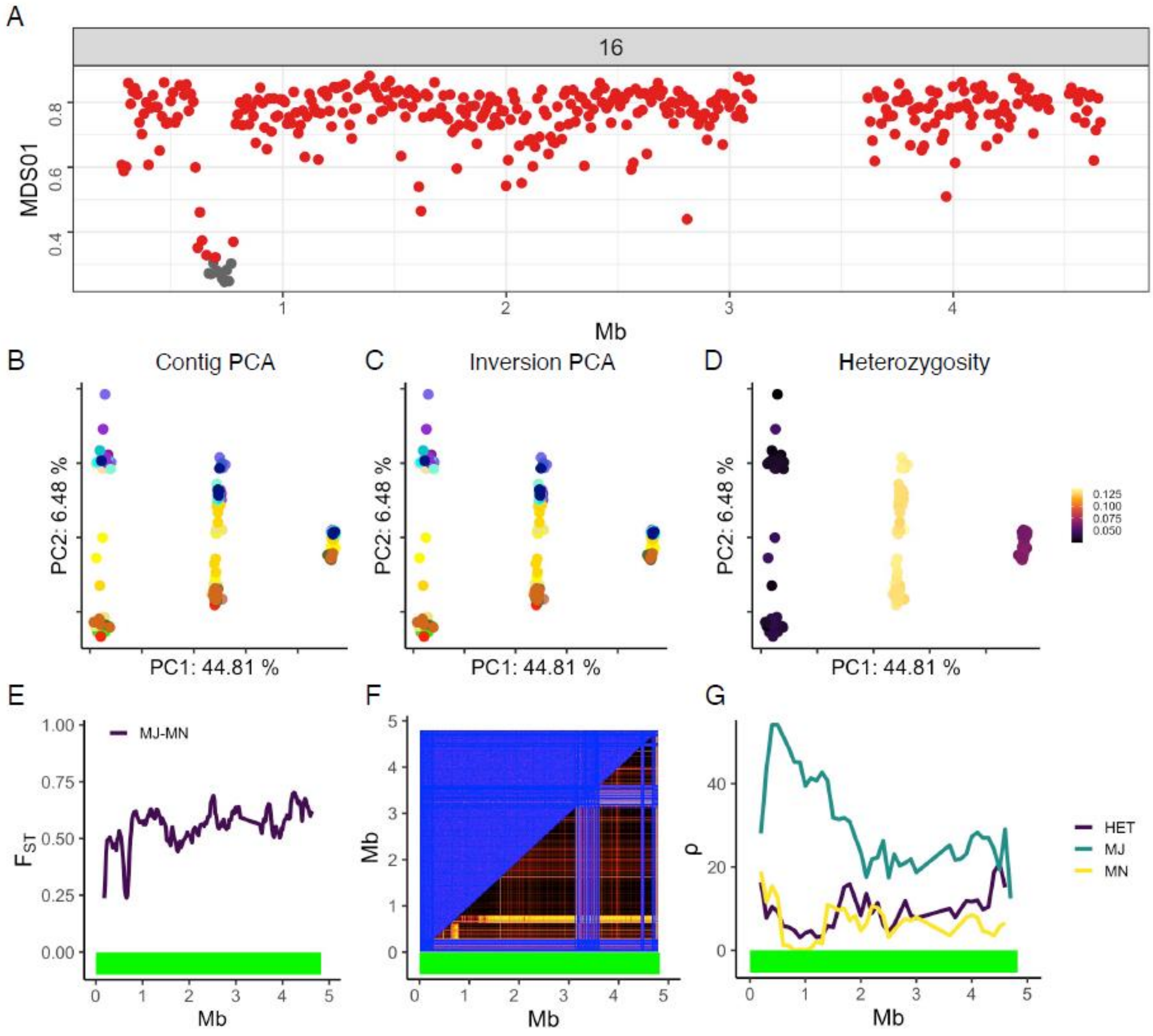

A
16
MDS01
0.4
0.6
0.8
1
2
3
4
Mb
B
Contig PCA
PC2: 6.48 %
PC1: 44.81 %
C
Inversion PCA
PC2: 6.48 %
PC1: 44.81 %
D
Heterozygosity
PC2: 6.48 %
PC1: 44.81 %
0.125
0.100
0.075
0.050
E
MJ-MN
F_{ST}
1.00
0.75
0.50
0.25
0.00
0
1
2
3
4
5
Mb
F
Mb
5
4
3
2
1
0
0
1
2
3
4
5
Mb
G
ρ
40
20
0
0
1
2
3
4
5
Mb
HET
MJ
MN

Inv17

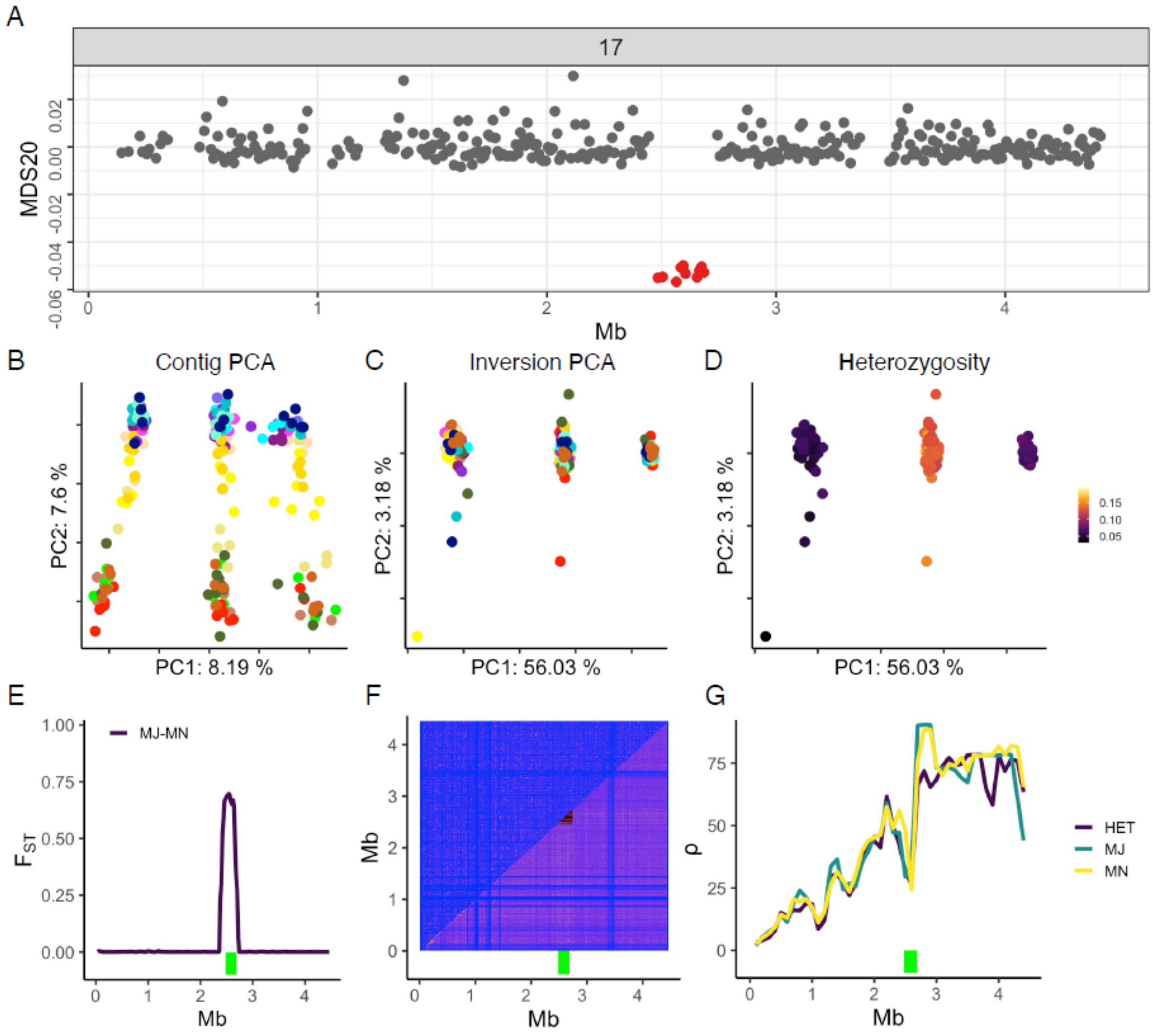

A
17
MDS20
Mb
B
Contig PCA
PC2: 7.6 %
PC1: 8.19 %
C
Inversion PCA
PC2: 3.18 %
PC1: 56.03 %
D
Heterozygosity
PC2: 3.18 %
PC1: 56.03 %
0.15
0.10
0.05
E
MJ-MN
F_{ST}
Mb
F
Mb
Mb
G
ρ
HET
MJ
MN
Mb

Inv18

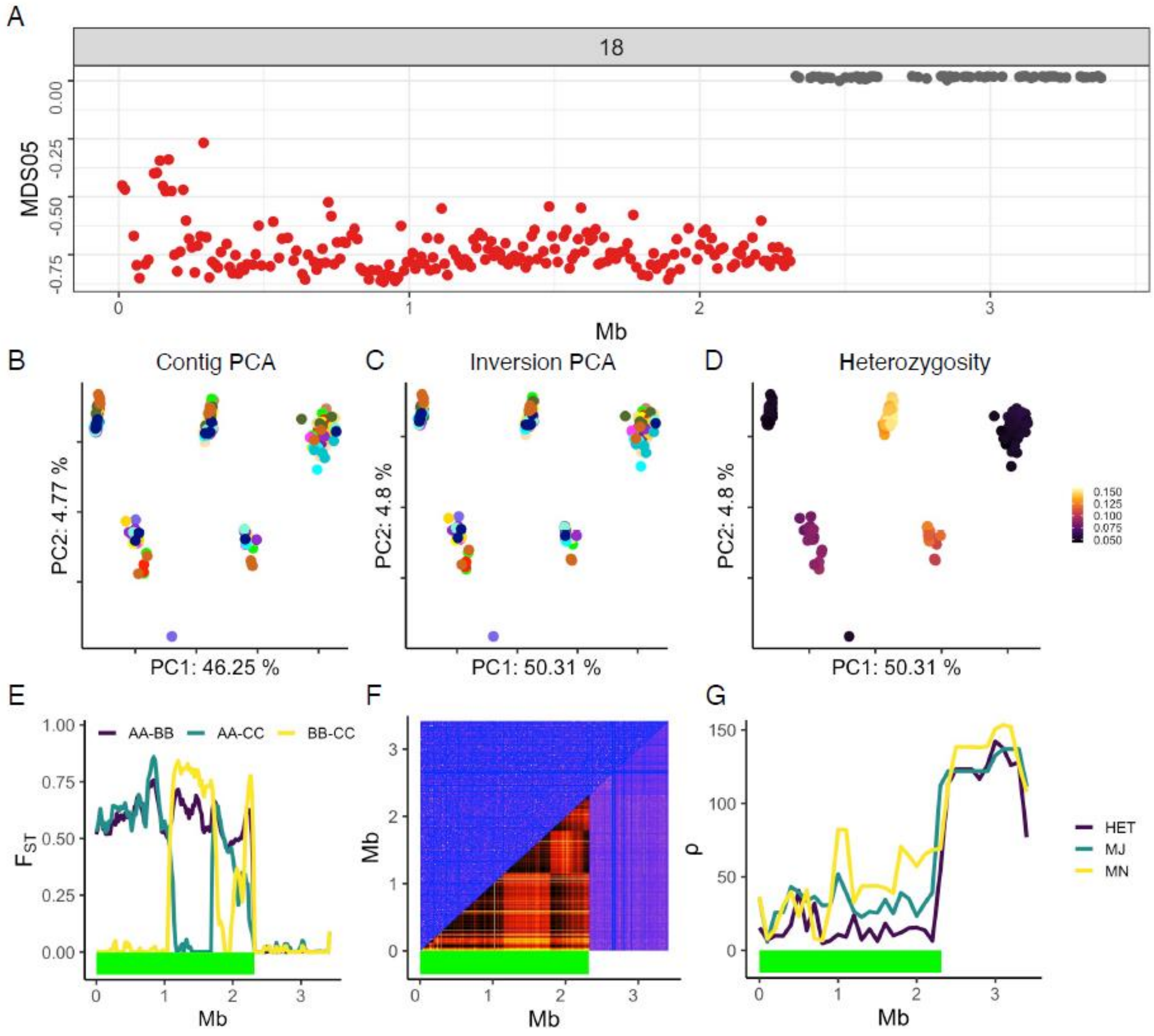

A
18
MDS05
0.00
-0.25
-0.50
-0.75
0
1
2
3
Mb
B
Contig PCA
PC2: 4.77 %
PC1: 46.25 %
C
Inversion PCA
PC2: 4.8 %
PC1: 50.31 %
D
Heterozygosity
PC2: 4.8 %
PC1: 50.31 %
0.150
0.125
0.100
0.075
0.050
E
AA-BB
AA-CC
BB-CC
1.00
0.75
0.50
0.25
0.00
Mb
F
Mb
G
150
100
50
0
HET
MJ
MN
Mb

Inv22.1

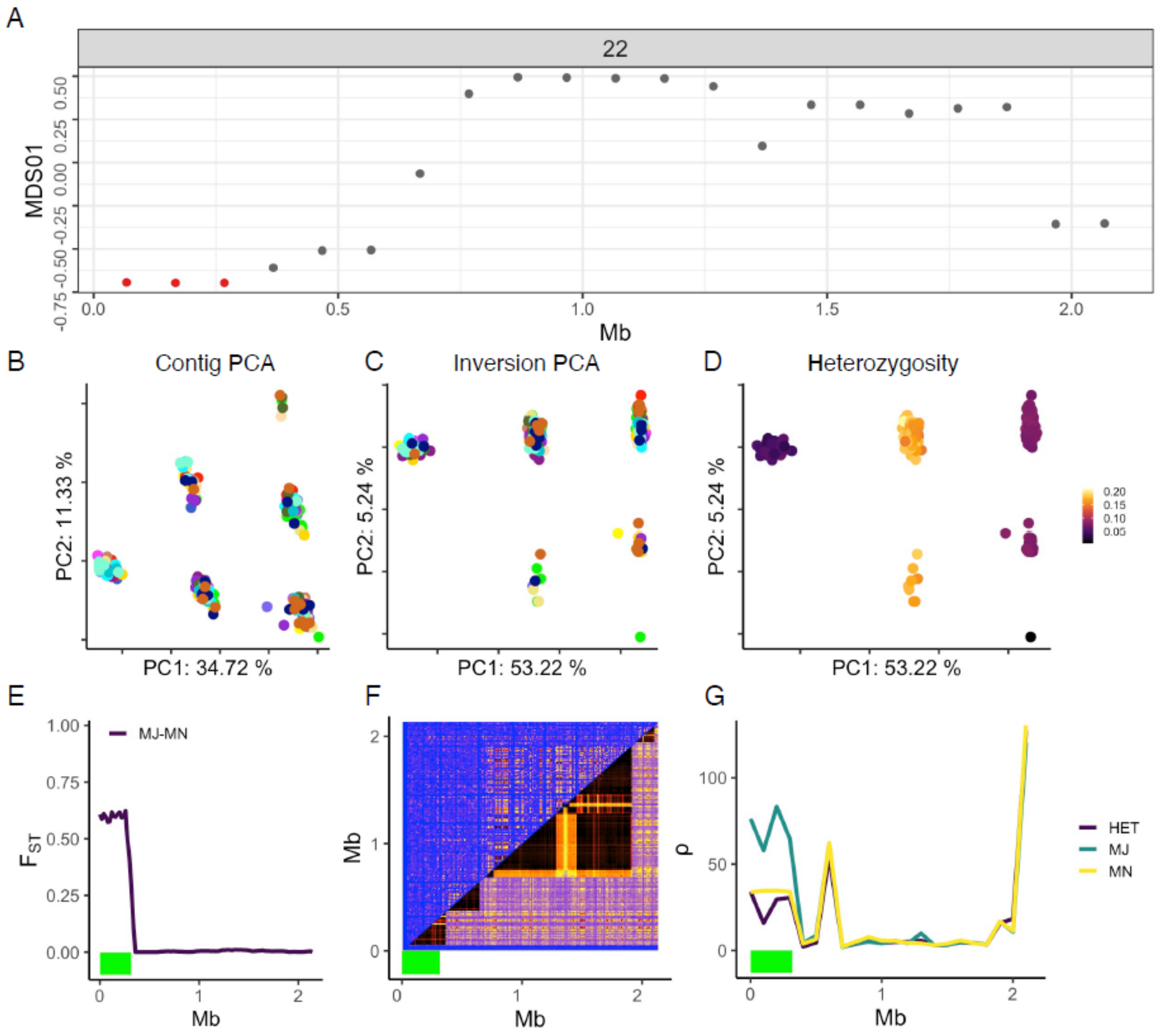
A
22
MDS01
Mb
B
Contig PCA
PC2: 11.33 %
PC1: 34.72 %
C
Inversion PCA
PC2: 5.24 %
PC1: 53.22 %
D
Heterozygosity
PC2: 5.24 %
PC1: 53.22 %
E
MJ-MN
F_{ST}
Mb
F
Mb
Mb
G
ρ
Mb
HET
MJ
MN

Inv22.2

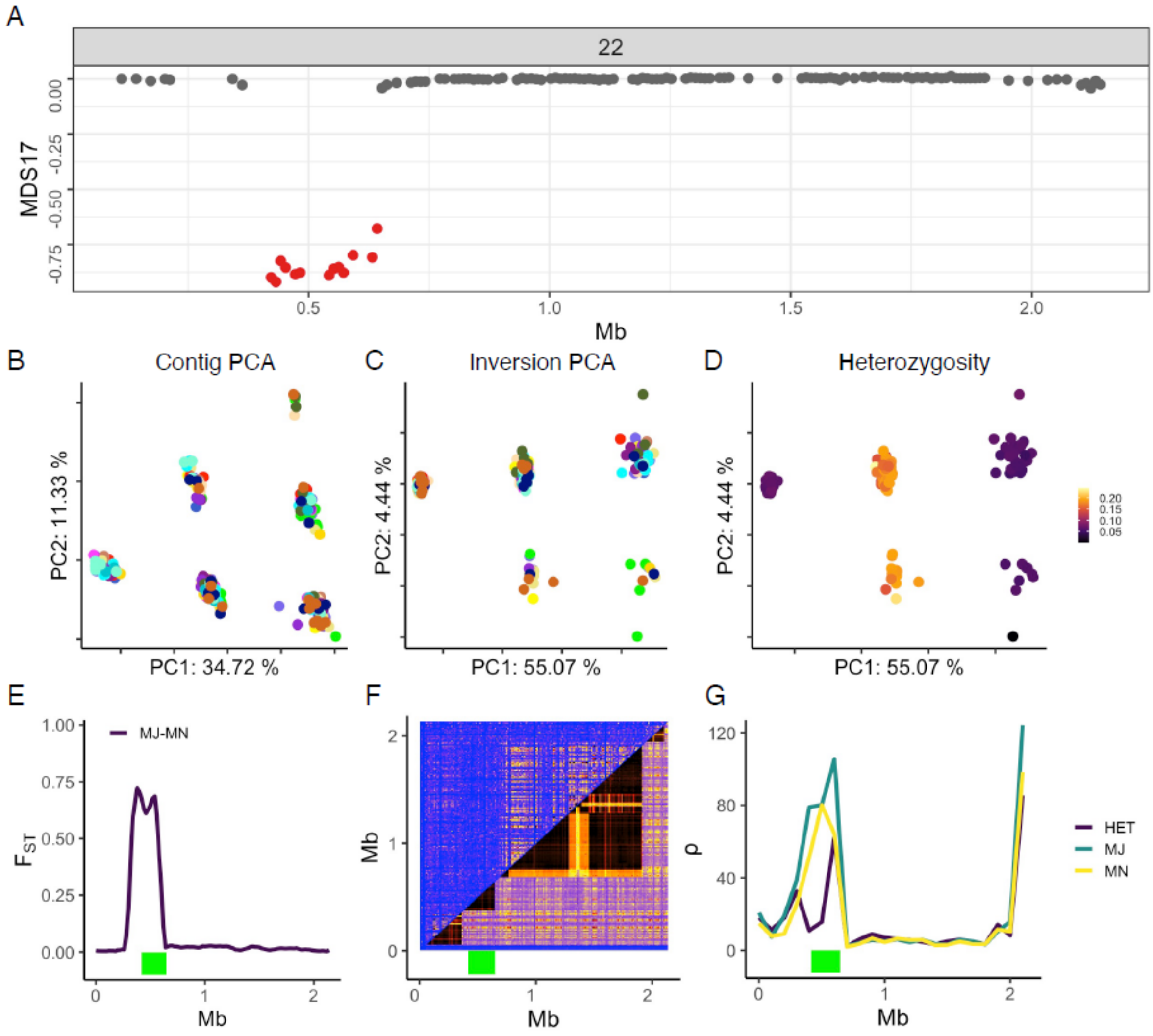

A
22
MDS17
0.00
-0.25
-0.50
-0.75
0.5
1.0
1.5
2.0
Mb
B
Contig PCA
PC2: 11.33 %
PC1: 34.72 %
C
Inversion PCA
PC2: 4.44 %
PC1: 55.07 %
D
Heterozygosity
PC2: 4.44 %
PC1: 55.07 %
0.20
0.15
0.10
0.05
E
1.00
0.75
0.50
0.25
0.00
MJ-MN
F_{ST}
0
1
2
Mb
F
2
1
0
Mb
0
1
2
Mb
G
120
80
40
0
ρ
HET
MJ
MN
0
1
2
Mb

Inv22.3

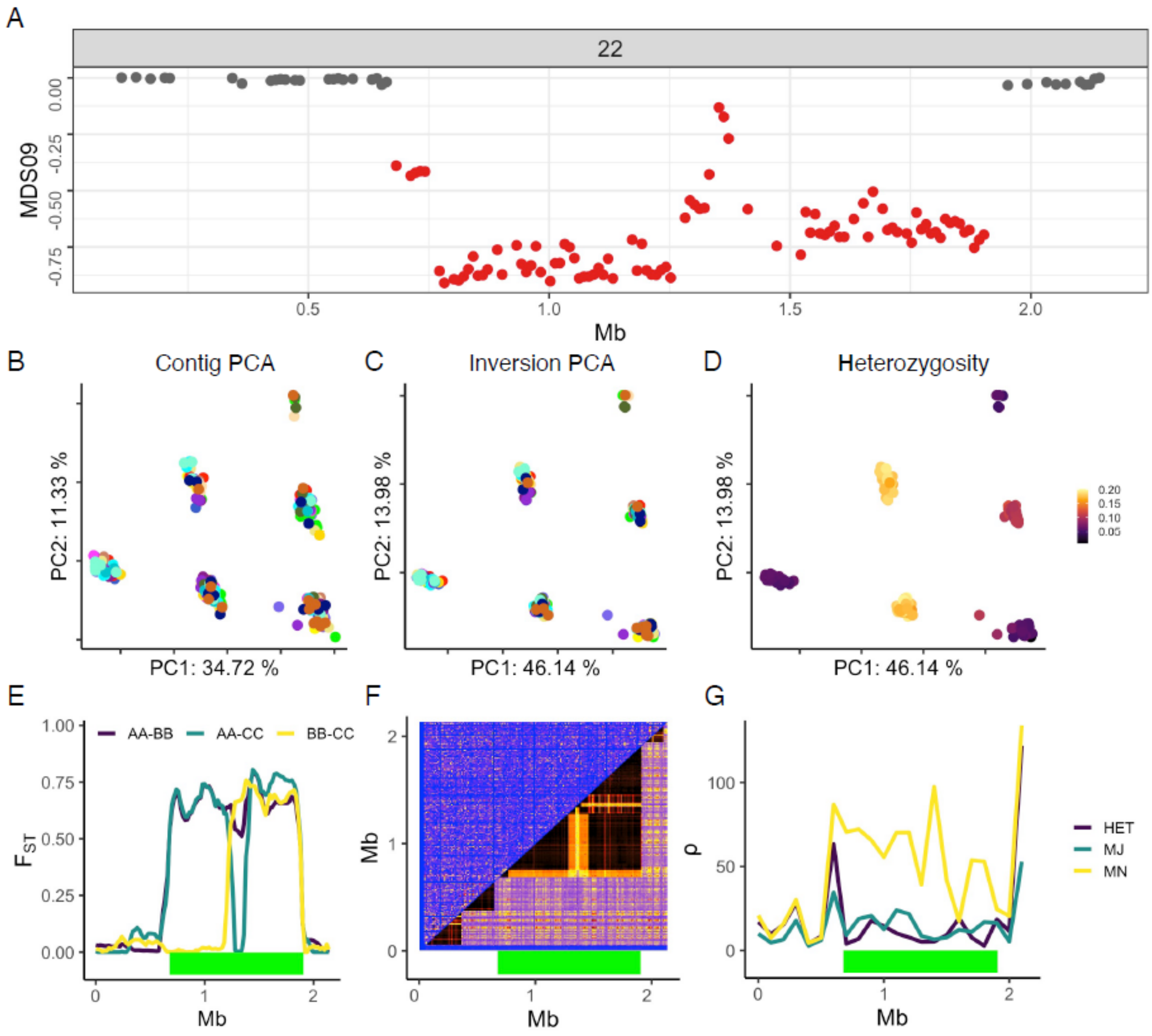
A
22
MDS09
0.00
-0.25
-0.50
-0.75
0.5
1.0
1.5
2.0
Mb
B
Contig PCA
PC2: 11.33 %
PC1: 34.72 %
C
Inversion PCA
PC2: 13.98 %
PC1: 46.14 %
D
Heterozygosity
PC2: 13.98 %
PC1: 46.14 %
0.20
0.15
0.10
0.05
E
AA-BB
AA-CC
BB-CC
1.00
0.75
0.50
0.25
0.00
F_ST
Mb
F
Mb
G
ρ
100
50
0
HET
MJ
MN
Mb

Inv22.4

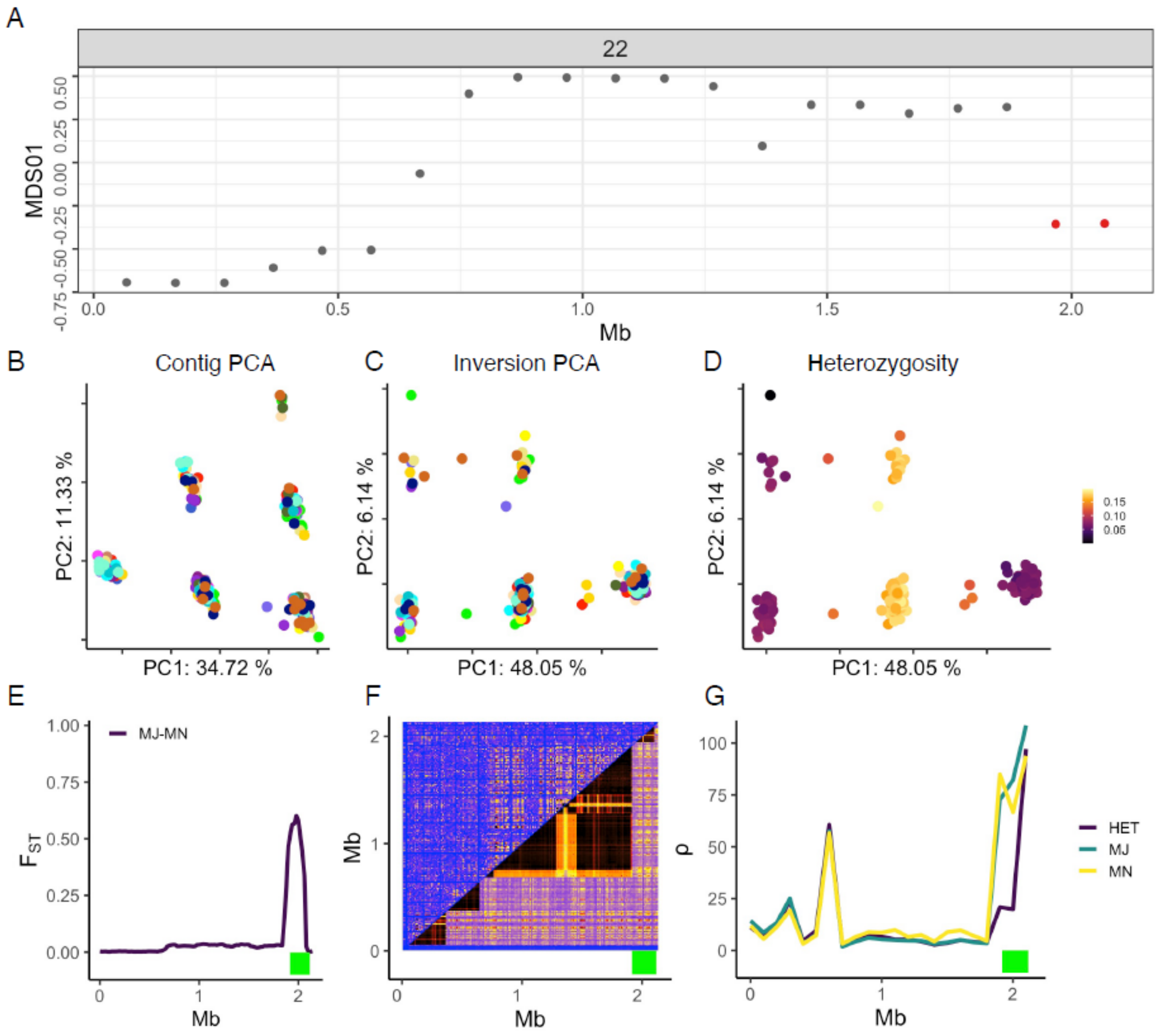

A
22
MDS01
0.50
0.25
0.00
-0.25
-0.50
-0.75
0.0
0.5
1.0
1.5
2.0
Mb
B
Contig PCA
PC2: 11.33 %
PC1: 34.72 %
C
Inversion PCA
PC2: 6.14 %
PC1: 48.05 %
D
Heterozygosity
PC2: 6.14 %
PC1: 48.05 %
0.15
0.10
0.05
E
MJ-MN
F_{ST}
1.00
0.75
0.50
0.25
0.00
0
1
2
Mb
F
Mb
2
1
0
0
1
2
Mb
G
ρ
100
75
50
25
0
0
1
2
Mb
HET
MJ
MN

Inv23.1

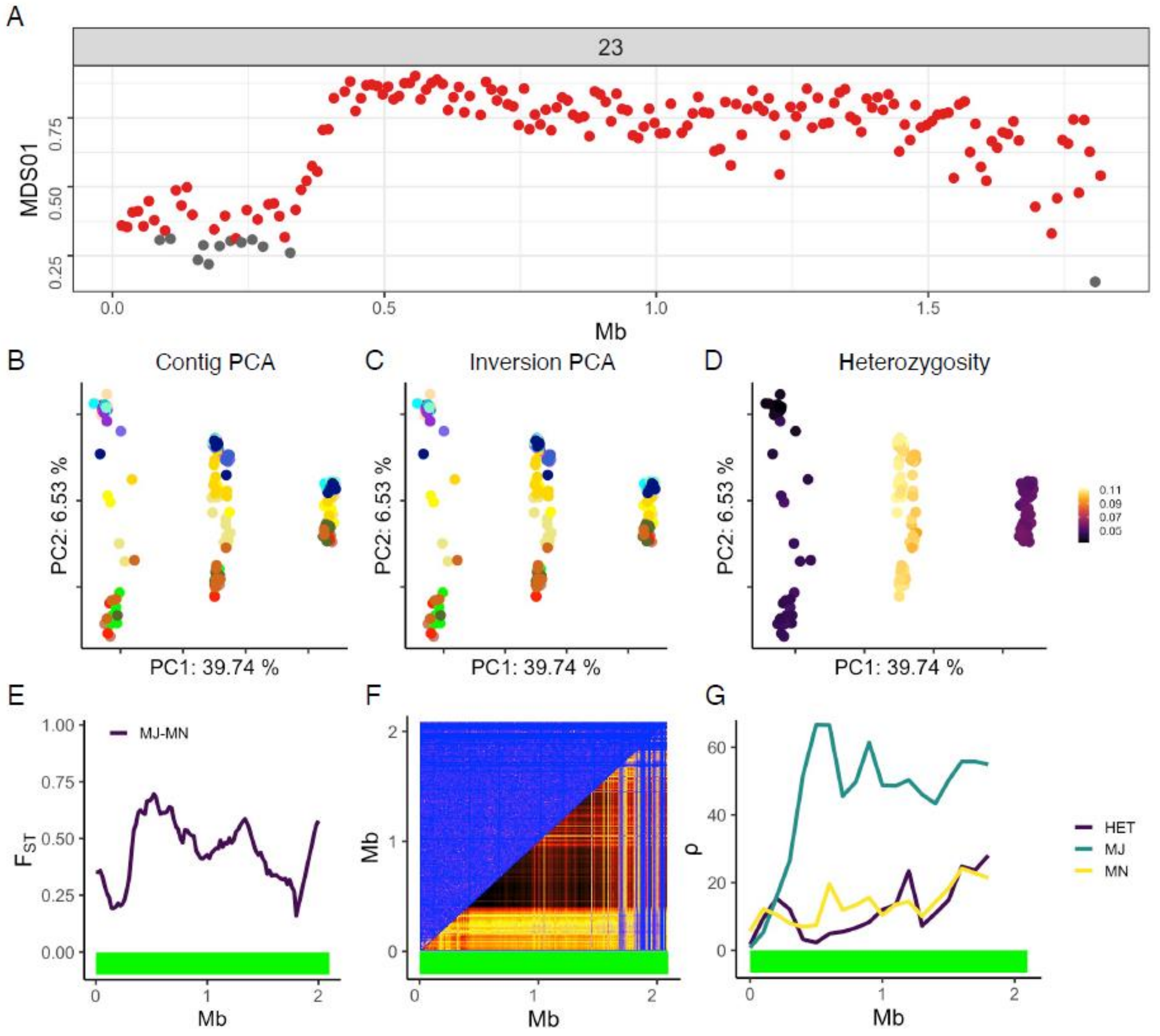

A
23
MDS01
0.25
0.50
0.75
0.0
0.5
1.0
1.5
Mb
B
Contig PCA
PC2: 6.53 %
PC1: 39.74 %
C
Inversion PCA
PC2: 6.53 %
PC1: 39.74 %
D
Heterozygosity
PC2: 6.53 %
PC1: 39.74 %
0.11
0.09
0.07
0.05
E
MJ-MN
1.00
0.75
0.50
0.25
0.00
F
0
1
2
Mb
F
Mb
2
1
0
0
1
2
Mb
G
60
40
20
0
ρ
HET
MJ
MN
0
1
2
Mb

Inv26

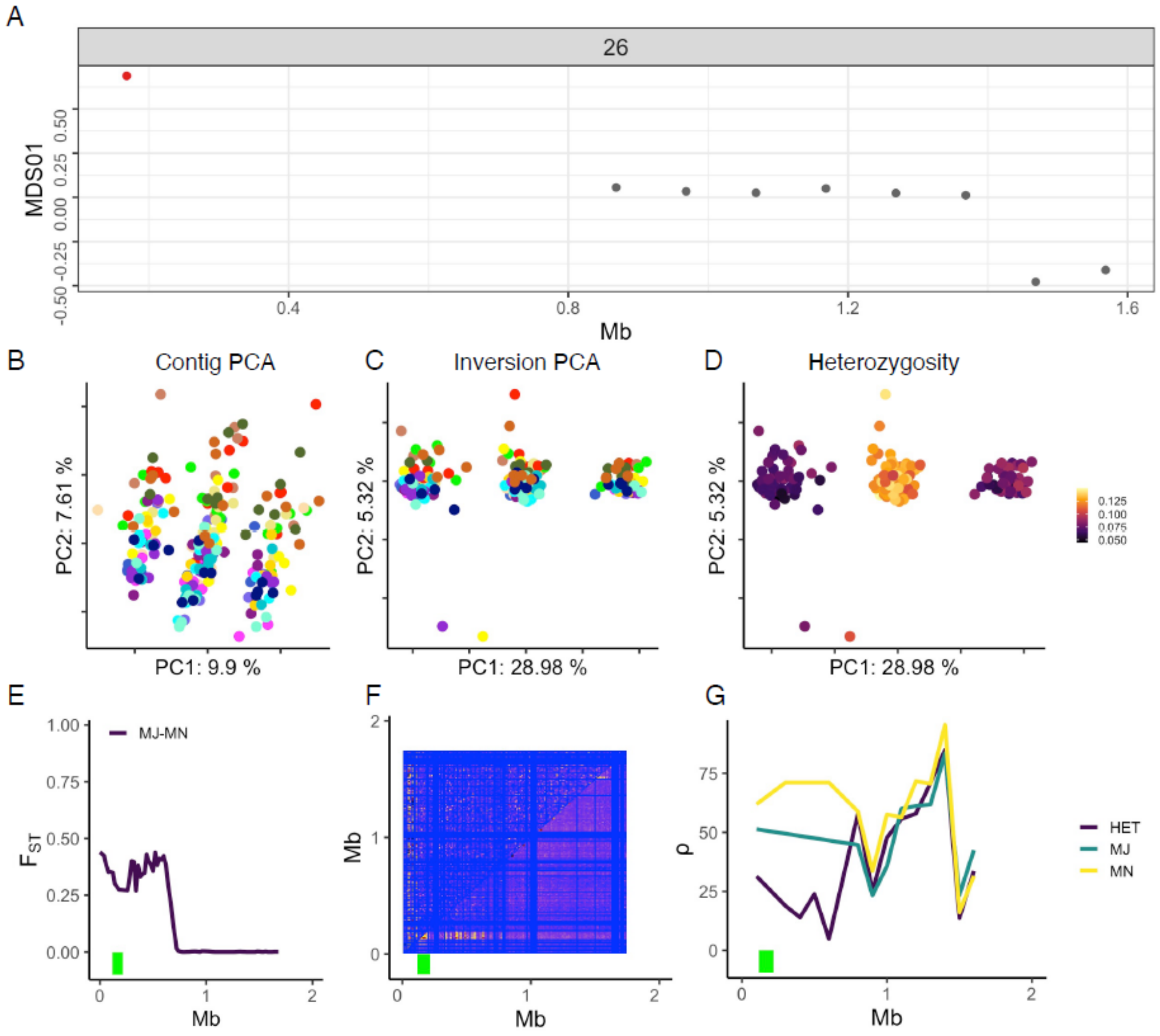

A
26
MDS01
-0.50 -0.25 0.00 0.25 0.50
0.4
0.8
1.2
1.6
Mb
B
Contig PCA
PC2: 7.61 %
PC1: 9.9 %
C
Inversion PCA
PC2: 5.32 %
PC1: 28.98 %
D
Heterozygosity
PC2: 5.32 %
PC1: 28.98 %
0.125
0.100
0.075
0.050
E
MJ-MN
1.00
0.75
0.50
0.25
0.00
FST
0
1
2
Mb
F
Mb
2
1
0
0
1
2
Mb
G
ρ
75
50
25
0
0
1
2
Mb
HET
MJ
MN

Figure S3. Identification of putative double-crossover events and overlapping inversions.

Double-crossover patterns

Plots show linkage disequilibrium (LD) patterns in a contig of interest with a green bar indicating inversion position (A); PCA for inversion region with clusters colored by genotype (B) and heterozygosity (C). Inversion haplotypes are labeled A, B, and C (haplotype created by a putative double crossover event). Double-crossover patterns are characterized by a cross in lighter (lower LD) in the middle of the high LD region and six groups in PCA that correspond to three haplotypes and create six genotypes with heterozygosity patterns that match expectations: homozygotes with lower heterozygosity than heterozygotes.

Inv18

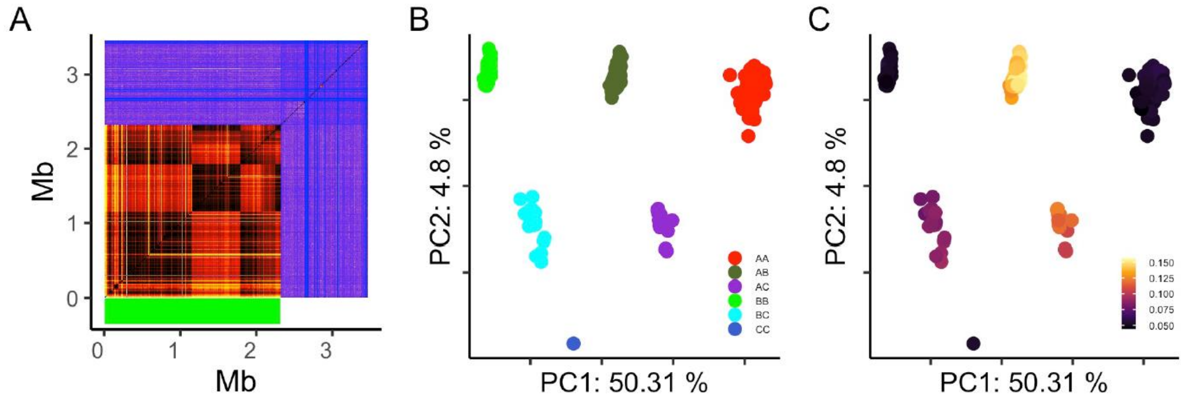

Inv22.3

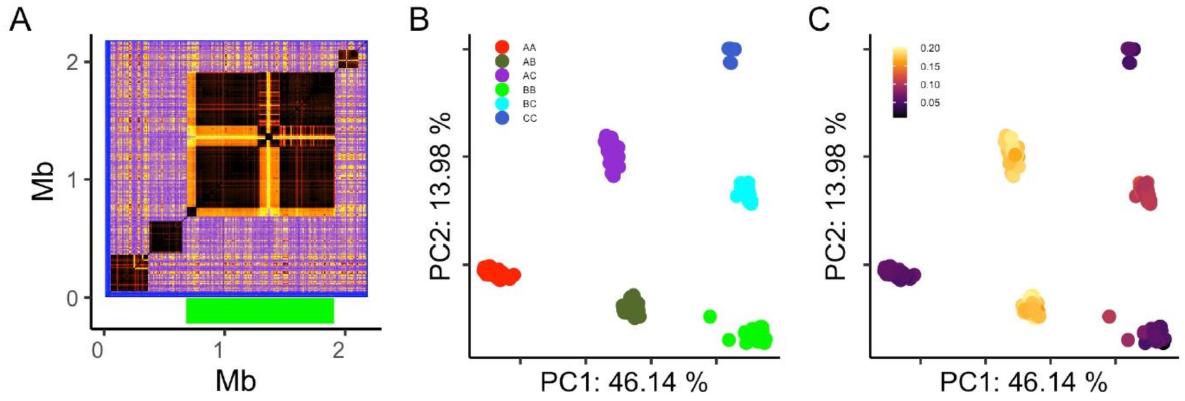

Inv22.4

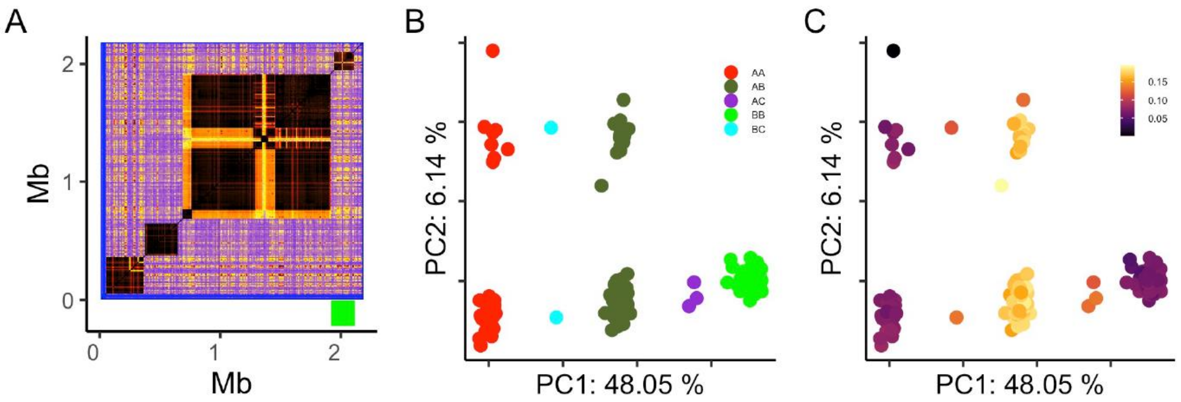

Overlapping inversions

Inv7.2

Inv7.2 overlaps with Inv7.1 (at the beginning of the contig). Plots show linkage disequilibrium (LD) patterns in a contig of interest with a green bar indicating Inv7.2 position (A), PCA for the part of Inv7.2 that overlaps with Inv7.1 (B) and the PCA for the nonoverlapping part of Inv7.2. Both PCAs are colored according to inversion genotype. We genotyped Inv7.2 based on PC2 from (B) and PC1 clustering from (C). Both genotyping methods provided the same results. See FigureS1 for more information on both inversions. AA, AB, BB – different inversion genotypes.

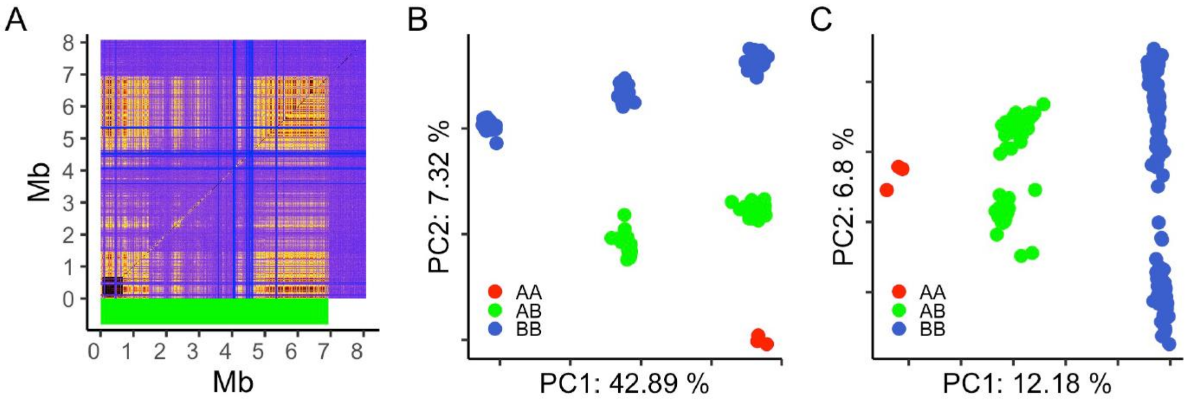

Inv14.6

Inv14.6 overlaps with Inv14.1 and Inv14.2 (at the beginning of the contig). Plots show PCA of the region of Inv14.6 overlapping with Inv14.1 (A); PCA of the region of Inv14.6 overlapping with 14.2 (B), F_{ST} between both Inv14.6 haplotypes (C), linkage disequilibrium patterns for IpsContig14 with Inv14.6 position indicated as green bar (D) and population recombination rate for individuals with different genotypes (E). AA, AB, BB - different inversion genotypes; MJ - more frequent haplotype; MN - less frequent haplotype; HET heterozygotes. Genotyping was performed based on PC2 from (A) and PC2-PCA from (B). Both genotyping methods give the same results.

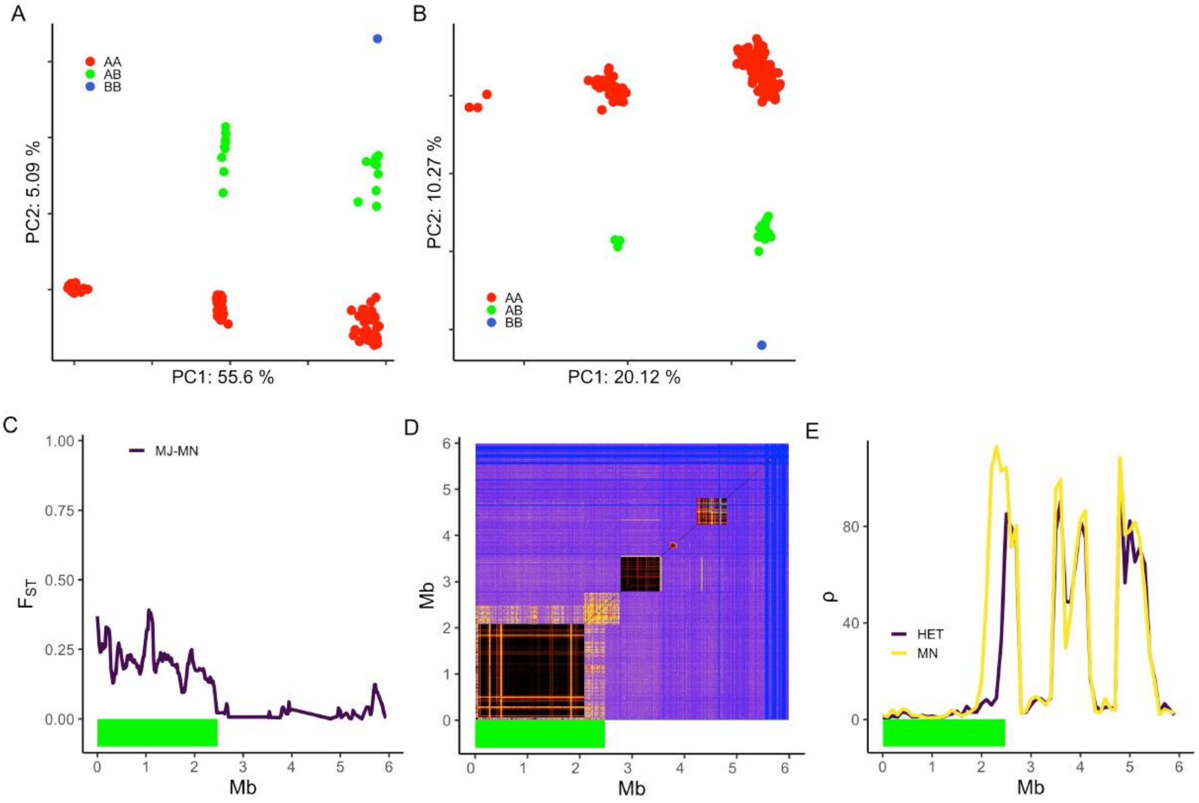

Inv.16.2

Inv16.2 overlaps with Inv16.1. It is not visible at the LD plots (C) but additional clustering to three groups is visible at PC2 from PCA analysis of the whole 16.1 inversion (A). Genotyping was done based on PC2 from PCA from the region of 16.1 (A). B – F_{ST} between both inversion haplotypes; C – linkage disequilibrium patterns; D – population recombination rate for individuals with different genotypes. AA, AB, BB – different inversion genotypes; MJ – more frequent haplotype; MN – less frequent haplotype; HET – heterozygotes. Inversion position indicated with green bar.

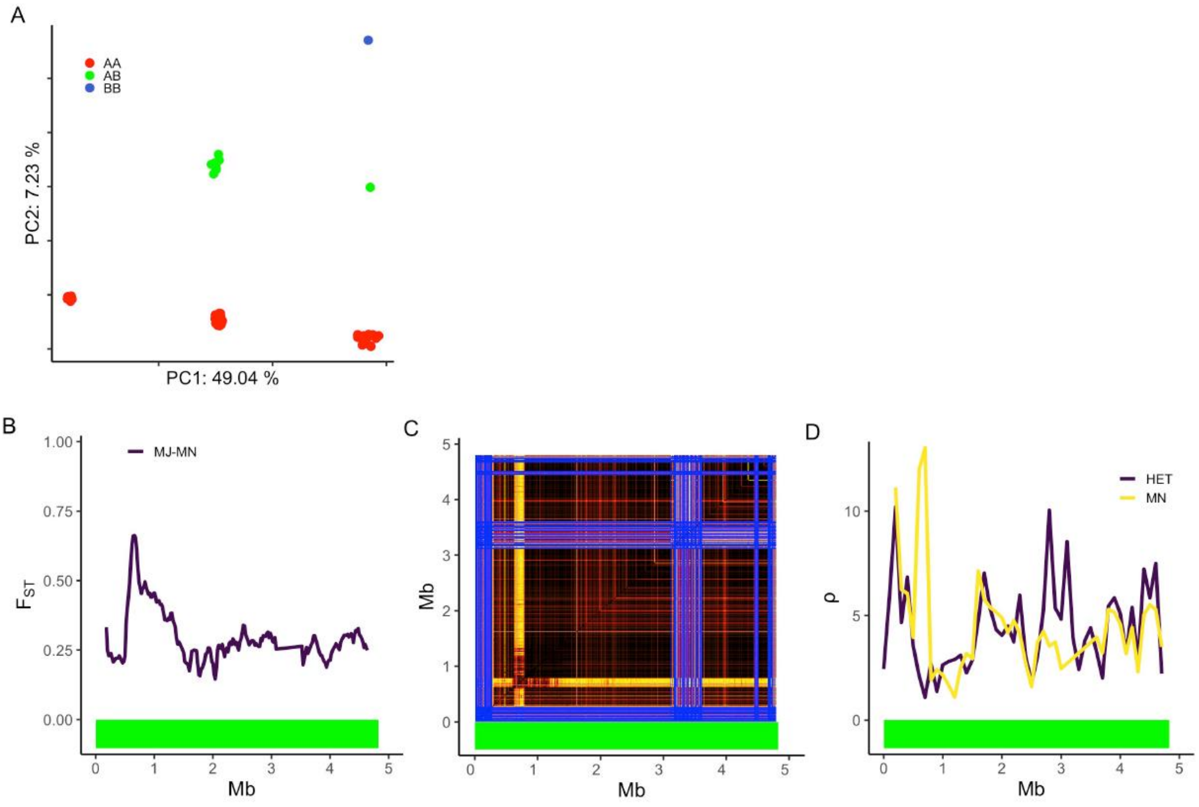

Inv22.5

Inv22.5 overlaps with Inv22.1, Inv22.2, Inv22.3 and 22.4. Genotyping was performed based on PC2 from PCAs of the region of 22.5 overlapping with 22.1 (A); 22.2 (B); 22.4 (D) and PC3 from PCA of the region of 22.5 overlapping with 14.3 (C; in this case PC2 mainly showed patterns of double crossover, see above). All genotyping methods give the same results. E - F_{ST} between the two inversion haplotypes; F - Linkage disequilibrium patterns; G - Population recombination rate for individuals with different genotypes. AA, AB, BB - different inversion genotypes; MJ - more frequent haplotype; MN - less frequent haplotype; HET heterozygotes. The position of the inversion is indicated by a green bar.

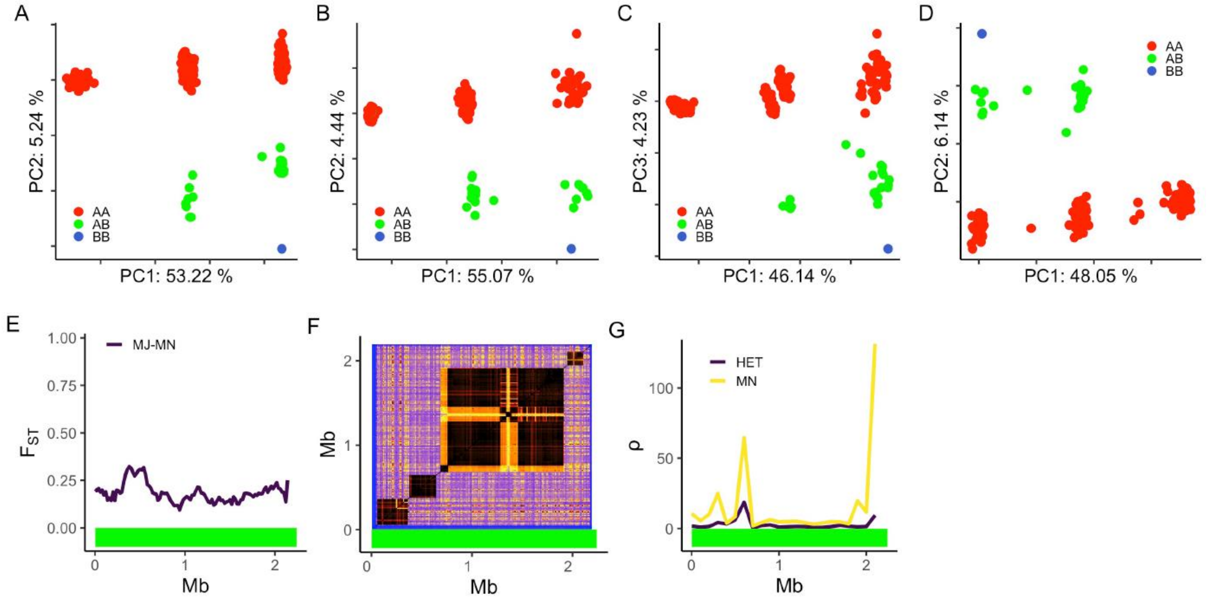

Inv 23.2

Inv23.2 overlaps with Inv23.1. It is not visible in the LD plots (C), but additional clustering into three groups is visible in PC2 from PCA analysis of the entire 23.1 inversion (A). Genotyping was performed based on PC2 from PCA of the 23.1 region (A). B - F_{ST} between both inversion haplotypes; C - Linkage disequilibrium patterns; D - Population recombination rate for individuals with different genotypes. AA, AB, BB - different inversion genotypes; MJ - more frequent haplotype; MN - less frequent haplotype; HET heterozygotes. The position of the inversion is indicated by a green bar.

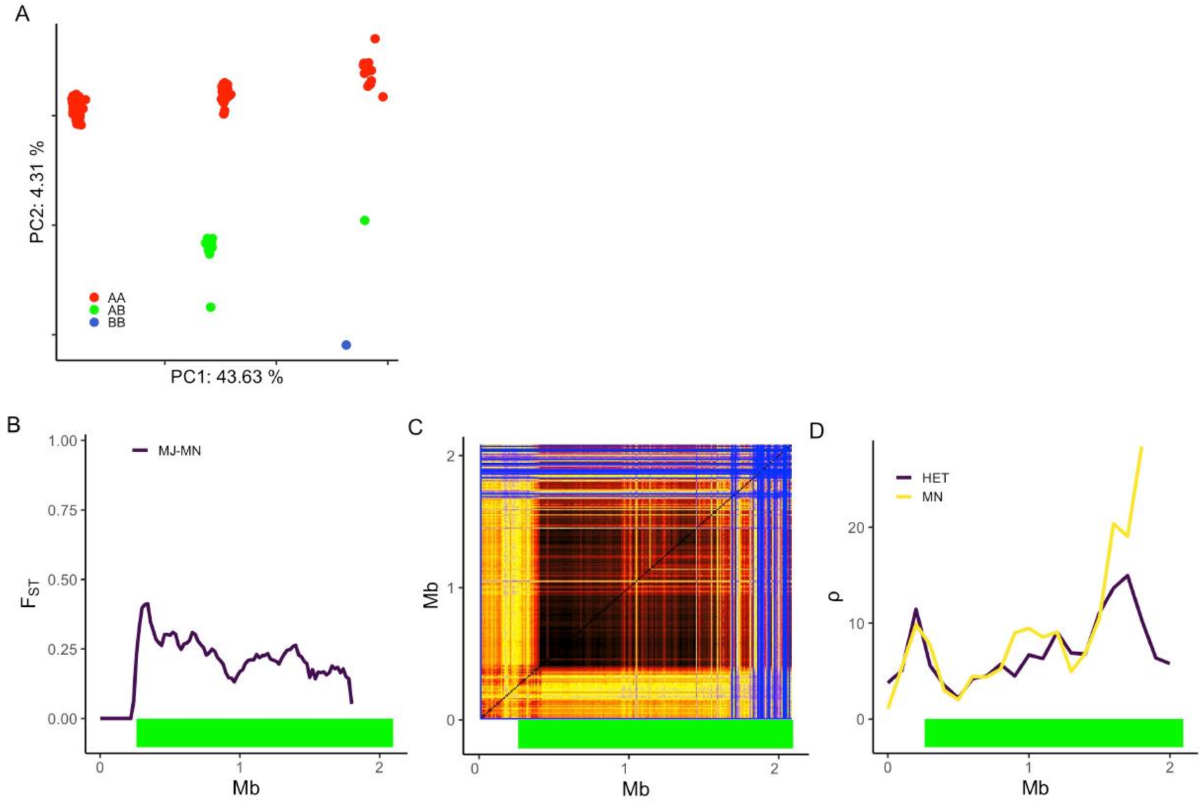

Figure S4 Linkage disequilibrium (LD) between inversions in the *Ips typographus* genome. Non-blue off diagonal squares for Inv16.1 and Inv23.1 indicate parts of the same inversion in different contigs. LD was calculated between inversions that could be genotyped in all studied populations. The color bar represents LD from 0 to 1.

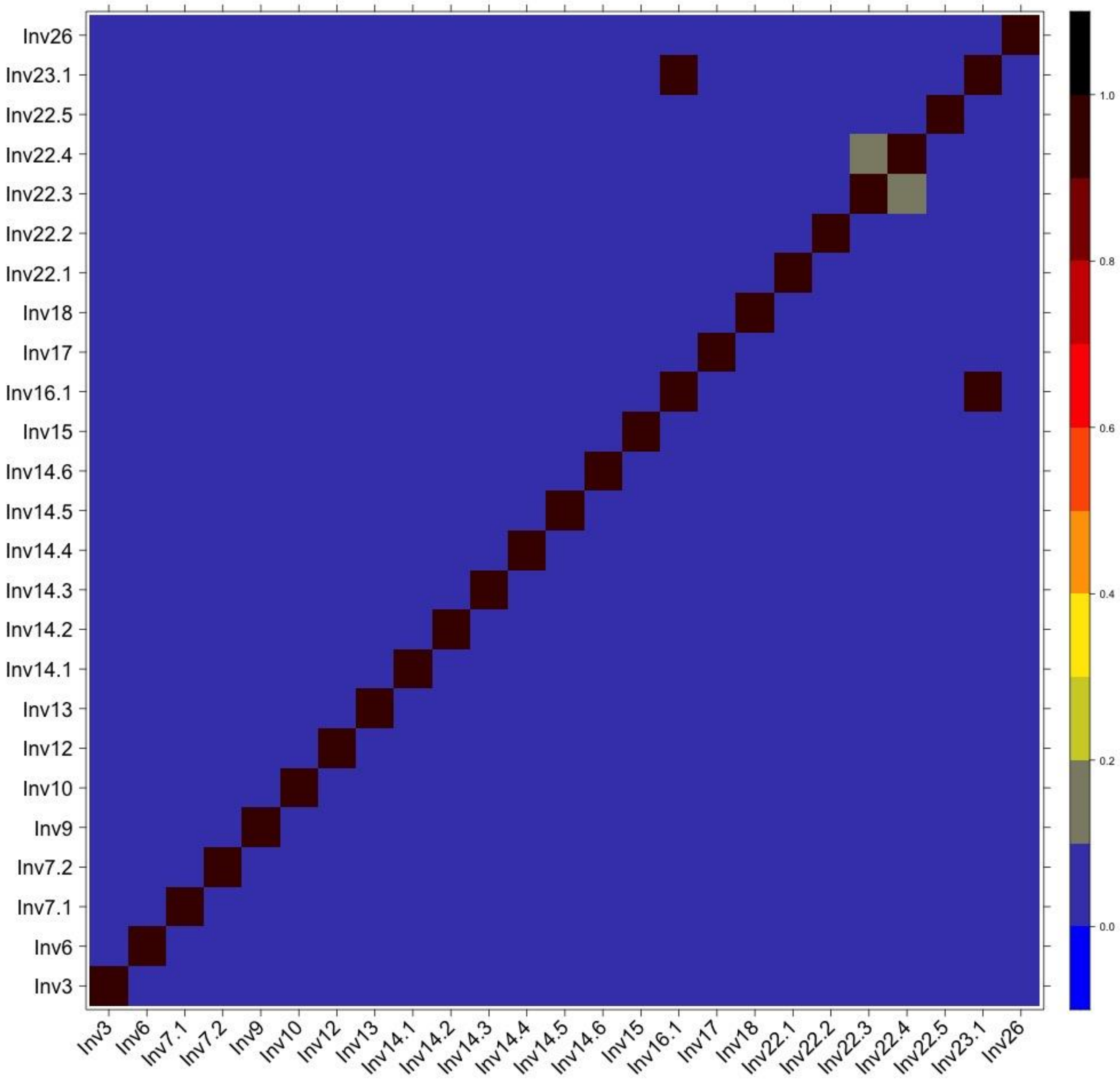

Figure S5 Correlation between inversion age and the major (MJ) inversion haplotype frequency.

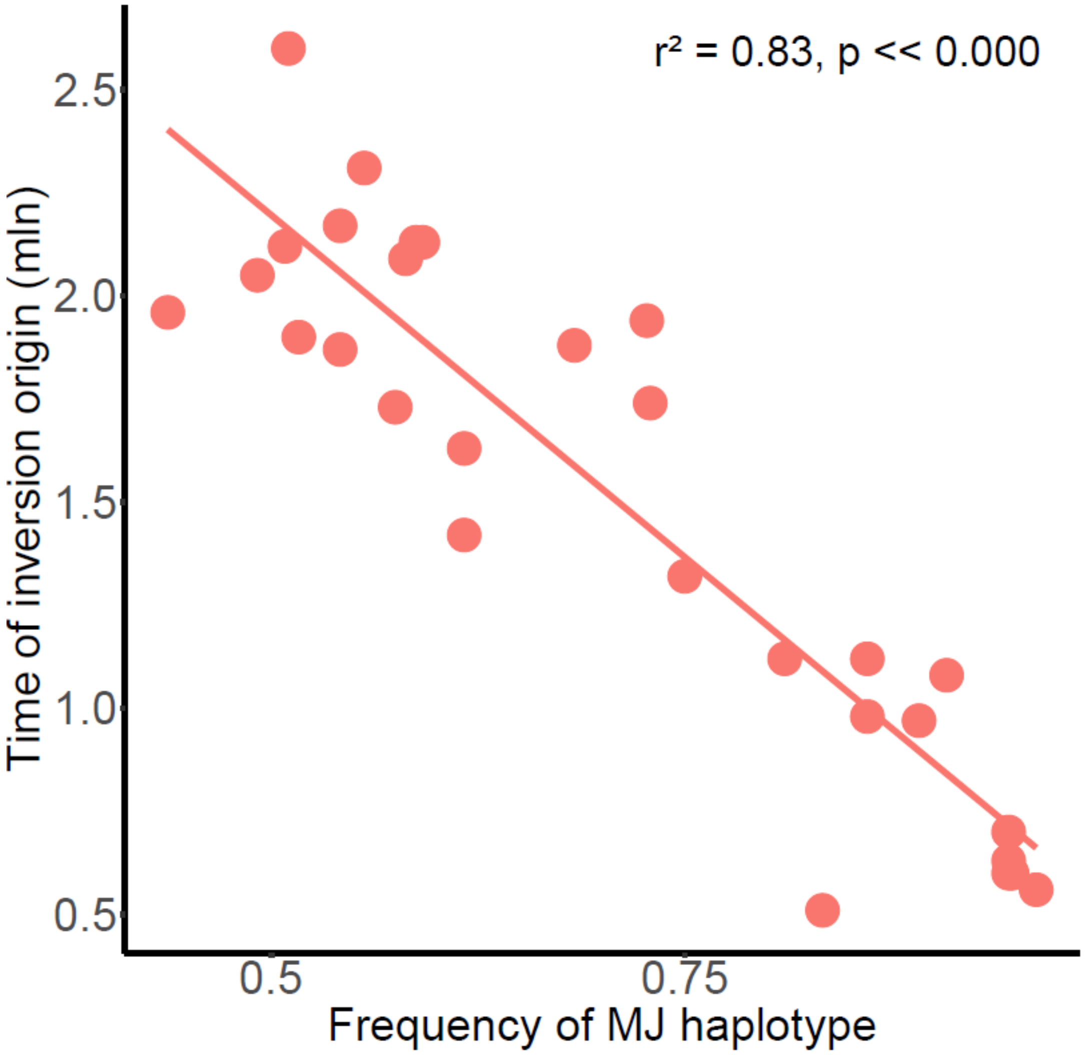

Figure S6 Inversion PCA analysis for northern (A) and southern (B) groups separately. Individuals colored by population.

Inv2

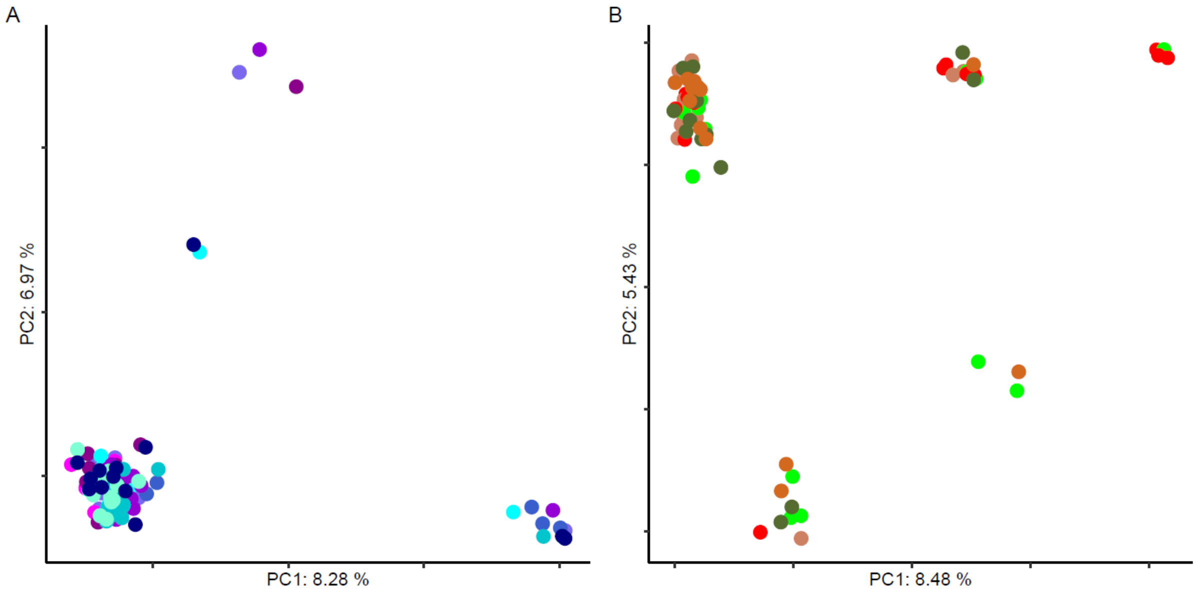

Inv3

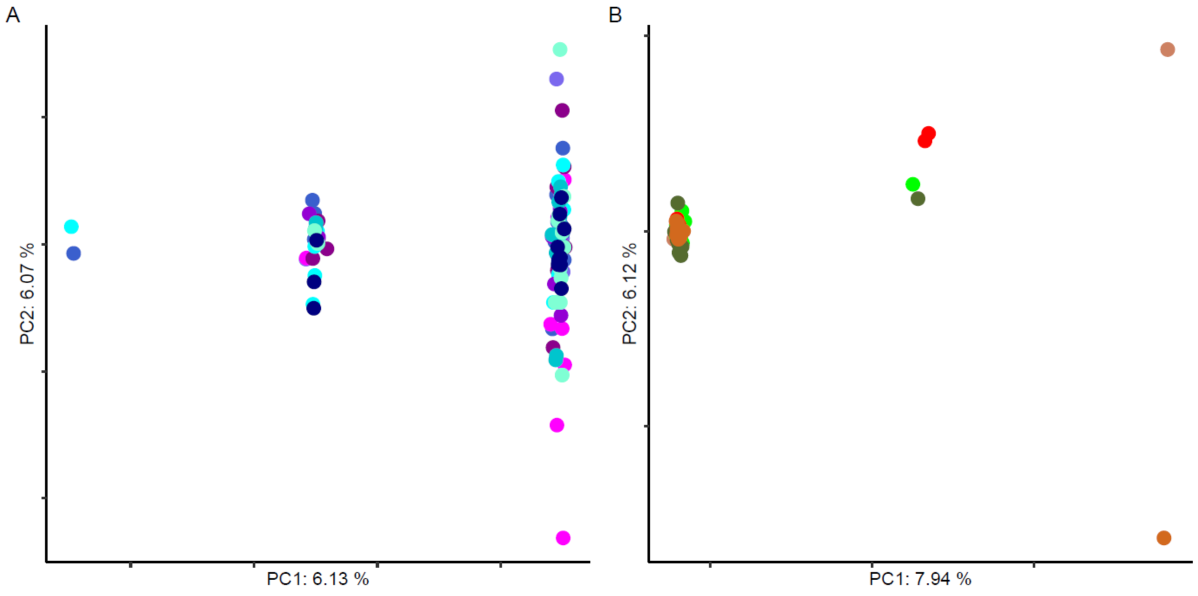

Inv5

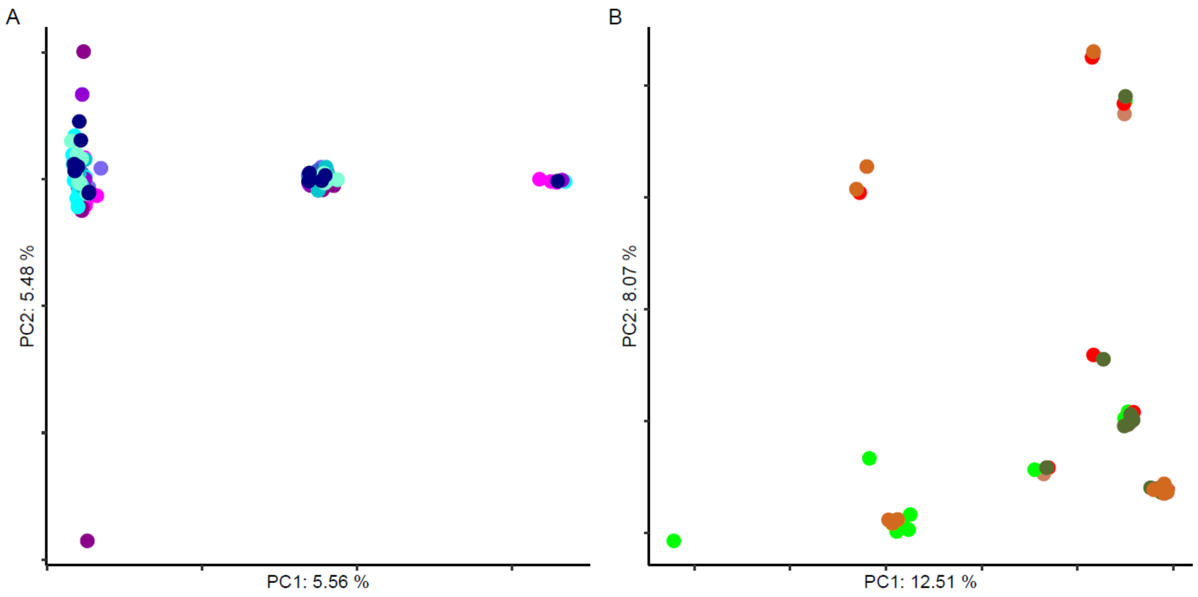

A
PC2: 5.48 %
PC1: 5.56 %
B
PC2: 8.07 %
PC1: 12.51 %

Inv6

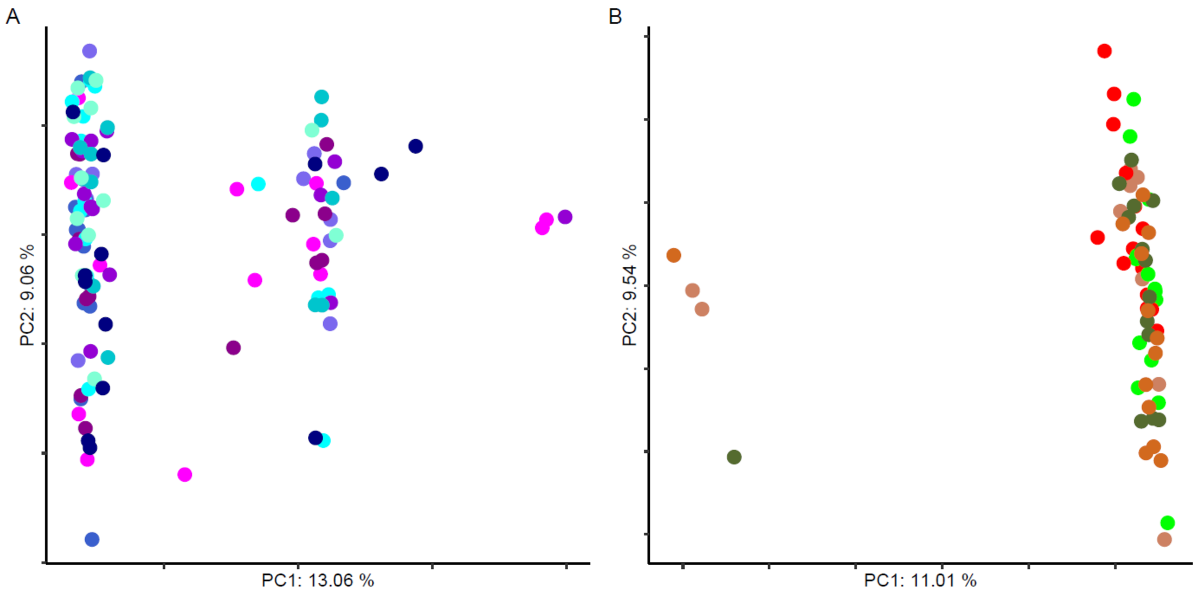

A
PC2: 9.06 %
PC1: 13.06 %
B
PC2: 9.54 %
PC1: 11.01 %

Inv7.1

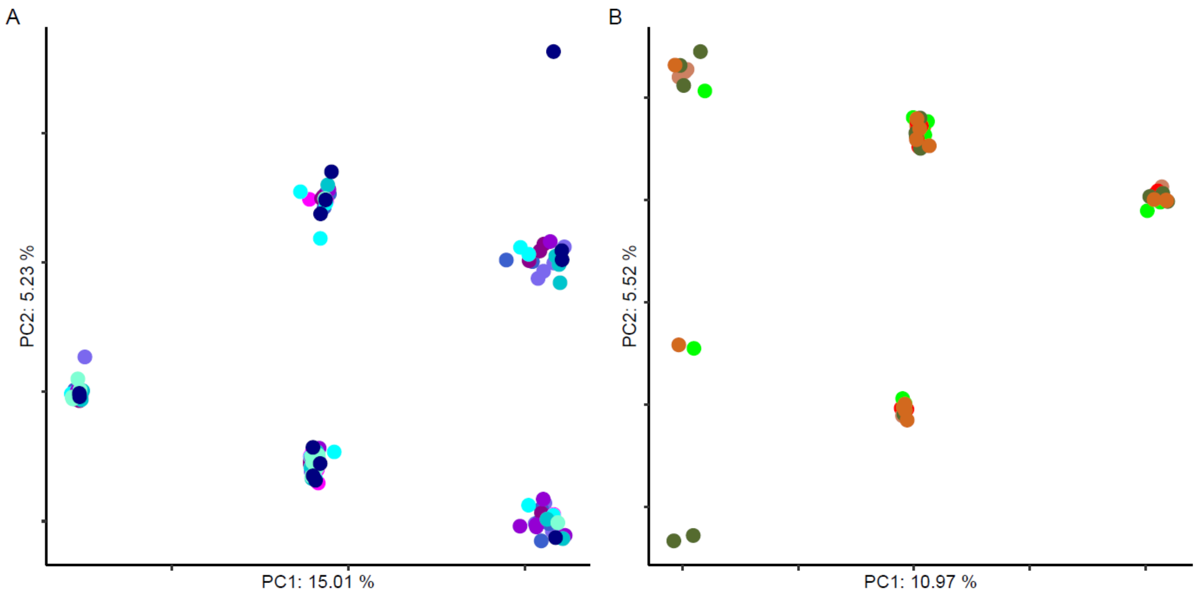
A
PC2: 5.23 %
PC1: 15.01 %
B
PC2: 5.52 %
PC1: 10.97 %

Inv7.2

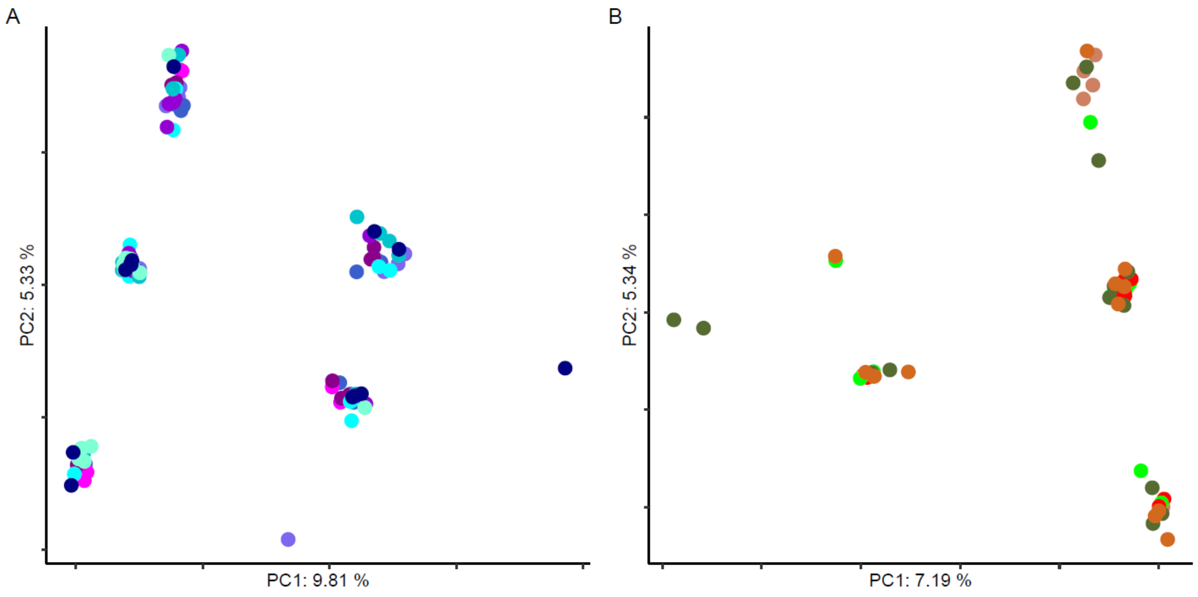
A
PC2: 5.33 %
PC1: 9.81 %
B
PC2: 5.34 %
PC1: 7.19 %

Inv9

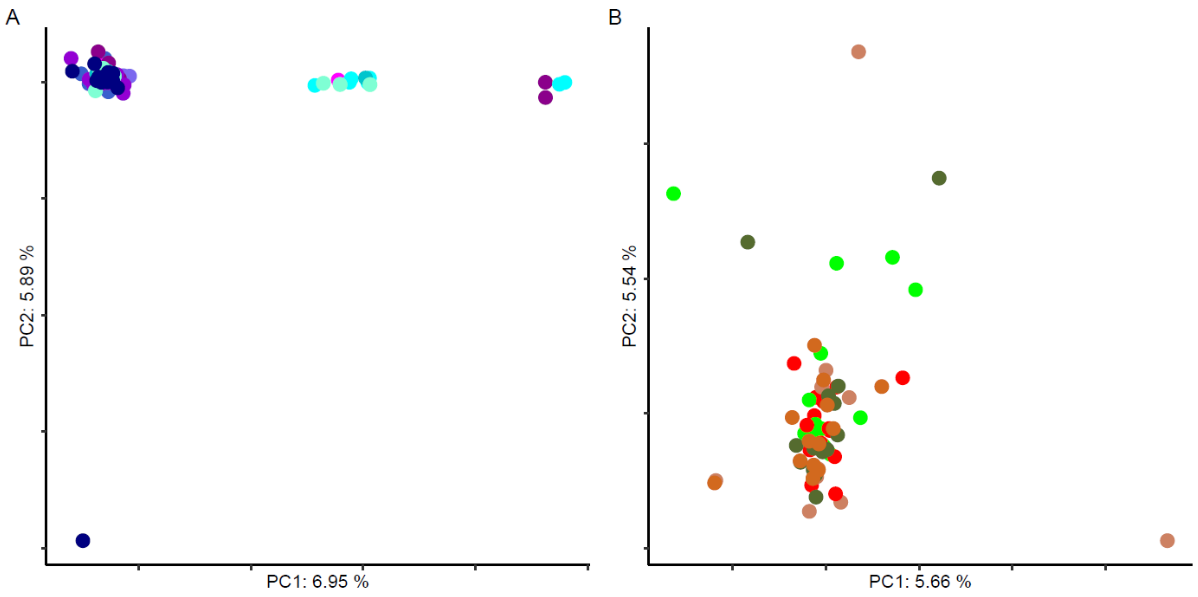
A
PC2: 5.89 %
PC1: 6.95 %
B
PC2: 5.54 %
PC1: 5.66 %

Inv10

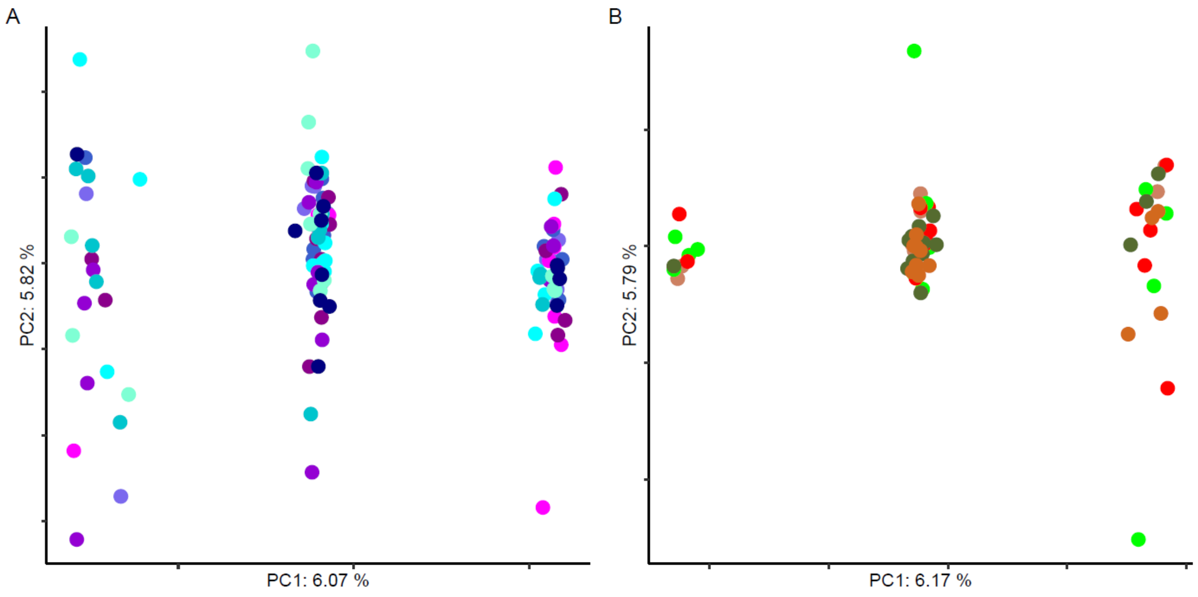
A
PC2: 5.82 %
PC1: 6.07 %
B
PC2: 5.79 %
PC1: 6.17 %

Inv12

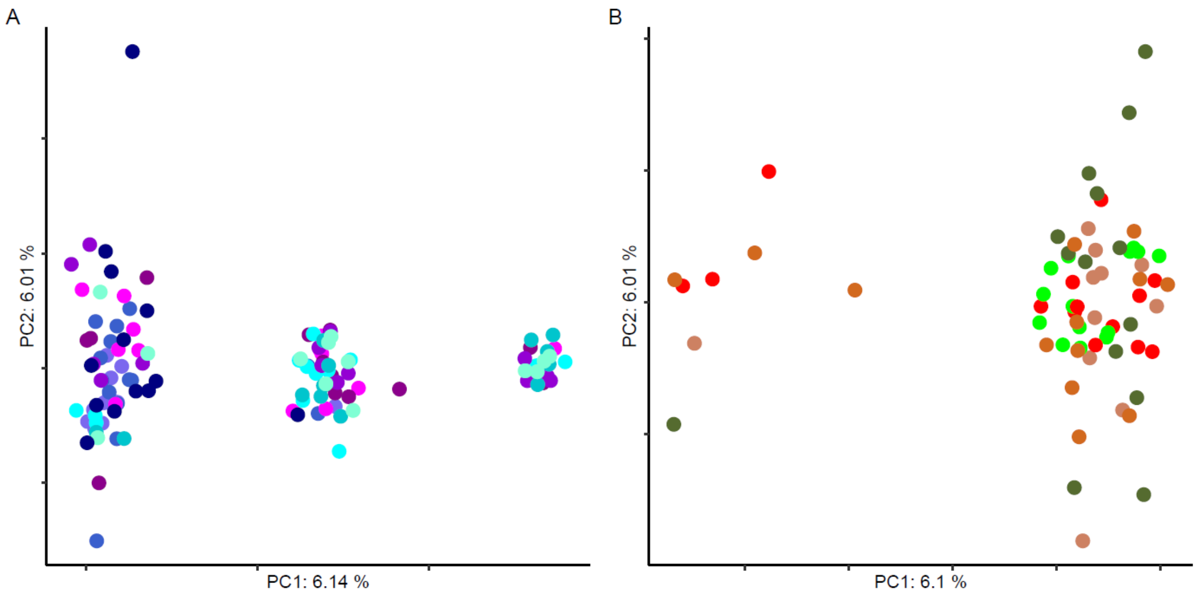
A
PC2: 6.01 %
PC1: 6.14 %
B
PC2: 6.01 %
PC1: 6.1 %

Inv13

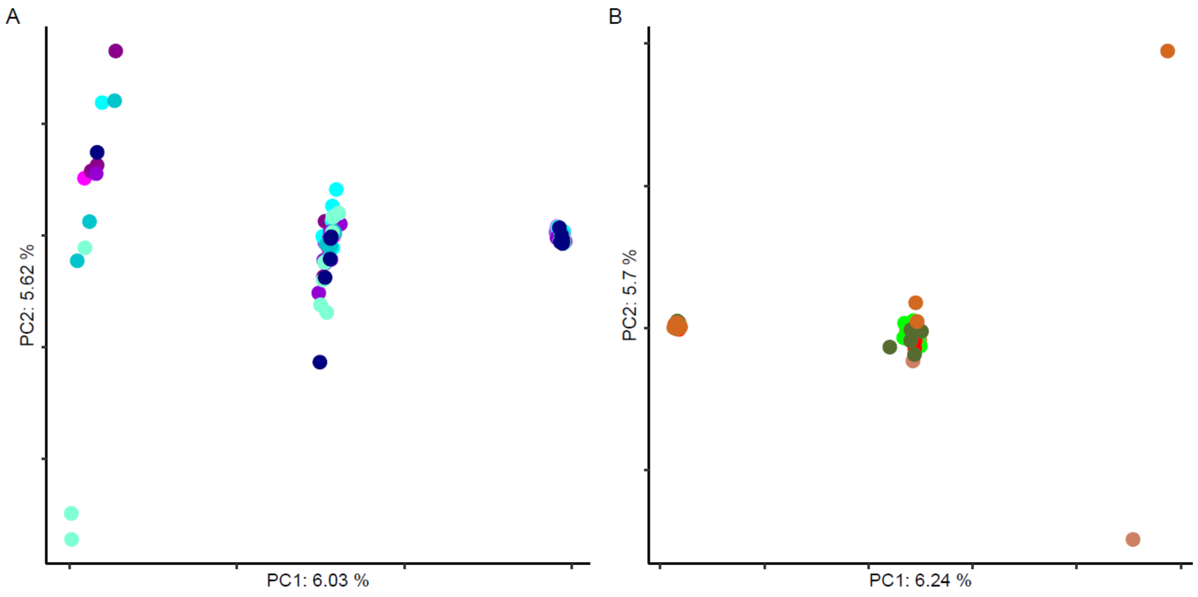
A
PC2: 5.62 %
PC1: 6.03 %
B
PC2: 5.7 %
PC1: 6.24 %

Inv14.1

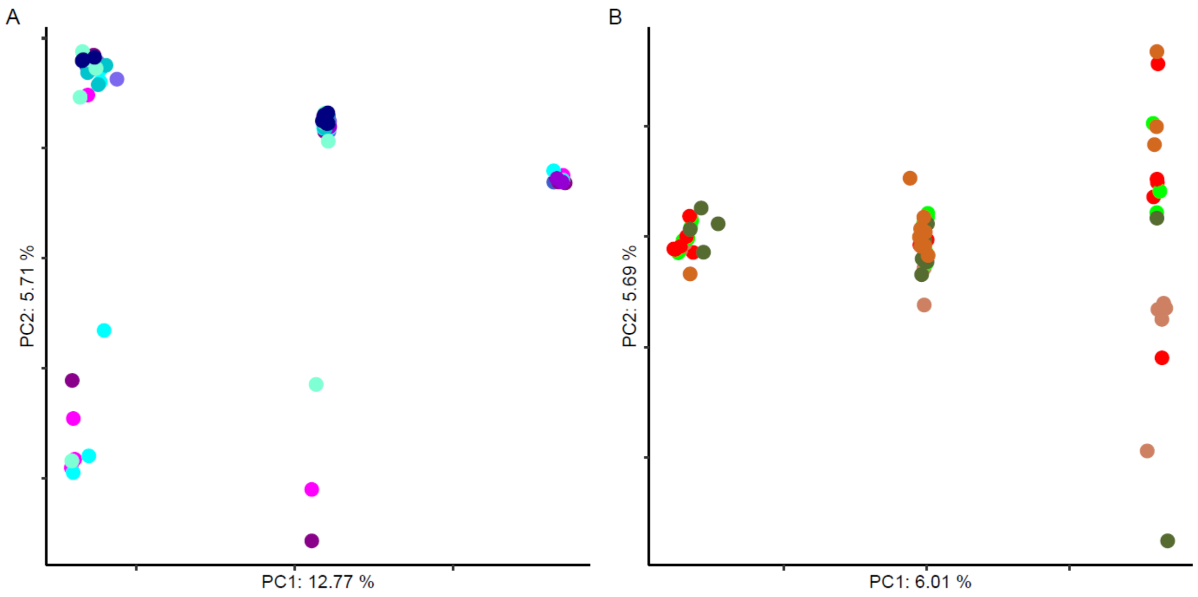

Inv14.2

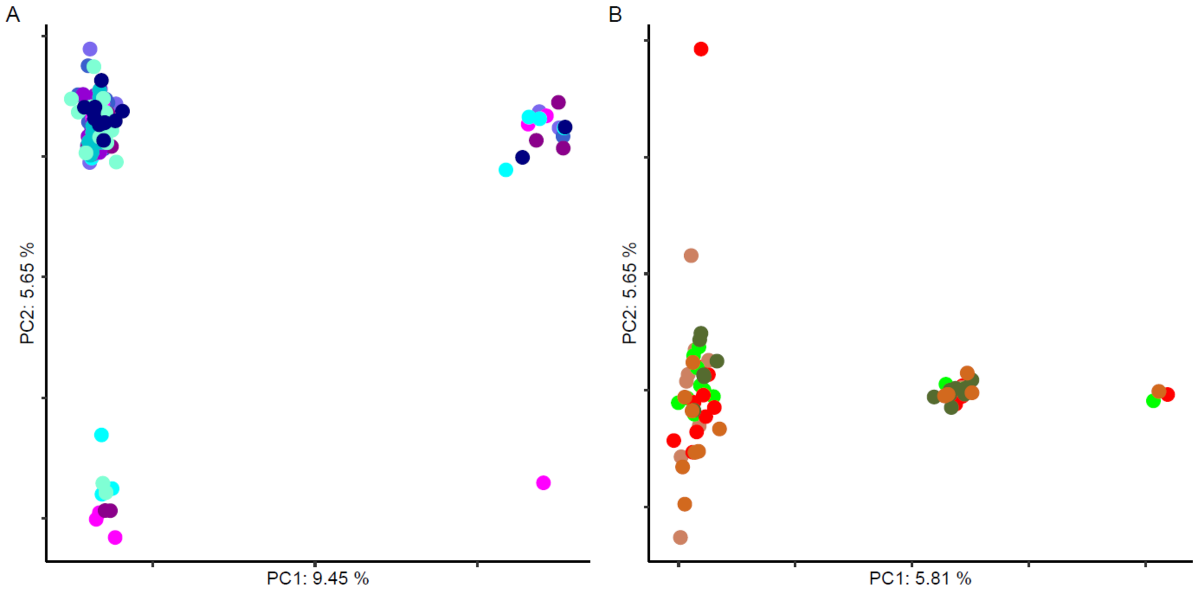

Inv14.3

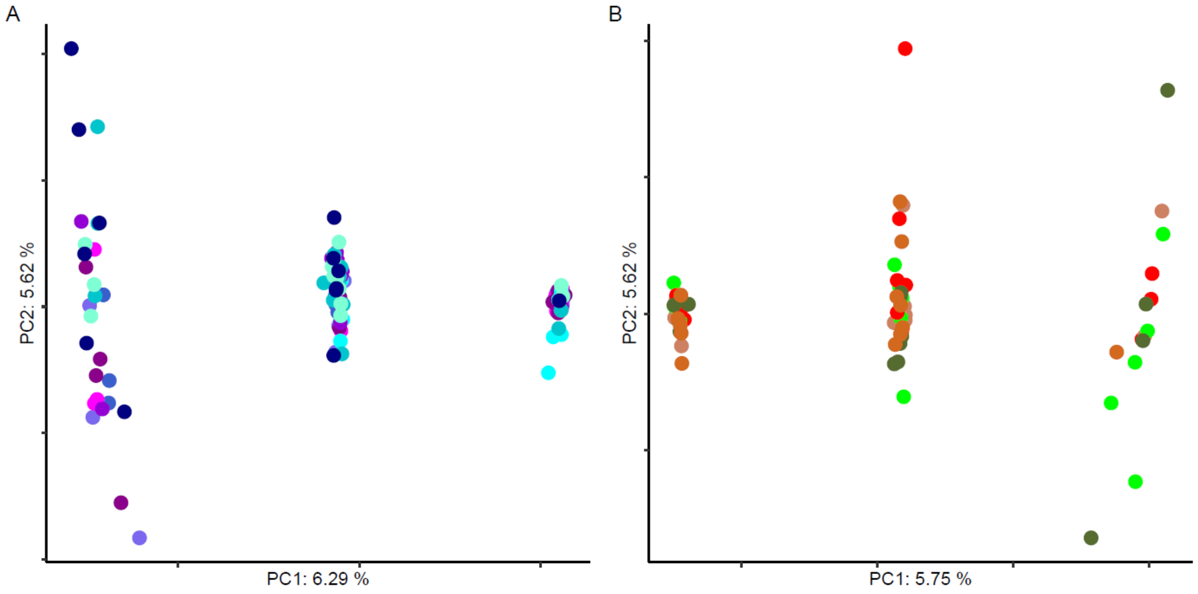

Inv14.4

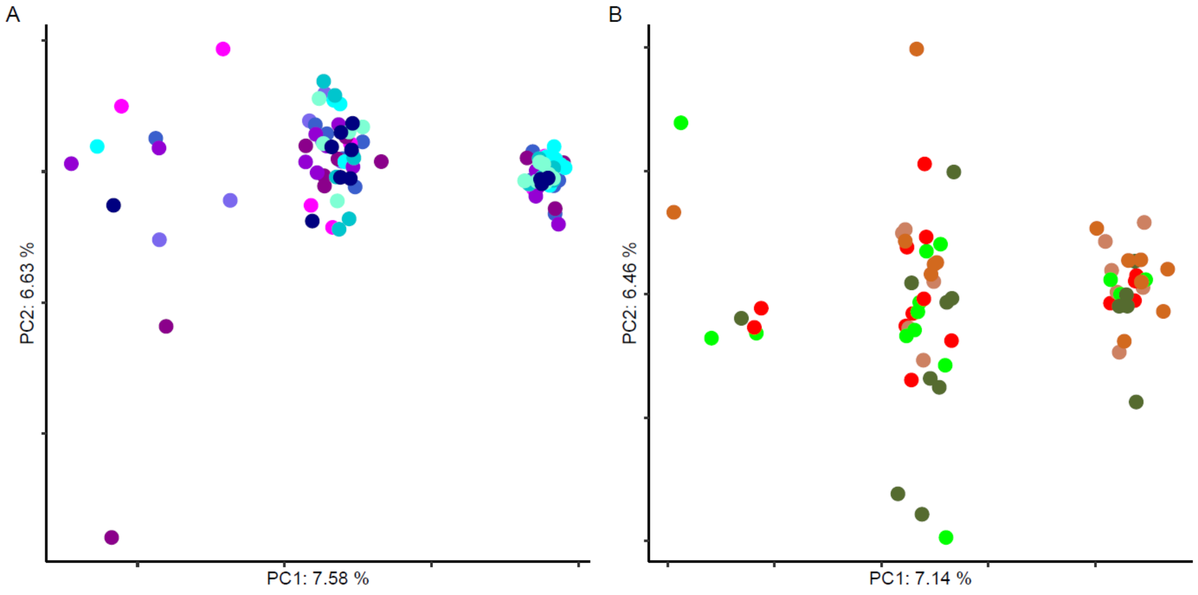

Inv14.5

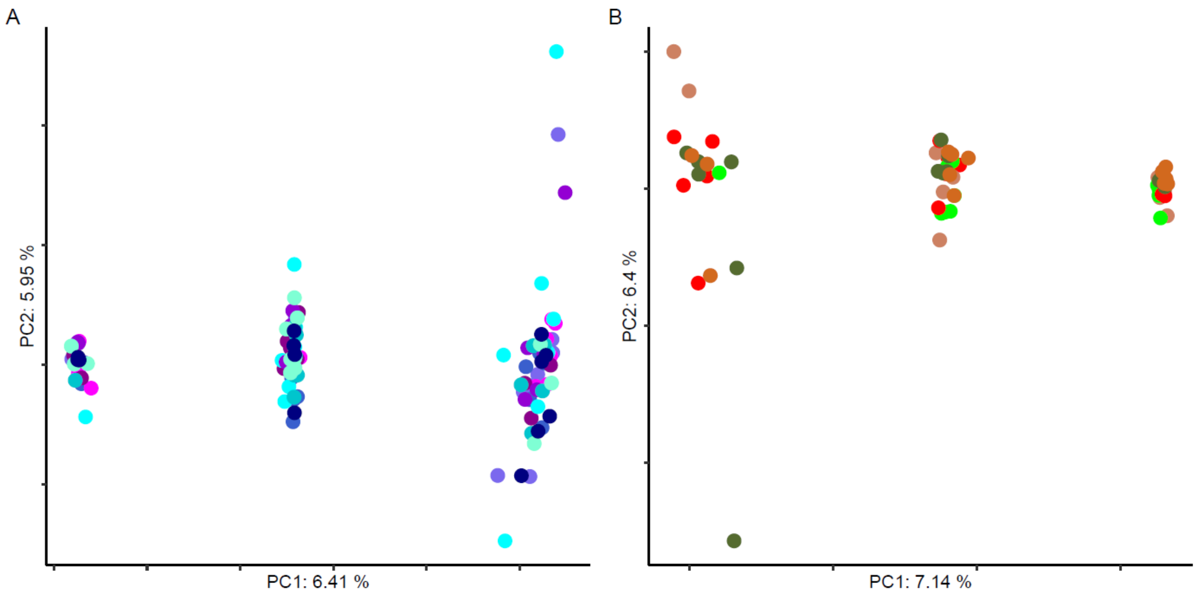

Inv15

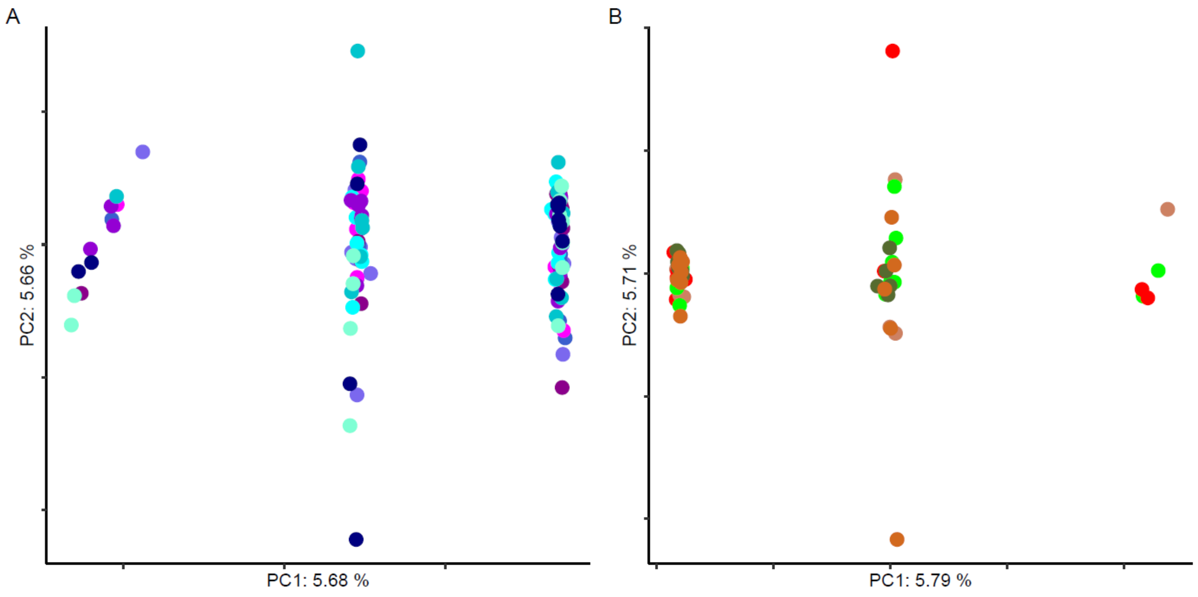

Inv16.1

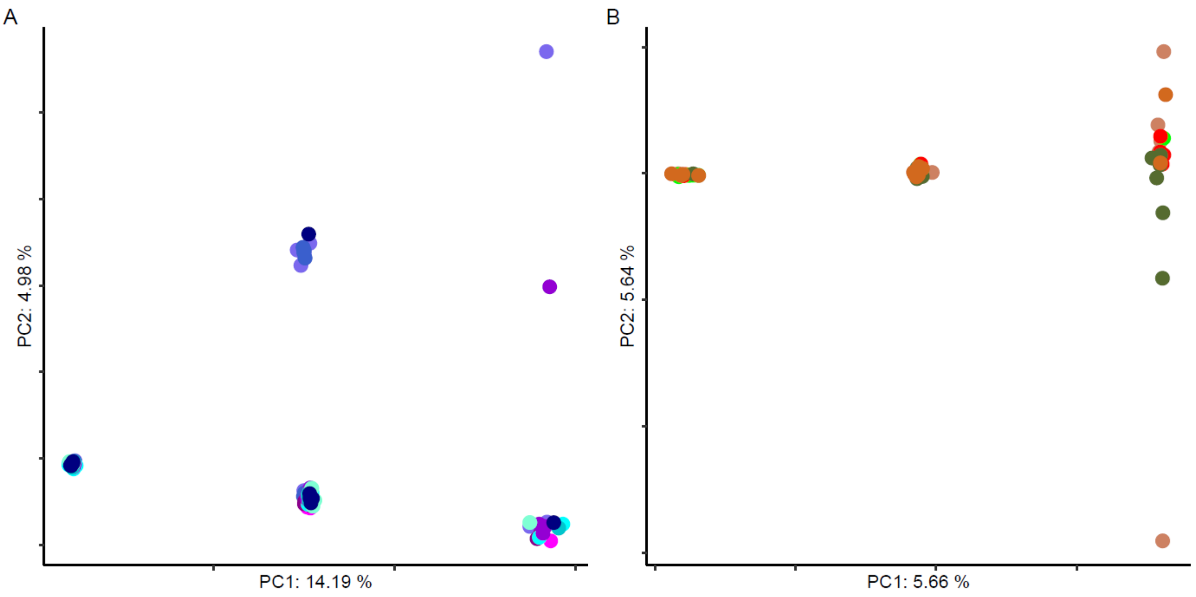

Inv17

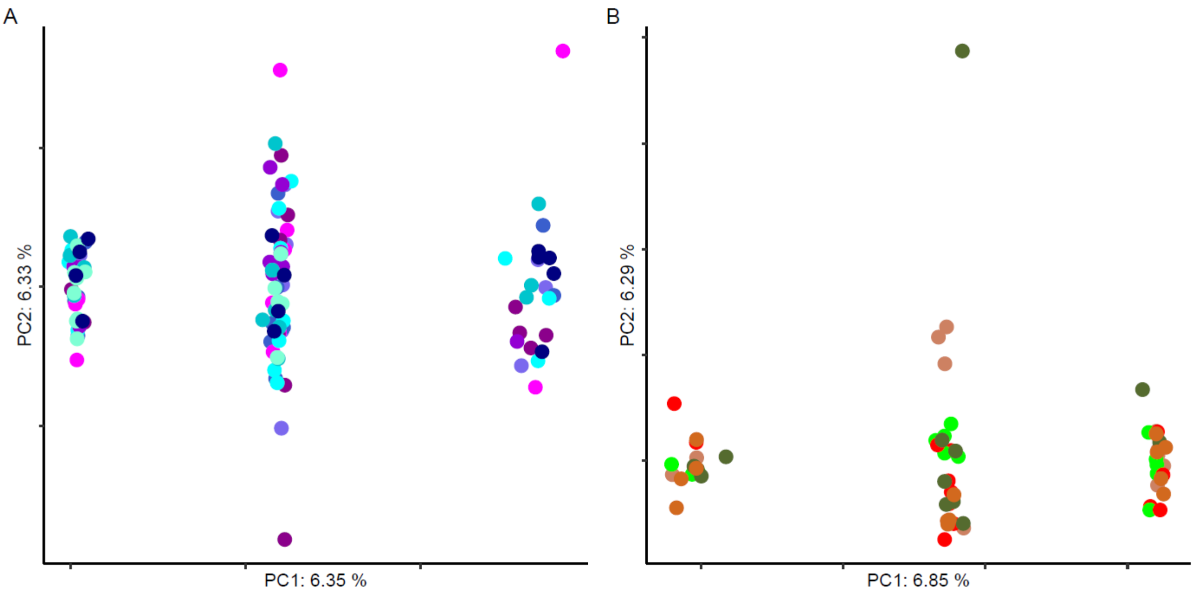

Inv18

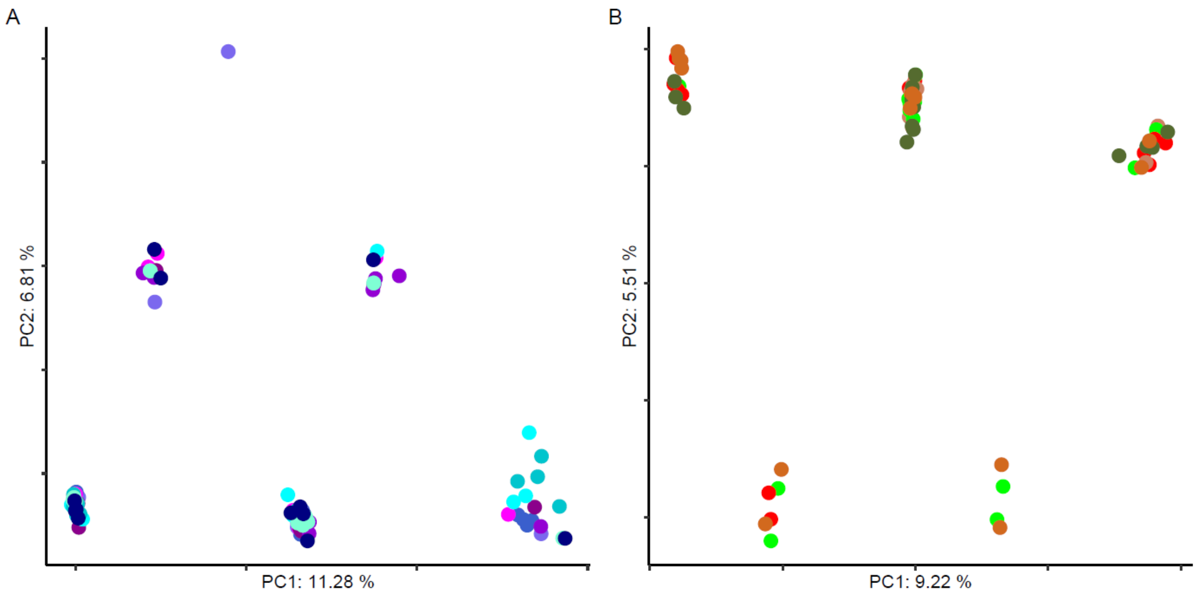

Inv22.1

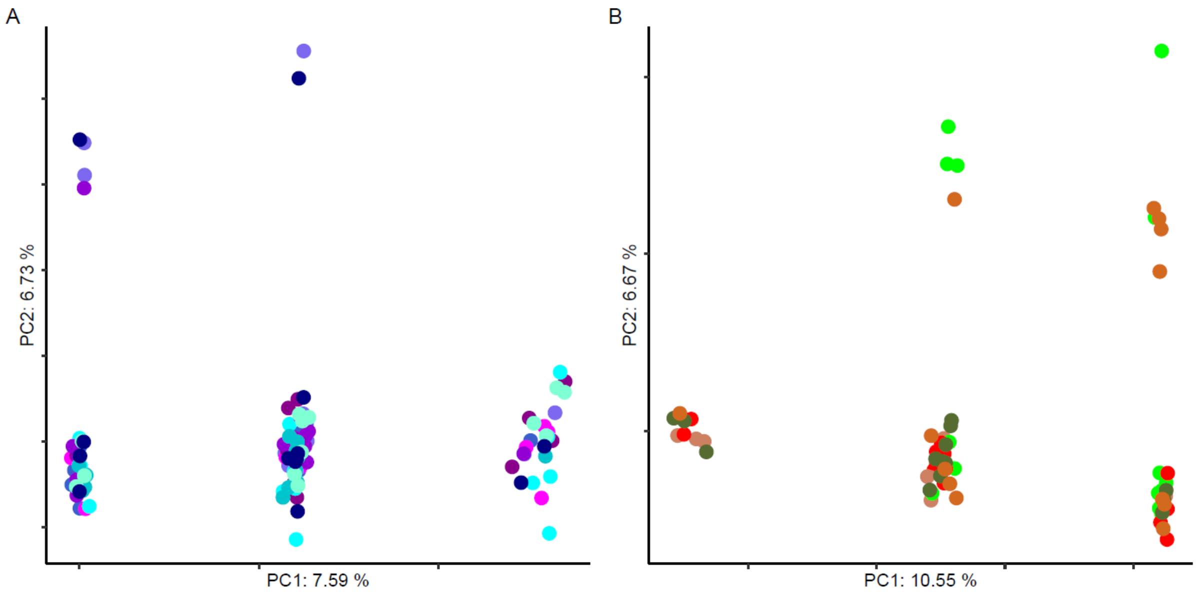

Inv22.2

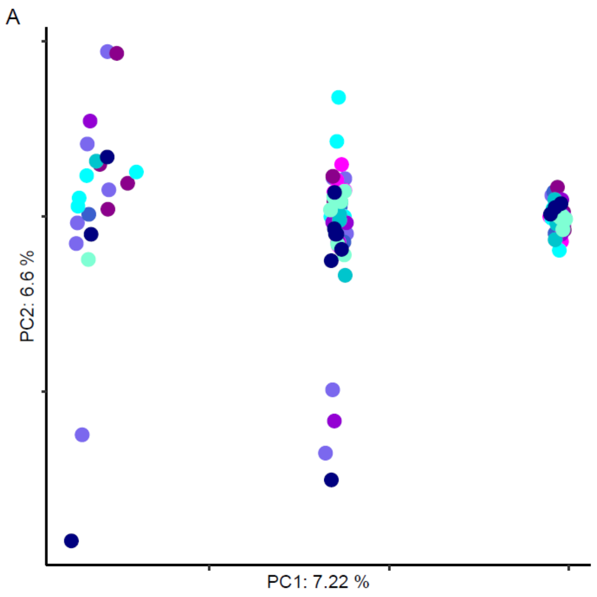

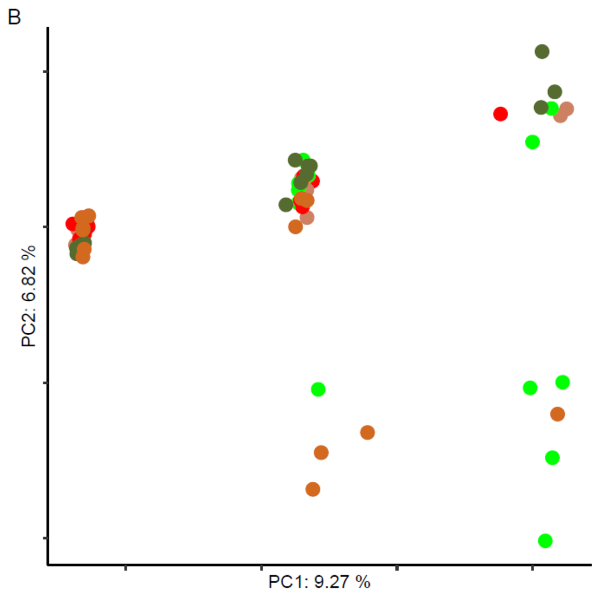

Inv22.3

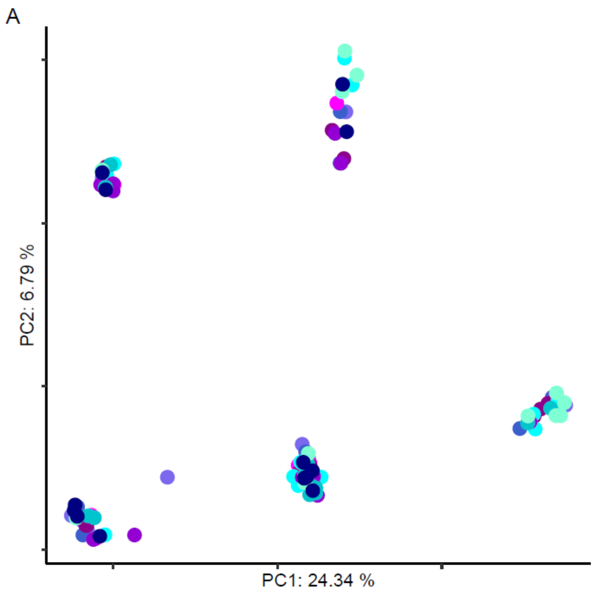

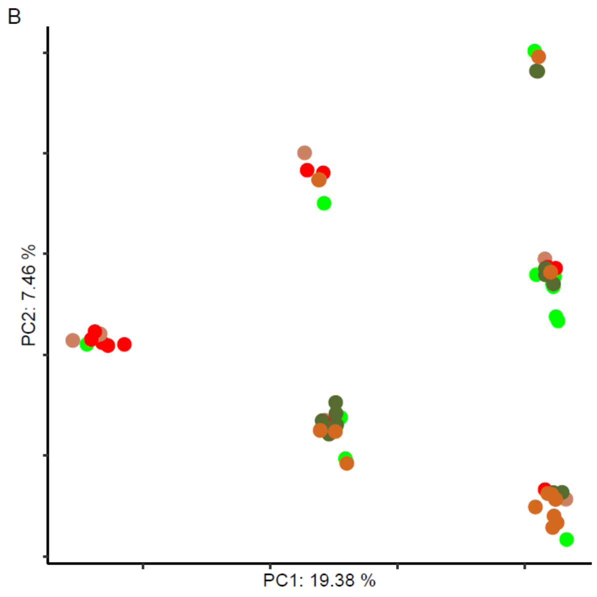

Inv22.4

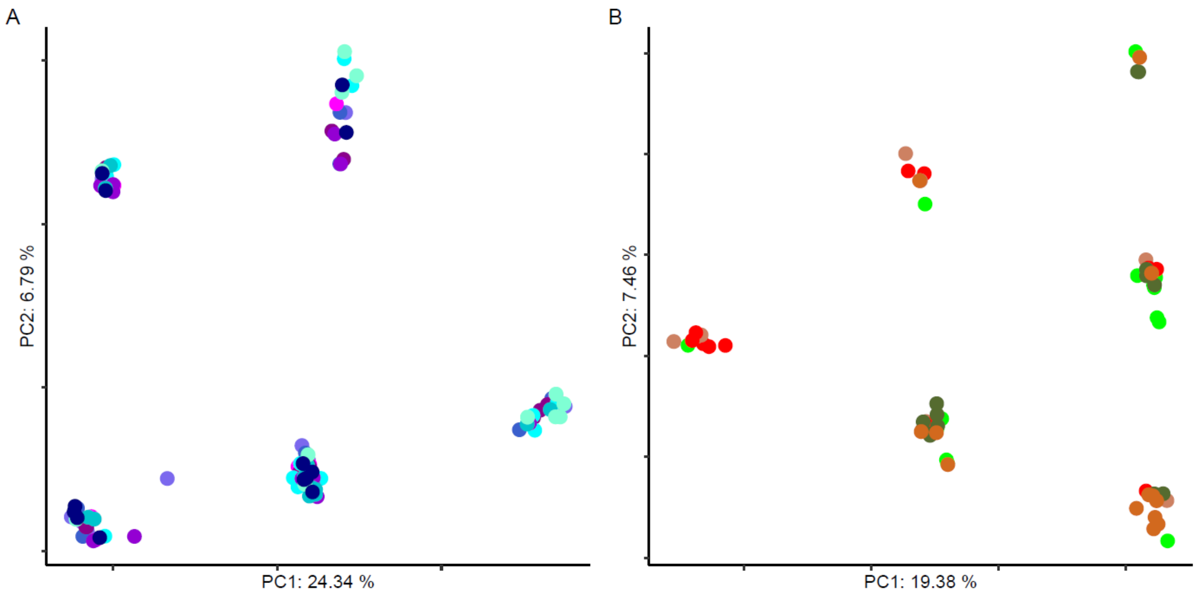

Inv23.1

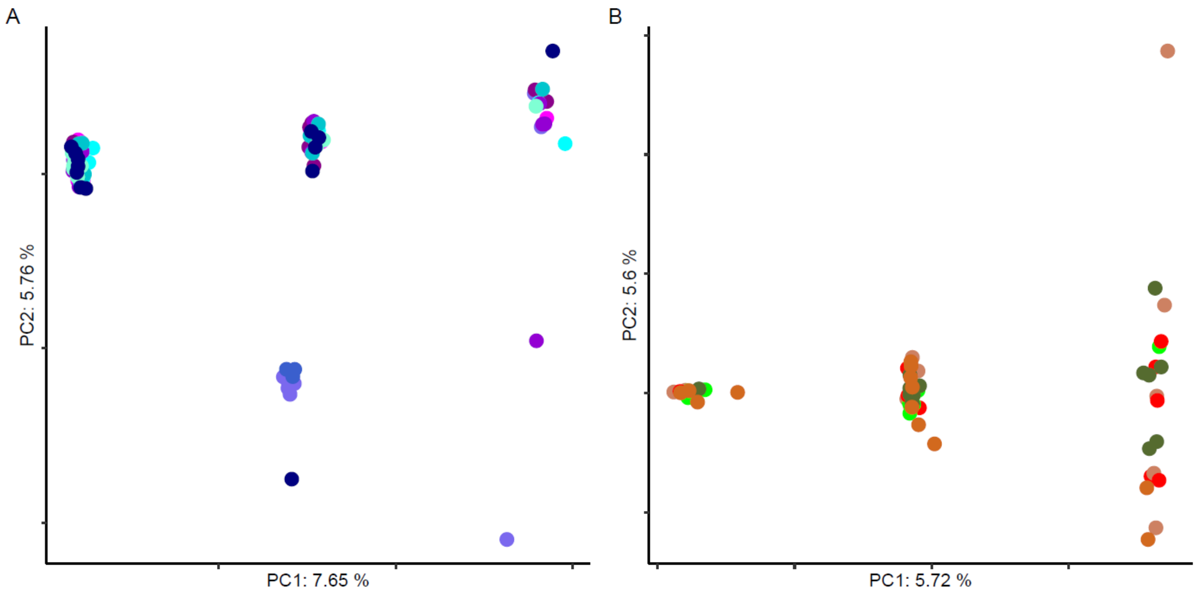

Inv26

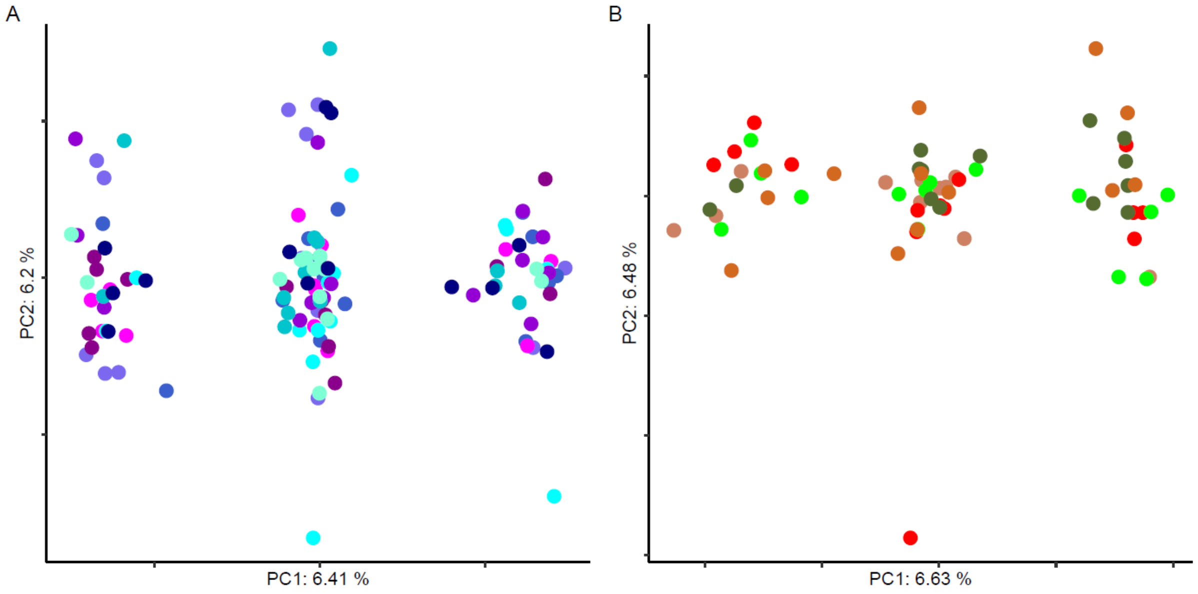

Figure S7 Mean likelihoods from 10 replicate runs for each tested K (1-5). CLUMPAK results: Delta(K=2) = 1254766.6813024; Delta(K=3) = 1.41919929347898; Delta(K=4) = 2.00718351826507.

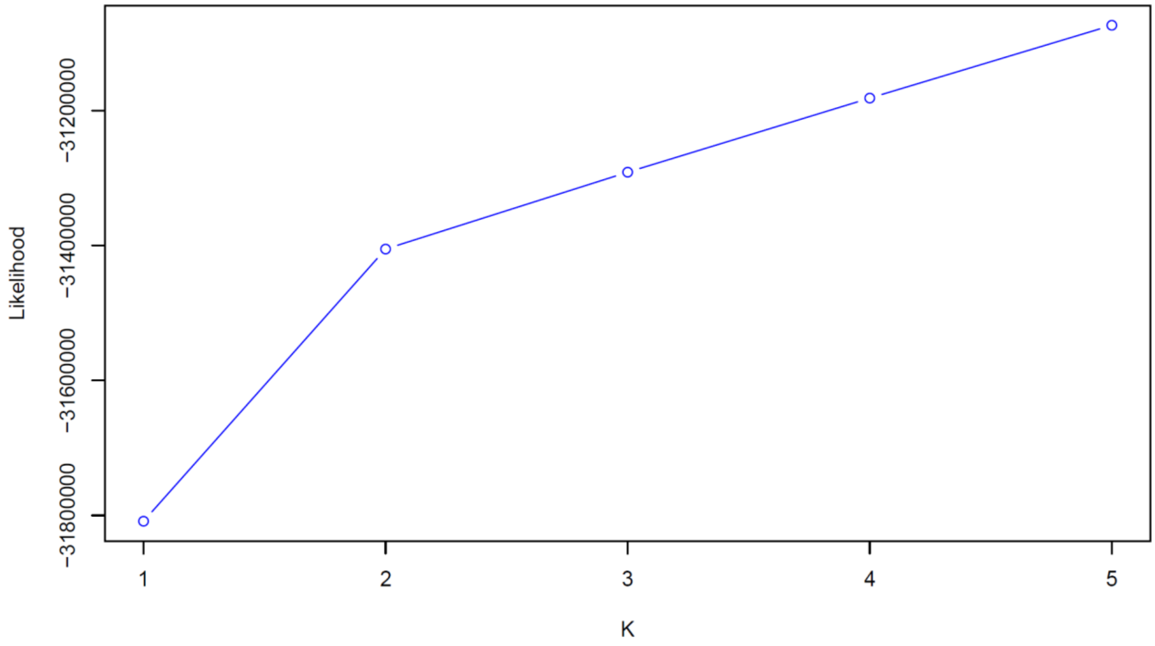

Figure S8 Nucleotide diversity, Tajima's D and d_{xy} calculated in 100kb windows along 36 contigs from the *Ips typographus* genome. Nucleotide diversity and Tajima's D were calculated per populations: northern, southern and Polish populations are shown in different blue, red/green and yellow colors, respectively. D_{xy} was calculated between southern and northern groups.

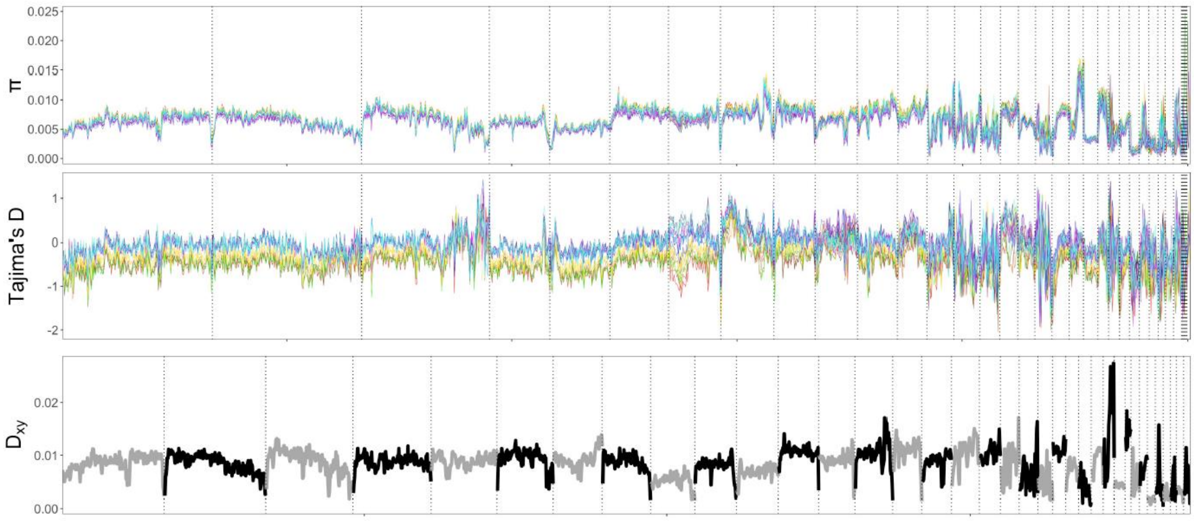

Figure S9 Correlation between nucleotide diversity (per *Ips typographus* population) and the latitude where the population was collected.

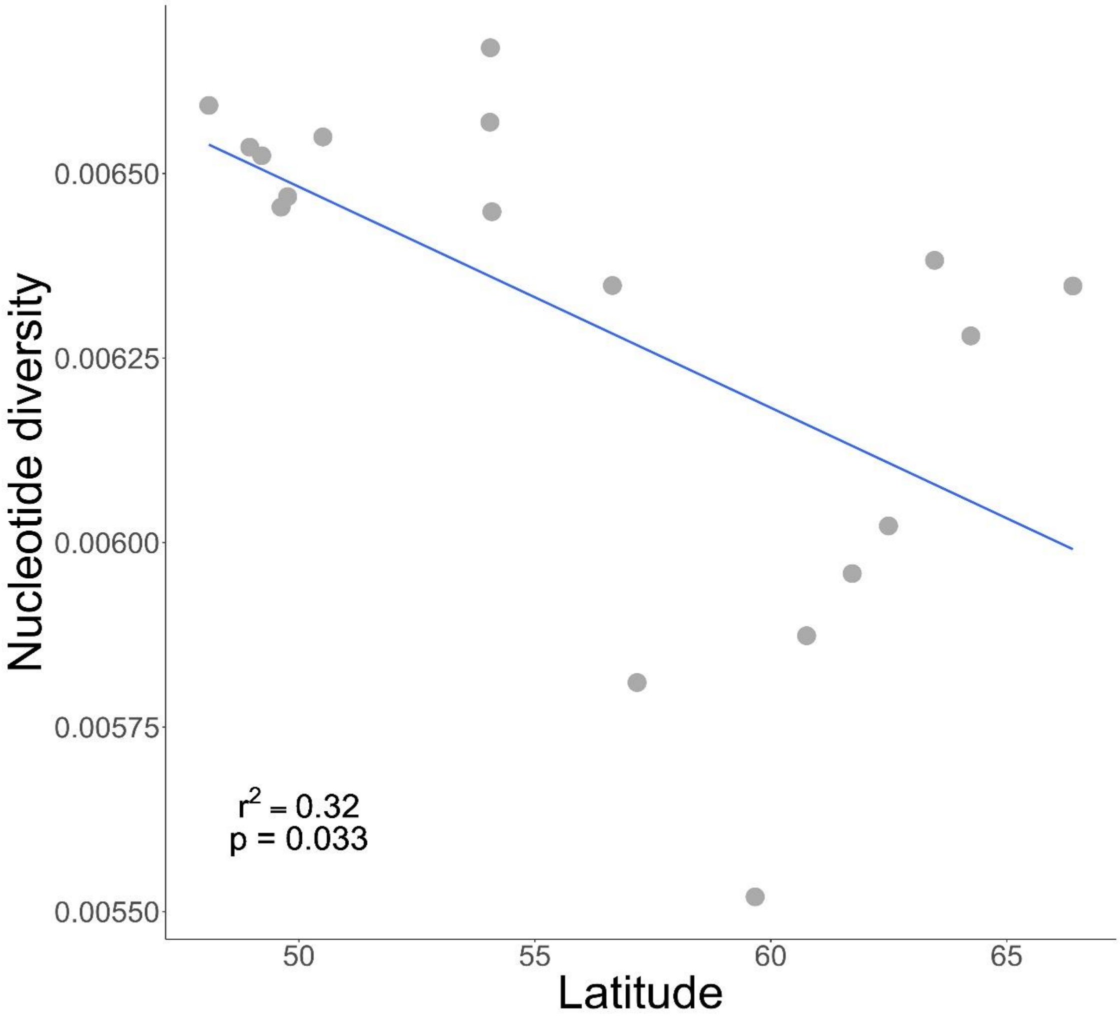

Figure S10 PC1 versus PC2 for inversion regions based on the dataset that included *Ips typographus* individuals with known diapause phenotype. Only inversions that could be genotyped in all studied populations were included. Purple dots represent individuals with obligate diapause and orange dots individuals with facultative diapause. Grey dots represent *I. typographus* individuals of unknown diapause phenotype.

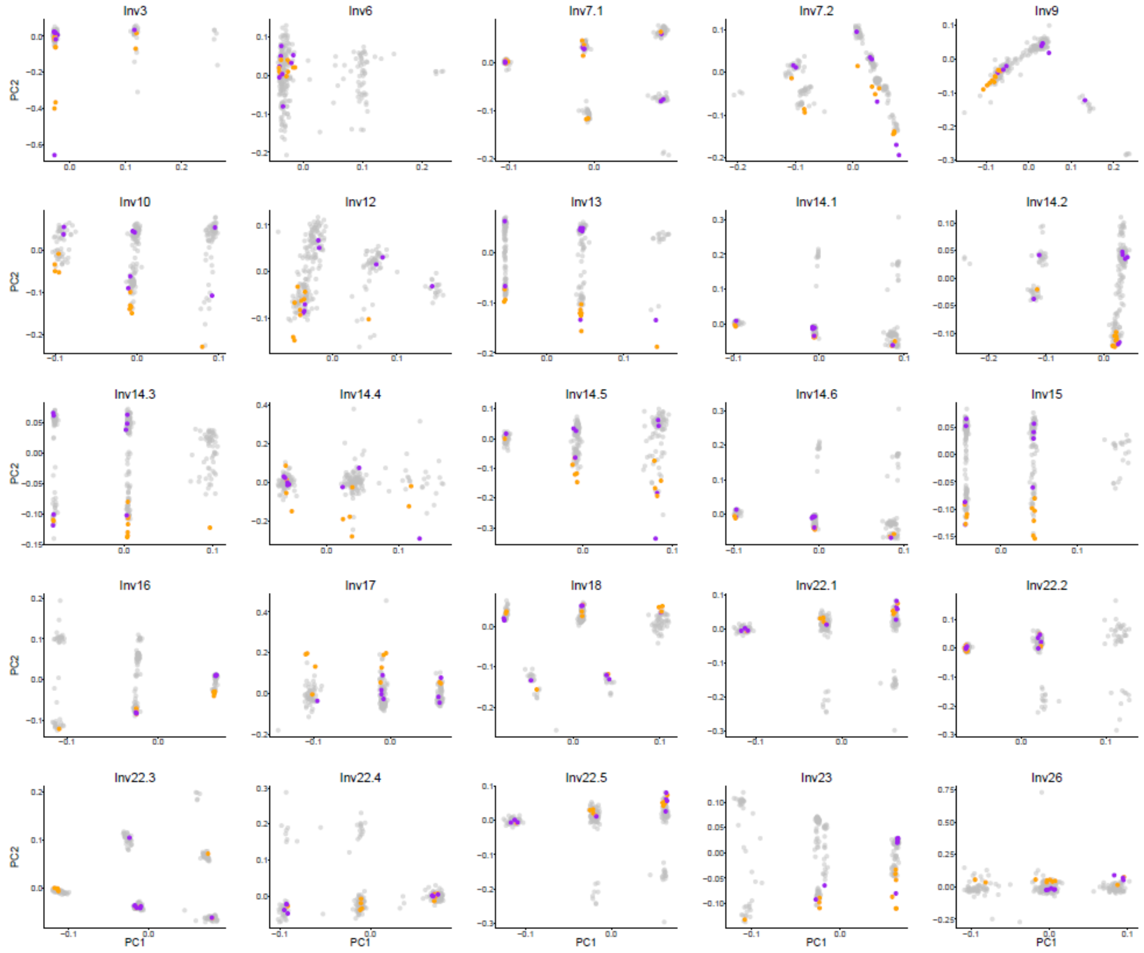

Figure S11 Genome-wide genetic differentiation (F_{ST}) along 36 contigs from the *Ips typographus* genome. Contigs are ordered by length and contigs boundaries are indicated with vertical lines. Red line shows F_{ST} between individuals with facultative and obligate diapause (A) and blue line (B) shows F_{ST} between southern and northern beetle populations. Panel C shows both lines together for easier comparison.

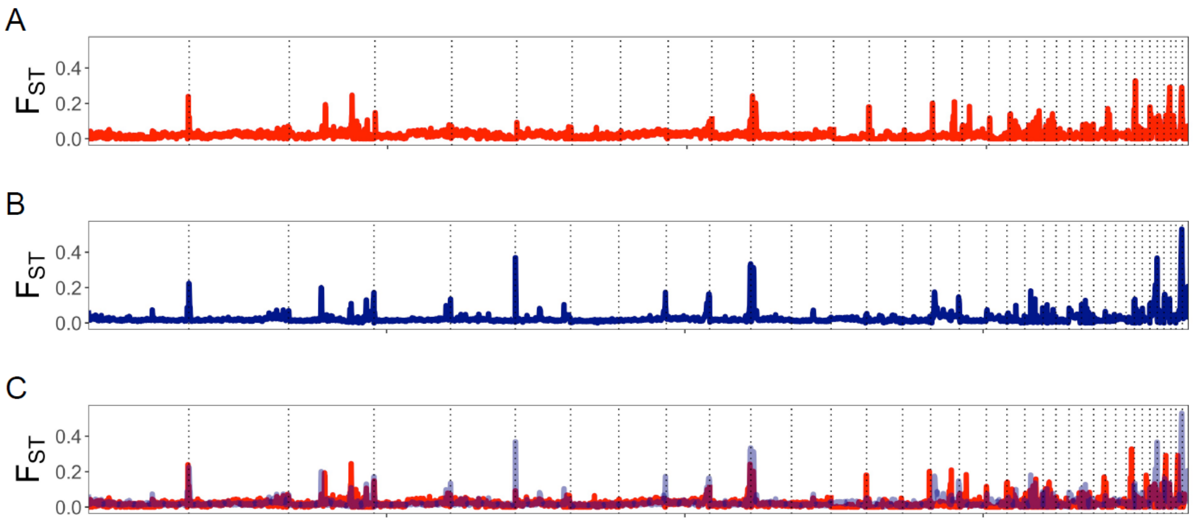

Figure S12 Neighbor-joining trees showing examples of inversion haplotypes in the *Ips typographus* genome with (a, b, e) and without (c, d, f) haplotype structuring. a and b show two alternative inversion haplotypes of inversion Inv16.1, minor and major, respectively; c and d show two alternative inversion haplotypes of Inv26, minor and major, respectively. e and f show minor (triangles) and major (dots) inversion haplotypes on one tree, for Inv16.1 and Inv26, respectively. Colors correspond to geographic origin of haplotypes (red: southern populations; yellow: Polish populations; blue: northern populations).

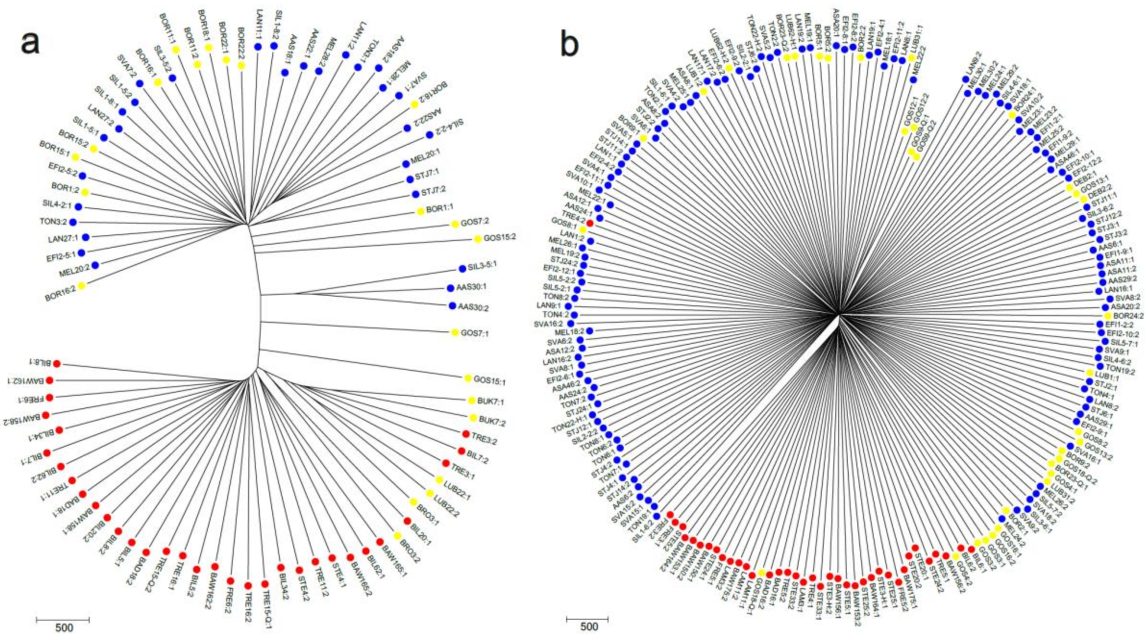

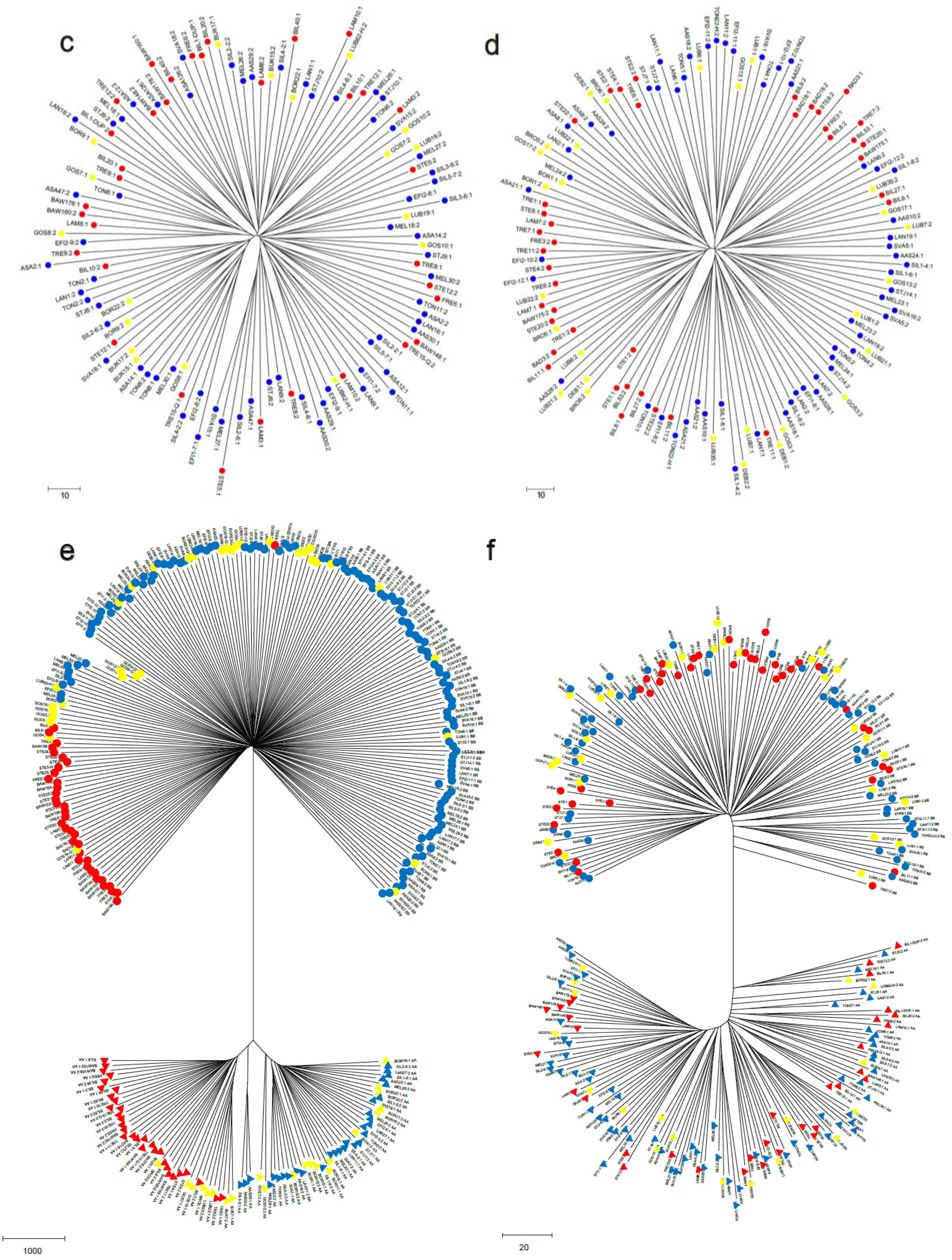
c
d
10
10
e
f
1000
20

Figure S13 Examples of NGSadmix results for three inversion regions (a: Inv13; b: Inv15; c: Inv17) in the *Ips typographus* genome showing clear separation into inversion homozygotes (red or blue individuals) or inversion heterozygotes (individuals with red and blue ancestry in approximately equal proportions (the admixture proportions are not exactly 50:50 due to data noise, e.g. different patterns of missing data among individuals or conversion events between two haplotypes). Individuals from different beetle populations are separated by vertical black lines.

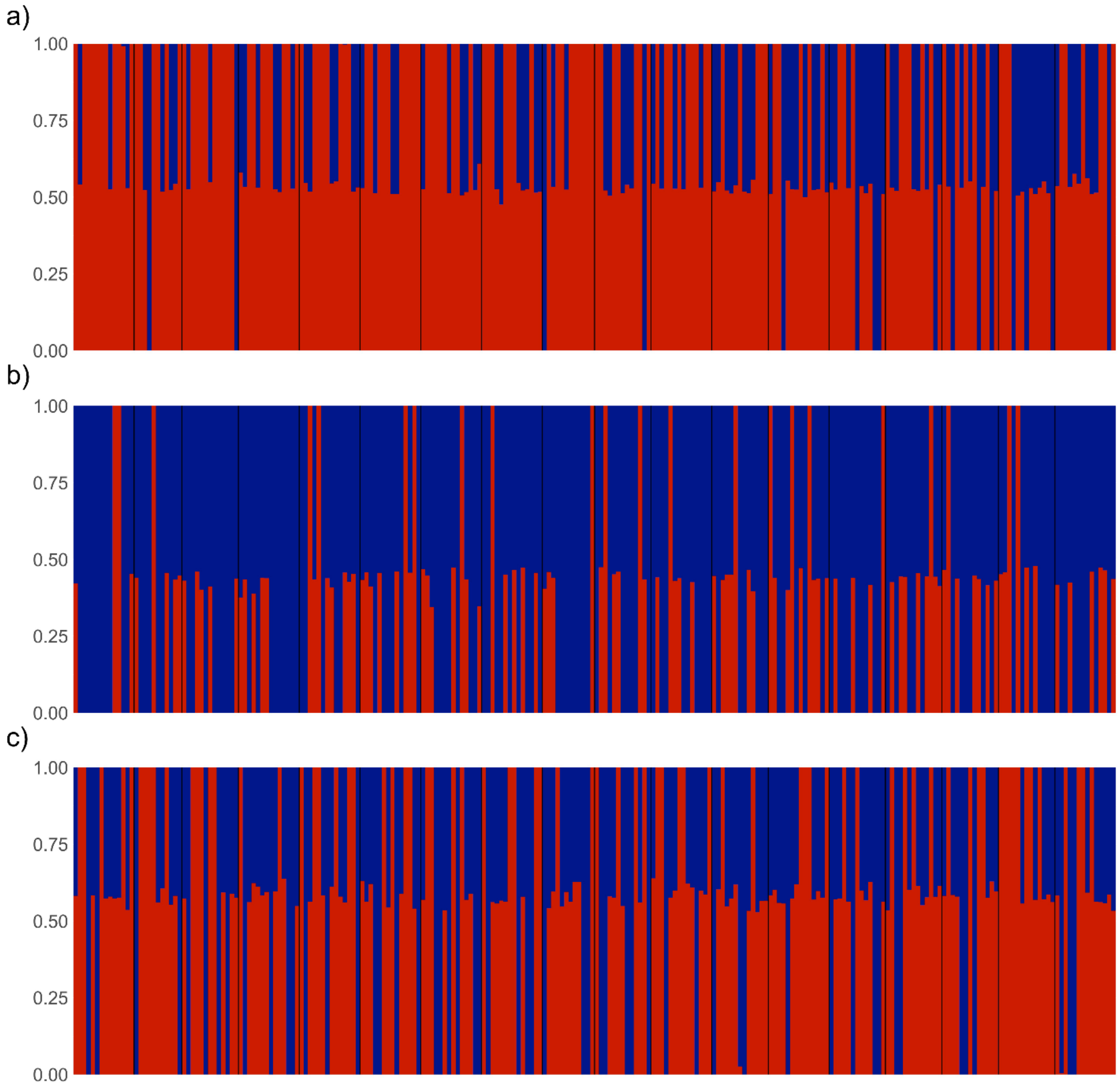

Figure S14 Genotype-environmental association (GEA) analysis of *Ips typographus* populations collected throughout Europe. The maps show rasters of final environmental variables extracted from a 50 km radius around each population locality. BIO1 (Annual Mean Temperature), BIO2 (Mean Diurnal Range). BIO3 (Isothermality). BIO4 (Temperature Seasonality), BIO5 (Max Temperature of Warmest Month). BIO6 (Min Temperature of Coldest Month), BIO7 (Temperature Annual Range), BIO8 (Mean Temperature of Wettest Quarter). BIO9 (Mean Temperature of Driest Quarter), BIO10 (Mean Temperature of Warmest Quarter), BIO11 (Mean Temperature of Coldest Quarter), BIO12 (Annual Precipitation), BIO13 (Precipitation of Wettest Month), BIO14 (Precipitation of Driest Month), BIO15 (Precipitation Seasonality), BIO16 (Precipitation of Wettest Quarter), BIO17 (Precipitation of Driest Quarter), BIO18 (Precipitation of Warmest Quarter), and BIO19 (Precipitation of Coldest Quarter).

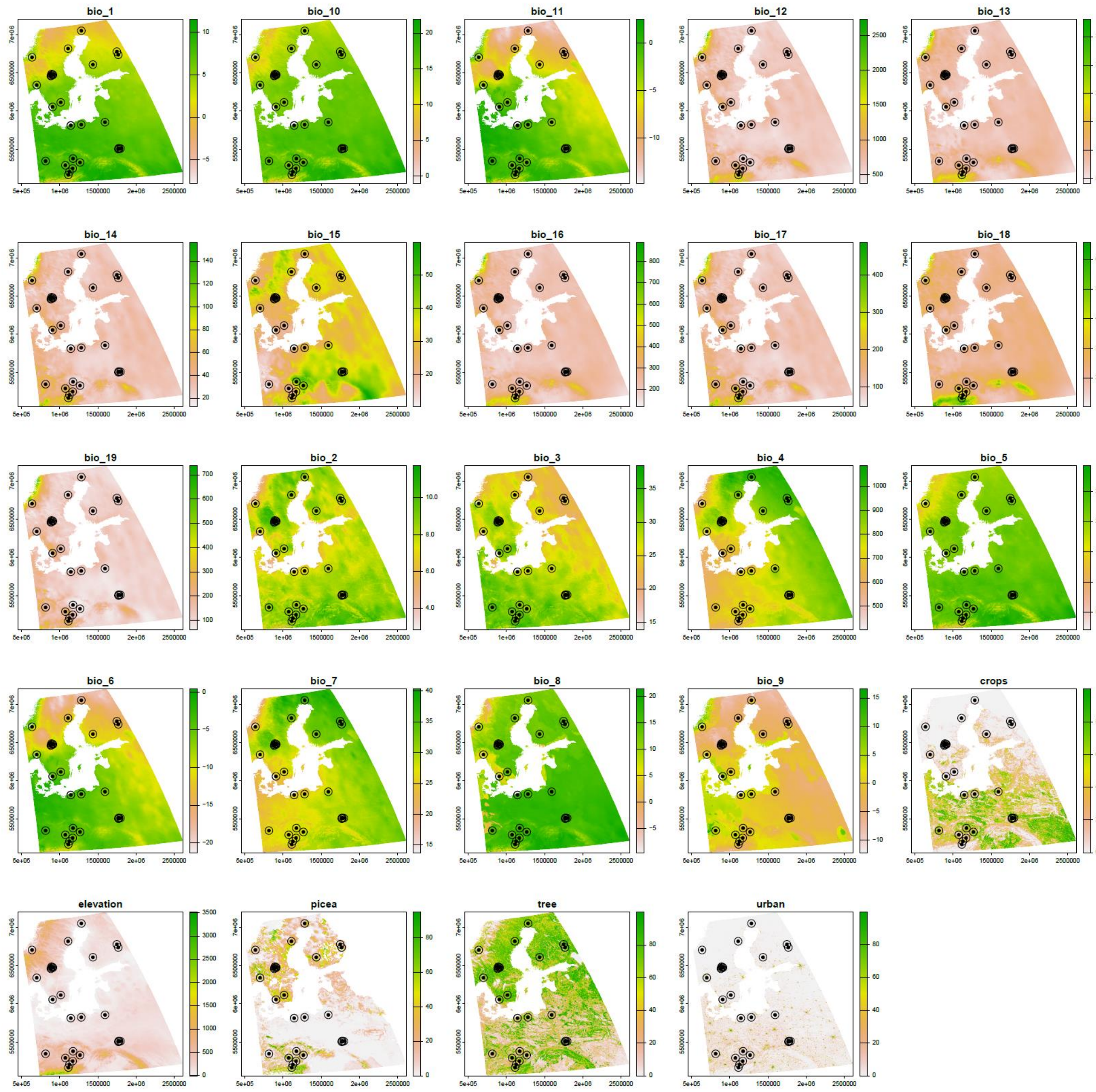
bio_1
bio_10
bio_11
bio_12
bio_13
bio_14
bio_15
bio_16
bio_17
bio_18
bio_19
bio_2
bio_3
bio_4
bio_5
bio_6
bio_7
bio_8
bio_9
crops
elevation
picea
tree
urban

Figure S15 Genotype-environmental association (GEA) analysis for *Ips typographus* populations collected throughout Europe. Variance explained by all PCs. and loadings of environmental variables in the first two principal components (see Table S12 for exact values). BIO1 (Annual Mean Temperature), BIO2 (Mean Diurnal Range), BIO3 (Isothermality), BIO4 (Temperature Seasonality), BIO5 (Max Temperature of Warmest Month), BIO6 (Min Temperature of Coldest Month), BIO7 (Temperature Annual Range), BIO8 (Mean Temperature of Wettest Quarter), BIO9 (Mean Temperature of Driest Quarter), BIO10 (Mean Temperature of Warmest Quarter), BIO11 (Mean Temperature of Coldest Quarter), BIO12 (Annual Precipitation), BIO13 (Precipitation of Wettest Month), BIO14 (Precipitation of Driest Month), BIO15 (Precipitation Seasonality), BIO16 (Precipitation of Wettest Quarter), BIO17 (Precipitation of Driest Quarter), BIO18 (Precipitation of Warmest Quarter), and BIO19 (Precipitation of Coldest Quarter).

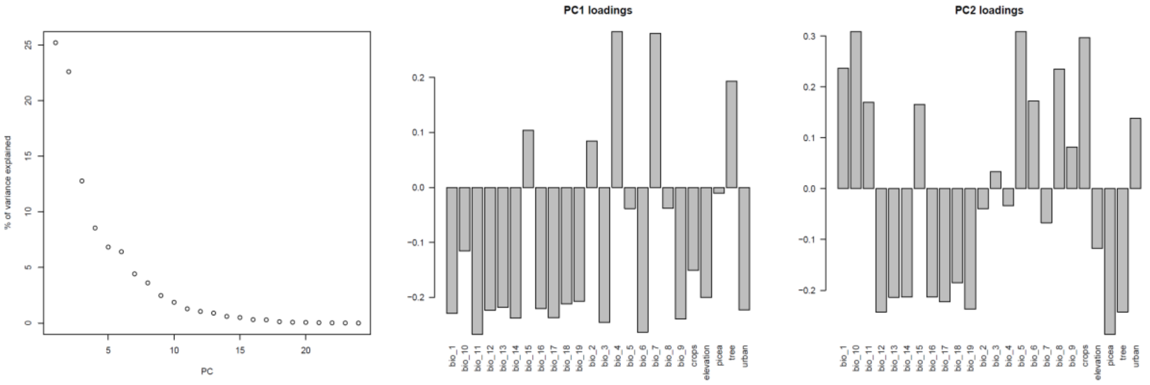

Figure S16 Mutation load analysis in *Ips typographus*.

The ratio of nonsynonymous to synonymous nucleotide diversity (π_N/π_S) computed for 200 kb windows along collinear parts of the genome (COL) and different inversion haplotypes (blue: major haplotype; yellow: minor haplotype; green: haplotype produced by double crossover events between minor and major haplotypes). n = 4 to 55 200kb windows per inversion haplotype and n = 531 for COL. The lower and upper box hinges correspond to the first and third quartiles, whiskers show 1.5 × the inter-quartile range.

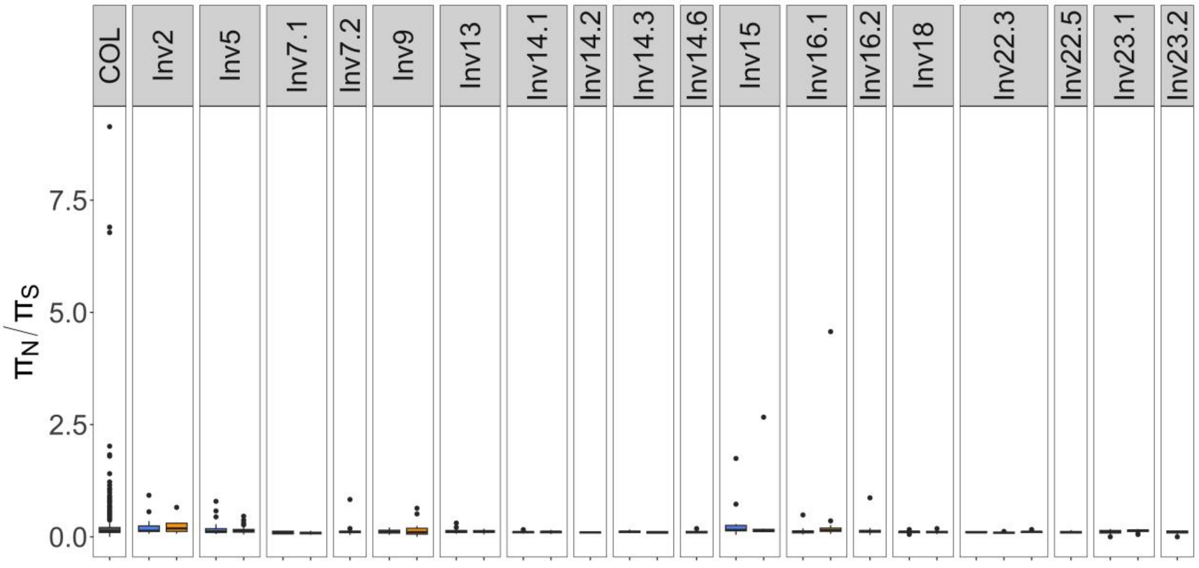

Supplement: evae263_Supplementary_Data [file evae263_supplementary_data.pdf]
